# Fast Amide Bond Cleavage Assisted by a Secondary Amino and a Carboxyl Group—A Model for yet Unknown Peptidases?

**DOI:** 10.3390/molecules24030572

**Published:** 2019-02-05

**Authors:** Igor V. Komarov, Aleksandr Yu. Ishchenko, Aleksandr Hovtvianitsa, Viacheslav Stepanenko, Serhii Kharchenko, Andrew D. Bond, Anthony J. Kirby

**Affiliations:** 1 Institute of High Technologies, Taras Shevchenko National University of Kyiv, Vul. Volodymyrska 64/13, 01601 Kyiv, Ukraine; 2 Enamine Ltd., Vul. Chervonotkatska 78, 02094 Kyiv, Ukraine; ischa1986@ukr.net (A.Y.I.); cayenestar@gmail.com (A.H.); v.stepanenko@enamine.net (V.S.); serxioharchenko@ukr.net (S.K.); 3 University Chemical Laboratory, Cambridge CB2 1EW, UK; adb29@cam.ac.uk

**Keywords:** amide hydrolysis, model compound, intramolecular catalysis, twisted amide, protease, intein

## Abstract

Unconstrained amides that undergo fast hydrolysis under mild conditions are valuable sources of information about how amide bonds may be activated in enzymatic transformations. We report a compound possessing an unconstrained amide bond surrounded by an amino and a carboxyl group, each mounted in close proximity on a bicyclic scaffold. Fast amide hydrolysis of this model compound was found to depend on the presence of both the amino and carboxyl functions, and to involve a proton transfer in the rate-limiting step. Possible mechanisms for the hydrolytic cleavage and their relevance to peptide bond cleavage catalyzed by natural enzymes are discussed. Experimental observations suggest that the most probable mechanisms of the model compound hydrolysis might include a twisted amide intermediate and a rate-determining proton transfer.

## 1. Introduction

**Scheme 1:**
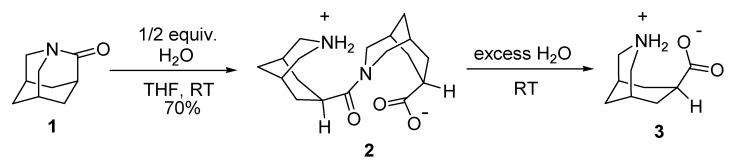
Formation and hydrolysis of amide **2**.

Hydrolysis of the 1-azatricyclo[3.3.1.1^3,7^]decan-2-one (**1**), which contains an ultimately twisted and *N*-pyramidalized amide bond, was reported to be very fast and accompanied by the formation of the “dimer” (compound **2**) and higher oligomers [1]. The use of a half equivalent of water under certain conditions led almost exclusively to formation of **2** (Scheme 1). Apparently, the product of the initial hydrolysis of **1**—the amino acid **3**—was immediately *N*-acylated by unreacted **1**, which possesses an extremely electrophilic carbonyl group. In contrast to the twisted and therefore highly reactive amide **1**, the dimer **2** has an essentially unconstrained amide bond: yet we found that it reacted with excess of water at a comparable rate, also giving the amino acid **3** (Scheme 1) [1]. Such a fast amide hydrolysis is unusual; therefore, we decided to study the compound **2** in more detail.

**Figure 1:**
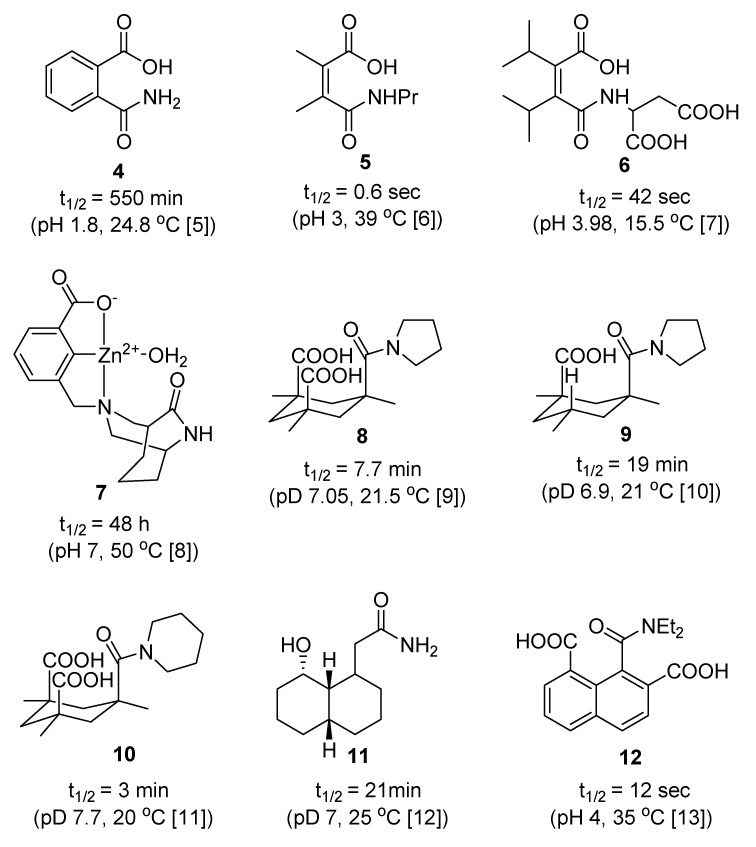
Selected compounds reported to undergo unusually fast amide bond hydrolysis in buffered water under mild conditions (indicated in brackets). Values of the half-life, calculated using the measured pseudo-first order rate constants *k_obs_* are listed for each compound.

Examples of amides hydrolysable under mild conditions are rare, but not unknown. Apart from **1** and other similar systems featuring highly deformed amide bonds (see the reviews [2,3,4]), there are compounds where fast amide bond hydrolysis was suggested to be facilitated by neighboring group assistance (Figure 1 lists some representative examples, [5,6,7,8,9,10,11,12,13]).

Compounds like **1** and **4**–**12** should not be regarded simply as chemical curiosities. In fact, most of them were deliberately designed and synthesized to obtain valuable information on the fundamental properties of the amide bond, and to use this information in unraveling the mechanisms of biologically relevant processes involving amide formation and cleavage: particularly mechanisms of protein cleavage catalyzed by proteolytic enzymes. Perhaps most studied in this regard are molecules mimicking the function of the side-chain carboxyl groups in the active sites of aspartic proteases. The first systematic studies with such molecules, later named enzyme models [14], were undertaken back in the 1950s [5,15]. In the following decades many more similar models were discovered. It was demonstrated that one or two carboxyl groups in close proximity to the amide function (as in compounds **4**–**6**, **8**–**10**, **12**) catalyzed the amide bond hydrolysis with rates comparable to those of the corresponding enzymatic transformations. Similar work was also published on models of metalloproteases (exemplified here by compound **7**), and serine/cysteine proteases (e.g., compound **11**). Although the validity of the enzyme models and their relevance to the natural enzyme mechanisms has been extensively criticized and re-examined (see, for example, [10,11,16,17], there is no doubt that they made important contributions to our understanding of the natural catalytic systems, and will do so in the future.

Moreover, reactive model amides like compounds **1** and **4**–**12**, together with natural systems, inspired many chemists to design selective and efficient non-enzymatic transformations of the amide bond, not limited to hydrolysis. For example, twisting around the N−C bond, as a general concept for amide bond activation was ingeniously utilized for the diverse and unprecedented functionalization of amides. Such twisting can be easily achieved either by formation of bridgehead lactams [18], or even by modifying acyclic amides [19] without resorting to polycyclic scaffolds. Notable are the metal-catalyzed N−C cross-coupling reactions of the twisted amides, impossible for unconstrained compounds (see recent reviews on this subject, [2,20,21,22]. Activation by neighboring groups was also used for developing selective amide group cleavage [23] and amine group protection strategies [24,25]. There has also been significant progress towards selective biomimetic metal-ion promoted amide bond cleavage, mimicking metalloproteases and amidohydrolases [23,26,27,28,29,30]. It should be noted, however, that efficient artificial catalytic (in particular, organocatalytic) amide bond transformations characteristic of proteases presents a challenge so far unachieved [31].

In view of the above, compound **2** might be regarded as a promising model compound, where the observed fast amide bond hydrolysis under mild conditions could provide new information about the intrinsic reactivity of the amide linkage and its possible catalytic cleavage.

One might hypothesize that the observed fast hydrolysis of **2** is facilitated by both carboxyl and secondary amine groups flanking the amide bond. In this paper, we report experimental evidence for this hypothesis, and also discuss the hidden potential of this model system in the study of biochemical transformations and the design of artificial catalytic systems.

## 2. Results

We first wanted to know if the presence of only one functional group, either the amino group or the carboxylate in the structural context of the amide **2** is sufficient for the fast hydrolysis of the amide bond. The literature data (e.g., on **5** [6], **9 [10]**, as well as on the compound described in [32]) clearly demonstrated that a single carboxyl group, if properly placed, can catalyze very fast amide bond hydrolysis intramolecularly, presumably through the proton transfer to the amide bond oxygen and subsequent nucleophilic attack at the N-CO bond carbon [6]. Involvement of the basic nitrogen of a neighboring pyrimidine substituent in the hydrolytic cleavage of the amide bond has also been well documented [33].

We synthesized two isostructural analogues of **2**, compounds **13** and **14**, one lacking the secondary amino group (bearing the –CH_2_– fragment instead), and the other possessing no carboxyl group (with –CH_2_– in place of –CH(COOH)–).

**Scheme 2:**
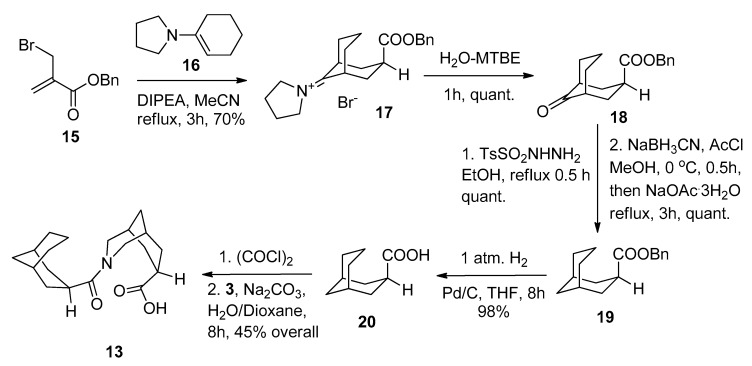
Synthesis of model compound lacking the amino group.

Compound **13** was prepared via the bicyclic carboxylic acid **20**, synthesized by the method first published in [34] and further refined by us [35]. The acid **20** was converted to the corresponding acid chloride and coupled under the Schotten–Baumann reaction conditions with amino acid **3** (Scheme 2).

**Scheme 3:**
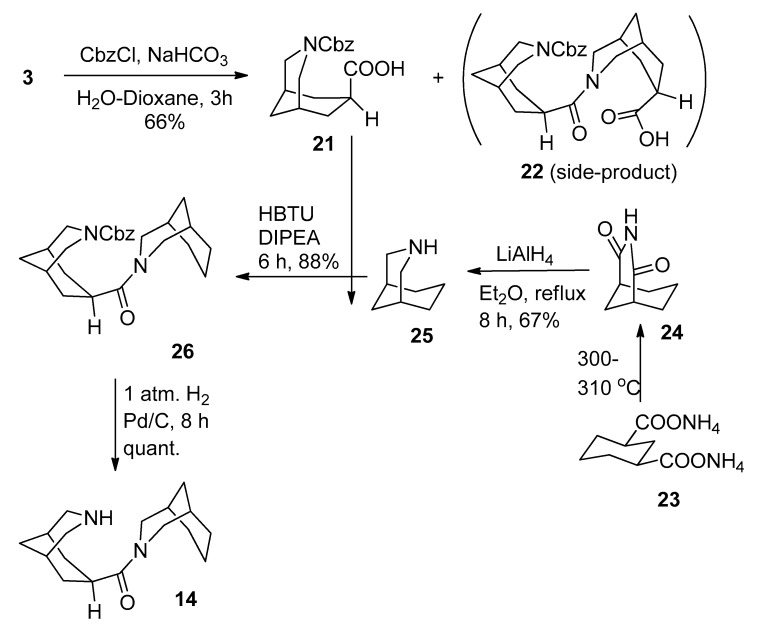
Synthesis of model compound lacking the carboxyl group.

The key intermediate in our synthesis of **14** was the bicyclic amine **25**, which was obtained following the steps described in [36,37] (Scheme 3). N-Cbz-protected amino acid **21** was prepared from **3** (we observed also formation of the side-product **22** during the protection) and coupled with **25** using a standard peptide coupling protocol. Cleavage of the Cbz-protecting group by hydrogenolysis afforded compound **14**.

**Figure 2:**
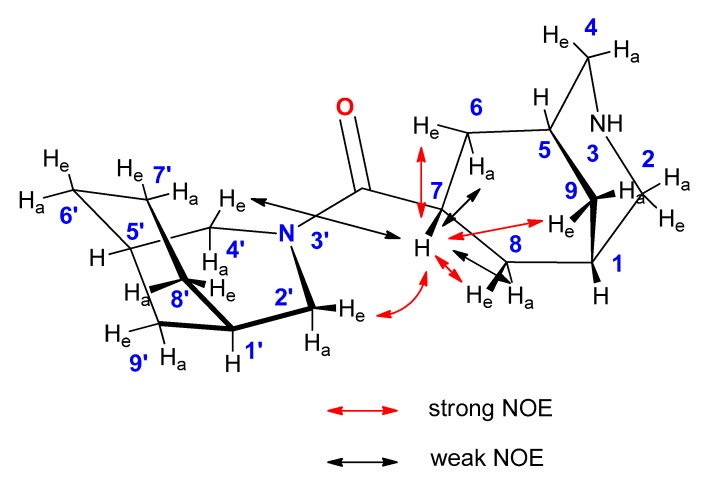
NOE correlations for 7-CH ^1^H-NMR peak in the 2D-NOESY spectrum of compound **14**.

The structure of the amide **14** was confirmed by comprehensive analysis of its 2D-NMR spectra. The key NOESY correlations, which prove the structure of **14** in solution are shown in Figure 2 (see also the Supporting Information).

**Figure 3:**
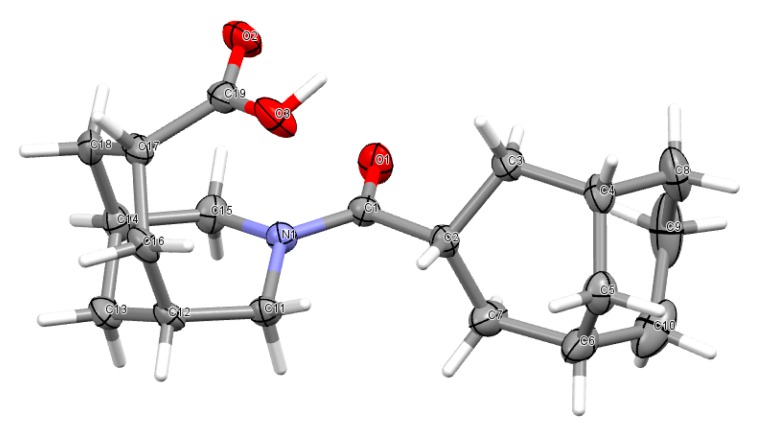
Molecular structure of **13** (ellipsoids are drawn at the 50% probability level).

Compound **13** gave single crystals suitable for X-ray structure determination, which showed that the carboxyl group is indeed in close proximity to the amide bond (Figure 3) and might facilitate hydrolysis: as for example, in the known compound **9 [10]**. However, neither **13** nor **14** was hydrolyzed at a measurable rate under the conditions used in [1] (0.084M in acetonitrile-d_3_ solution in the presence of five equivalents of water), in which compound **2** was reported to be hydrolyzed within days. We noted only very slow changes in the solutions of **14** in the presence of a large excess of water (on standing in a CD_3_CN-D_2_O mixture, 1:1 *v\v* at 25 °С): where formation of insoluble, presumably polymeric material was observed within several weeks. These results are also in agreement with the reported fact [1] that if the carboxyl group in compound **2** is reduced to CH_2_OH, the resulting amino alcohol also withstands hydrolysis even during prolonged treatment with excess water. Therefore, one might conclude that the *amino and carboxyl groups* act together to facilitate the hydrolysis of amide **2**.

**Scheme 4:**
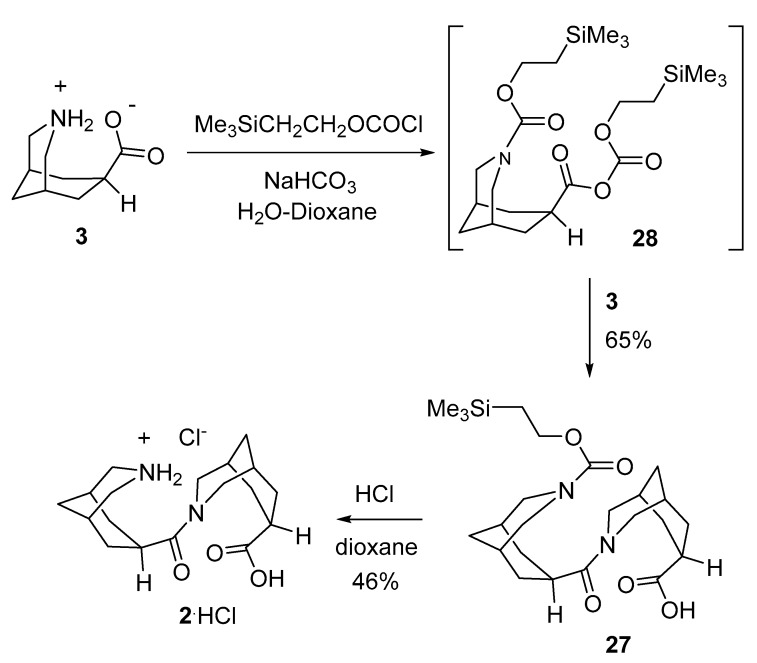
Alternative synthesis of amide **2** (hydrochloride).

In order to elucidate possible mechanisms for the intramolecular catalysis of the amide bond in **2**, we decided to obtain this compound as a hydrochloride by a route different from that shown in Scheme 1 in order to study the rates of its reaction with water in buffers at different pH. We reasoned that the hydrochloride salt would be more stable than the zwitterionic form of **2** obtained directly from **1**, enabling us to purify the compound. The synthetic approach was based on our observation (above) that treatment of the amino acid **3** with CbzCl gave not only the expected protected compound **21**, but also the dimer **22** as a side-product (Scheme 3, in parentheses). Using Me_3_SiCH_2_CH_2_OCOCl as protecting reagent and varying the reaction conditions, we obtained the dimer **27** in a reasonable yield. Most probably, formation of **27** proceeded through the activated intermediate **28**, which reacted with the starting amino acid (Scheme 4). Alternatively, compound **27** could be formed through the twisted amide **1**, formed upon activation of the carboxyl group. The twisted amide could acylate unreacted **3** (as shown in Scheme 1), and the dimer **2** produced could then be trapped by the N-protecting reagent. Treatment of **27** with dry HCl in dioxane afforded the hydrochloride **2**·HCl.

Compound **2**·HCl indeed turned out to be quite stable and crystallized from the reaction mixture after the deprotection of **27** upon addition of diethyl ether. The purity of the obtained **2**·HCl was sufficient to study its hydrolysis in different D_2_O buffers (acetate, pD 3.81 and 4.85; phosphate, pD 6.68 and 7.95; carbonate, pD 9.45 and 10.68). Progress of the hydrolysis was monitored by ^1^H-NMR using unambiguously assigned peaks for the starting compound and its hydrolysis product.

**Figure 4:**
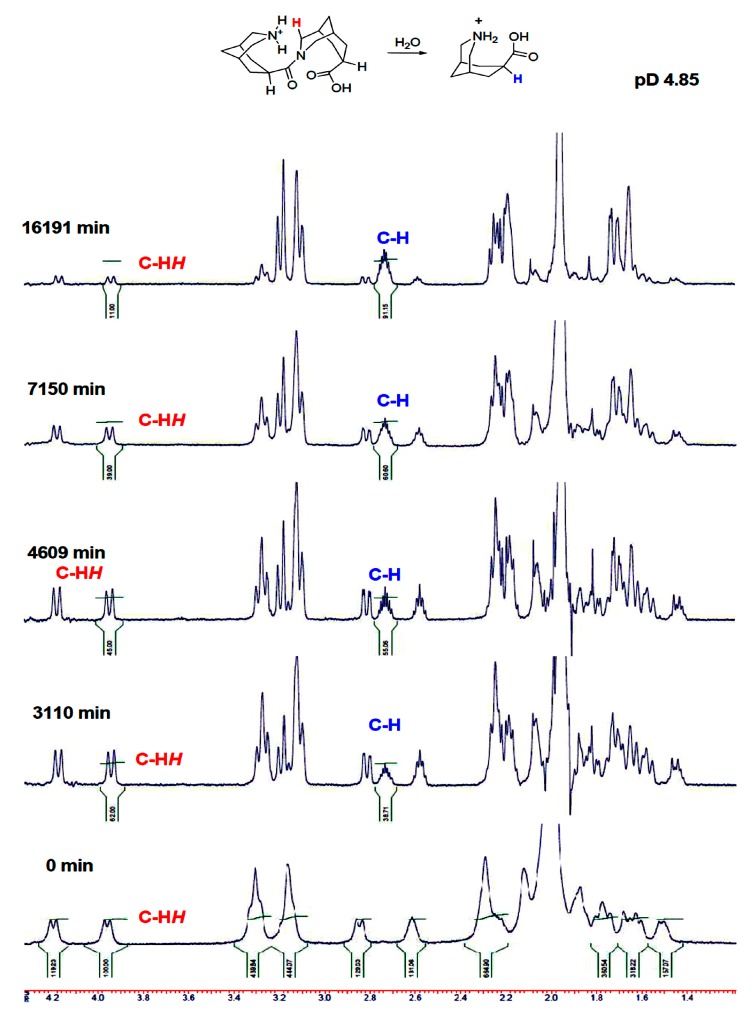
Representative ^1^H-NMR spectral data set for hydrolysis of **2**·HCl in acetate D_2_O buffer, pD 4.85, 23 °C.

In the acidic acetate buffers (pD 3.81 and 4.85) and in phosphate buffer at pD 6.68, the **2**·HCl hydrolysis product—the amino acid **3**—appeared slowly in the ^1^H- and ^13^C-NMR spectra while the starting amide peaks disappeared. A typical ^1^H-NMR data set for the hydrolysis in an acetate buffer is illustrated in Figure 4. The kinetic parameters obtained for the hydrolysis in the acidic-neutral buffers are listed in Table 1 (see also the Experimental section and Supporting Information for the details). As can be seen from the data in Table 1, the pseudo-first order rate constant ***k_obs_*** of the hydrolytic amide bond cleavage in **2** gradually increases with the pD values in this pD range, with the maximum of 1.849 × 10^−4^ min^−1^ (half-life ~62.5 h, 23 °C) in phosphate buffer at pD 6.68.

Hydrolysis of **2**·HCl in alkaline buffers (phosphate at pD 7.95, carbonate at pD 9.45 and 10.68) was found to become slower as the pD increased. The kinetic data obtained for this pD range are shown in Table 2.

**Figure 5:**
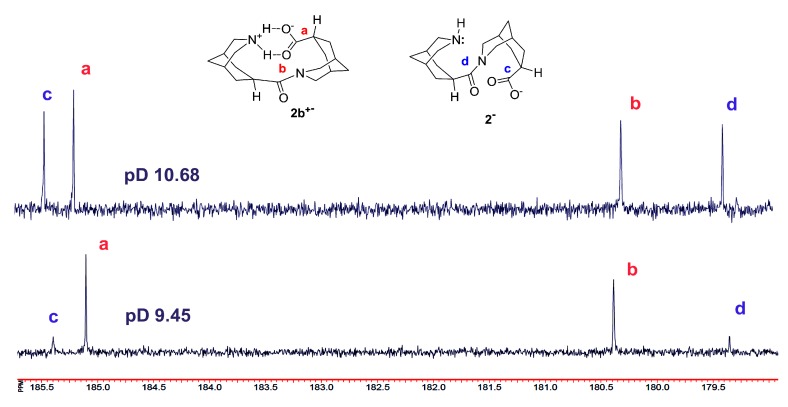
Fragments of ^13^C-NMR spectra of compound **2** in different buffers containing peaks of the carbonyl carbon atoms.

Interestingly, at pD 9.45 and 10.68 we observed a duplicated set of the ^1^H- and ^13^C-NMR signals corresponding to the starting amide, suggesting the presence of its two forms in the solutions (Figures S33–S36 in the Supplementary Materials). These two forms were observed immediately after the compound was dissolved (the equilibrium between the forms established faster than we could run the ^1^H-NMR spectrum, ~2 min), and their ratio remained unchanged during the whole period of the kinetic measurements (~2 months). However, the ratio between the forms depended on the pD of the solutions. The form which was dominant at the pD 9.45 had the chemical shifts of the NMR signals close to those observed for the **2**·HCl in acidic buffers; at the pD 10.68, both forms gave corresponding signals of almost equal intensity; to explain these observations, we suggest the existence of the structures **2b^+−^** and **2^−^** in the alkaline solutions (Figure 5).

**Figure 6:**
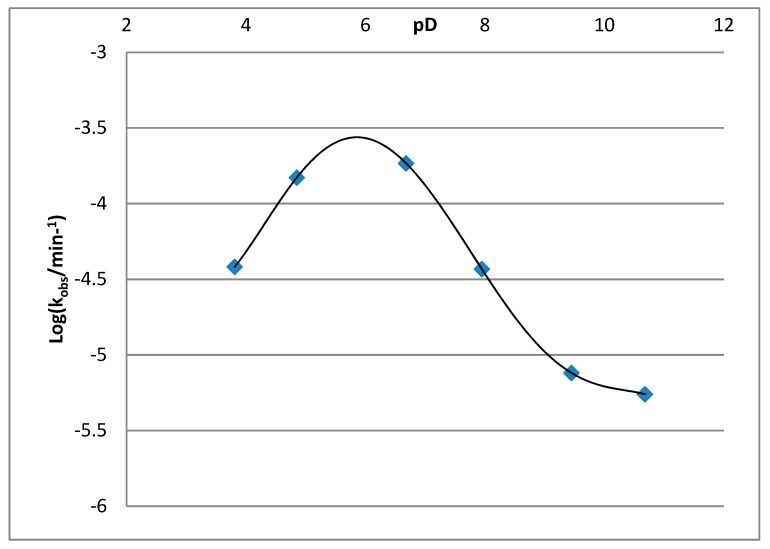
pD-rate profile for the hydrolysis of **2**·HCl.

Figure 6 summarizes the kinetic data, showing the pD–rate profile of the amide **2** hydrolysis in D_2_O. The profile is bell-shaped, with the rate maximum at around neutral pD at 23 °C.

## 3. Discussion

**Figure 7:**
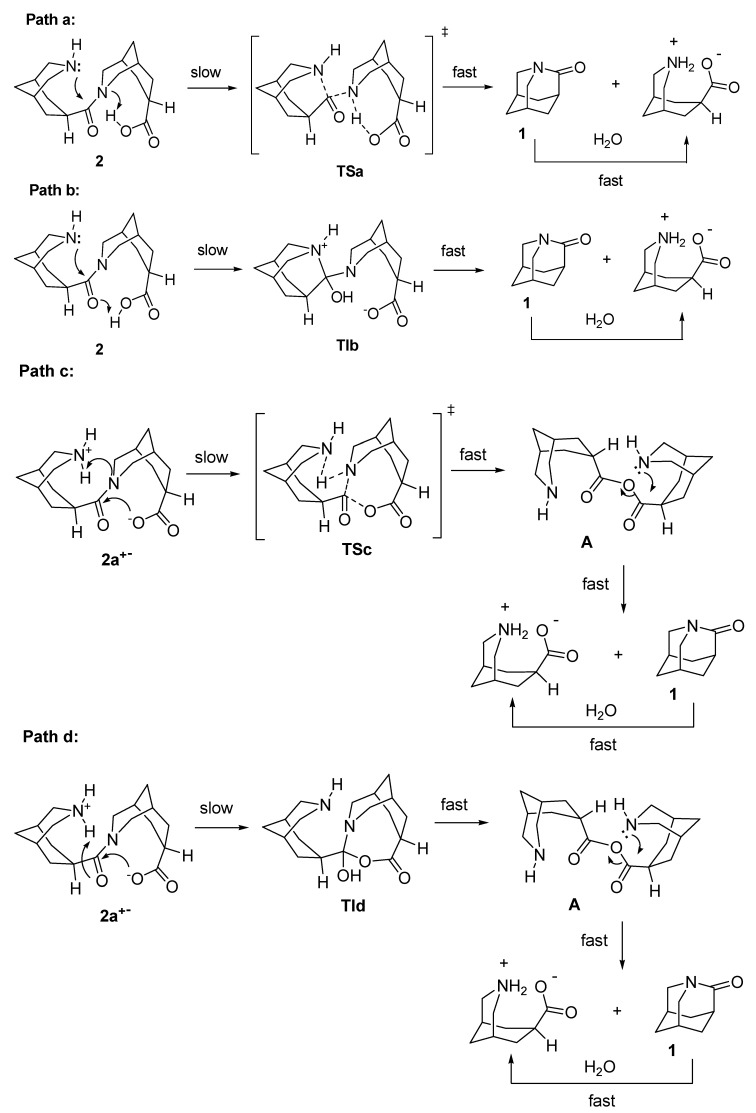
Possible mechanisms of the hydrolysis of the amide **2**.

The bell-shaped rate profile for the hydrolytic cleavage of the amide **2** confirmed our suggestion made using the model molecules **13** and **14**: that both amino and carboxyl groups are involved in the hydrolysis. On the basis of our experimental data, one can suggest the equilibria between differently protonated **2** in different buffers, as schematically illustrated in Figure 7. Evidently, the basic nitrogen in **2** is protonated and “switched off” from intramolecular assistance of the amide bond hydrolysis at low pDs (where species **2^+^** dominate). Similarly, deprotonation of the COOH group “switches off” its involvement in the reaction at high pDs (the equilibria shifting to unreactive **2^−^**). There is an alternative explanation of the pD rate profile—see the discussion of possible hydrolysis mechanisms below—but in all cases the amino and carboxyl groups should act synergistically.

**Figure 8:**
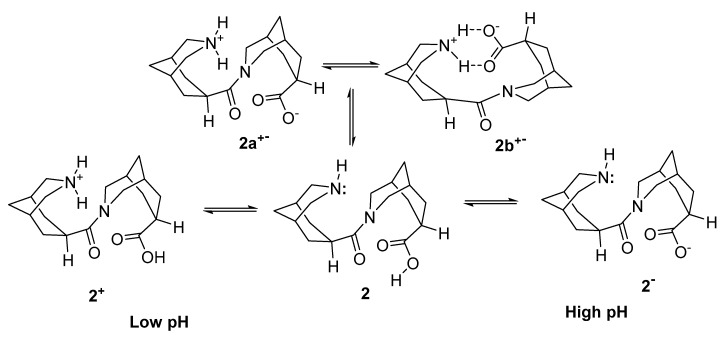
Equilibria established in solutions of **2** at different pH.

At the high pDs, another reason for the slowdown of the reaction might be epimerization at the carbon atom next to the carboxyl group. The racemization could also explain the doubling of the signals in the solution of **2** in the alkaline buffers. However, we can exclude this possibility: the NMR signal-doubling is observed almost immediately after dissolving the amide **2** in the buffers, and the ratio of the species observed in the NMR spectra is dependent on the pD, but not on time. Epimerization in similar systems is known to proceed much more slowly and under harsher conditions, leading to almost complete conversion of the endo-COOH epimer to the exo-COOH epimer [35]. Observation of the two species in the spectra of **2** might be explained by the slow (on the NMR time scale) exchange between the species **2b^+−^** and **2^−^** at high pDs (Figure 8). This could be confirmed by the fact that one of the species (content increasing with pD, so assigned to **2^−^**) has the carboxyl group ^13^C-NMR resonance at lower field then the other species (tentatively assigned to **2b^+−^**, Figure 8), in line with literature data reporting the downfield shift for the COOH ^13^C-NMR peak upon deprotonation [38].

**Figure 9:**
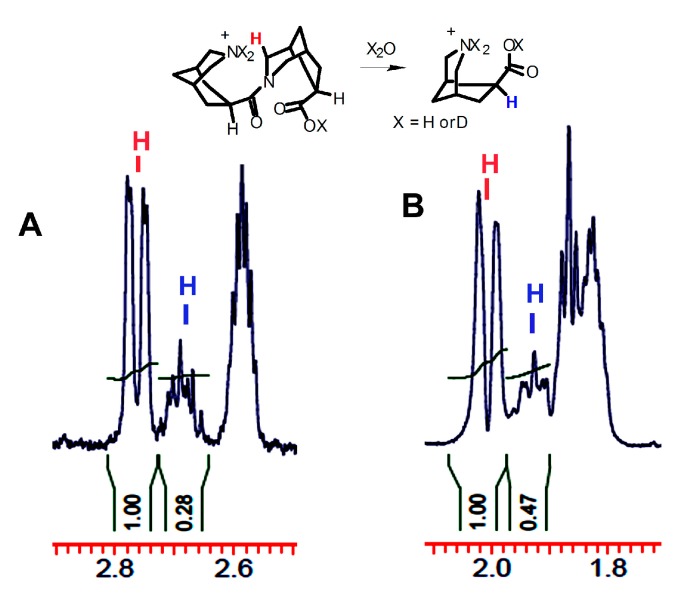
Characteristic regions of the ^1^H-NMR spectra illustrating the hydrolysis rates of **2**·HCl in D_2_O (**A**) and H_2_O (**B**). After twelve days of reaction.

Two types of possible mechanisms of the hydrolysis of **2** can be suggested, if both the amino and carboxyl functions are involved. These mechanisms (which we name *N*- and *O*-mechanisms in order to distinguish them in the following discussion) differ by the roles played by the two functional groups in intramolecular catalysis. The N-mechanism, realizable either through **path a** or **b** (Figure 7) involves a nucleophilic attack of the basic nitrogen atom on the amide bond carbon while the COOH group acts as a general acid. It might be concerted (**path a**) or involve formation of a tetrahedral intermediate (**TIb**, **path b**), but in both cases should result in formation of the twisted amide **1**. Although we did not observe **1** in the reaction mixtures at any pDs by NMR, we know that its hydrolysis will be extremely fast under the reaction conditions used [1], excluding the accumulation of **1** in observable concentrations. The O-mechanism (concerted **path c** or stepwise **path d**, Figure 7) suggests that the amino and the carboxyl group swap roles in the first step of the reaction: the deprotonated COOH is now the nucleophile and the protonated amino group acts as the general acid. This possible mechanism would involve formation of the anhydride intermediate **A** (apart from the tetrahedral intermediate **TId** in the **path d**). Degradation of **A** might again involve the nucleophilic amino group attack at one of the anhydride carbonyls and formation of the twisted amide **1**. In all cases the limiting step is the first; the following transformations should be fast—explaining why no intermediates were detected by NMR spectroscopy in the reaction mixtures. In order to prove that the rate limiting step of the hydrolysis (at least at low pH) involves a proton transfer, we set up two hydrolysis reactions of **2**·HCl running in parallel with all the conditions but one identical: the solvent for one being H_2_O, for the other, D_2_O. NMR monitoring of the reaction rates revealed a well-defined isotope effect: the hydrolysis in H_2_O being ca 1.3–1.6 times faster than in D_2_O. Figure 9 illustrates the faster appearance of the hydrolysis product in the ^1^H-NMR spectra of the reaction mixtures run in the course of the experiment.

The fact that we observed none of the possible intermediates makes it difficult to choose between **paths a-d** based only on our experimental data.

If the amine nitrogen is indeed involved as the nucleophilic center in the intramolecular catalysis of the amide bond cleavage in **2**, this raises an intriguing question: why does nature not utilize nitrogen-based nucleophilic centers in enzymatic hydrolytic cleavage of the amide bond? None of the currently known peptidases (hydrolases acting on amide bonds in protein backbones [39]) utilize an N-nucleophilic attack as a part of their catalytic mechanism [40]. Ubiquitous and long-studied serine and cysteine proteases feature the side-chain –OH or –SH nucleophilic groups in their active sites. Similarly, the threonine –OH group is thought to be involved as a nucleophile in relatively recently discovered threonine proteases [41]. The catalytic mechanisms proposed for both aspartic and metalloproteases involve nucleophilic attack of a water molecule at the scissile amide bond. Finally, the rare glutamic proteases were also suggested to utilize a water molecule as the nucleophile [42]. Even if there are as yet undiscovered “XX-peptidases” (XX designating an N-nucleophile in the active site), they undoubtedly must be rare.

**Figure 10:**
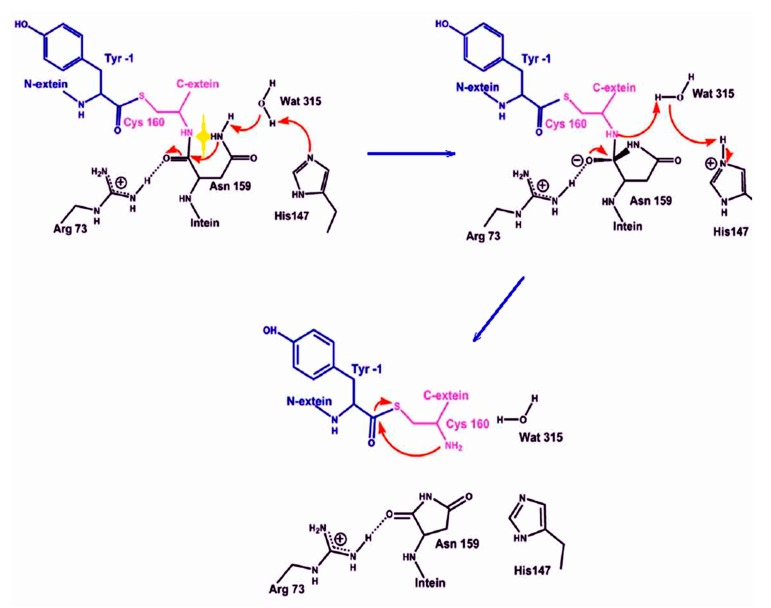
A part of the proposed intein SspDnaE splicing mechanism involving the Asn159 nucleophilic attack (marked with the yellow star) at the nearby amide bond carbonyl. Reprinted from [46] with permission from Elsevier.

In fact, there are known proteolytic enzymes that cleave peptide bonds with active participation of a nucleophilic nitrogen atom. These enzymes are not peptidases but rather lyases, as their action does not include hydrolysis. The N-nucleophile thought to play the key role in the amide bond cleavage is the nominally non-nucleophilic amide nitrogen of the asparagine side chain. Named asparagine peptide lyases, these enzymes were assigned to a “seventh catalytic type of proteolytic enzymes” [43]; many families of this enzyme type are now known. Typical examples are inteins, the protein segments able to mediate their own scission from larger proteins [44,45]. This process, known as protein splicing, involves cleavage of the two amide bonds flanking the inteins, producing the intein-removed proteins built from two remaining N- and C-extein fragments. Most intein-containing proteins have a conserved asparagine residue at one of the amide-bond cleavage sites. In the key step of the splicing, the asparagine amide is activated for intramolecular nucleophilic attack at its own peptide bond, leading to its cleavage. The chain of molecular events, a part of the mechanism proposed for splicing an intein (SspDnaE) [46] is illustrated in Figure 10.

Details of the activation of the Asn side chain amide for the nucleophilic attack are not completely understood. This could involve its tautomerization with the formation of a more reactive imidate species [47] or, alternatively, twisting the amide bond, which is known to enhance its nucleophilicity [48]. Interestingly, a mechanism involving the twisted asparagine amide group was proposed for oligosaccharyltransferases, the membrane protein complexes which catalyze asparagine-linked glycosylation [49]. Whatever the mechanism of the amide nucleophile activation, it is clear that nature avoids strong amine nucleophiles in the active sites of the proteolytic enzymes. They are present in a latent form (amide group of Asn) and only generated during the catalysis. It cannot be due to low availability of active *N*-nucleophiles in proteins. The lysine amino group, for example, acts as a nucleophile in ribulose 1,5-bisphosphate carboxylase (Rubisco), the most abundant enzyme on Earth [50]. In the catalytic protein splicing described above, where amide bonds are cleaved, however, the action of the asparagine-derived active nucleophile results in destruction of the enzyme itself. One might even question whether the splicing can be considered as an enzymatic process [43]. The prospect of self-destruction could be one of the reasons why natural proteases evolved without *N*-nucleophiles in the active site.

**Figure 11:**
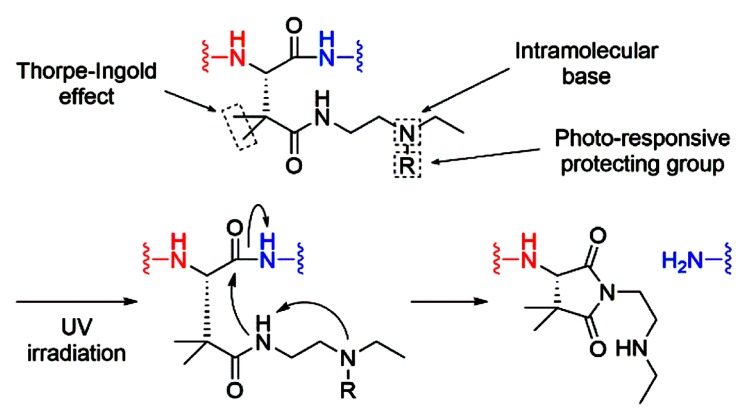
Photoinduced peptide bond cleavage inspired by natural protein splicing. Reprinted with permission from [51]. Copyright 2016 American Chemical Society.

Our model compound **2** demonstrates that one possible way to avoid self-destruction could be structural constraint holding the intermediate *N*-acylated species in a twisted conformation, before a water molecule arrives and completes the hydrolysis cycle. There are no reasons to believe that this is impossible in natural catalytic systems, therefore, one might also believe that “XX-peptidases” are yet to be discovered. In any case, in seems worthwhile to design artificial catalytic systems exploiting the *N*-nucleophiles in the catalytic processes. Such systems could be inspired by known natural enzymes or by model molecules as described in this paper. One important step towards this goal has already been reported by Otaka et al. [51]. They developed a photo-responsive amide cleavage device, mimicking the intein-mediated protein splicing. According to their approach, photo-triggered liberation of the secondary amino group on a modified asparagine fragment activates the asparagine amide for the nucleophilic attack that subsequently cleaves the adjacent peptide bond, as illustrated in Figure 11.

## 4. Materials and Methods

### 4.1. General

Solvents were purified according to standard procedures [52]. Melting points were measured on an automated melting point system and are uncorrected. Analytical TLC was performed using Polychrom SI F254 plates. Column chromatography was performed using silica gel (230–400 mesh) as the stationary phase. ^1^H, ^13^C NMR and all 2D NMR spectra were recorded at 499.9 or 400.4 MHz for protons and 124.9 or 100.4 MHz for carbon-13. Chemical shifts are reported in ppm downfield from TMS (^1^H, ^13^C) as an internal standard. Mass spectra were recorded either on an Agilent 1100 LC/MSD SL (Agilent, Santa Clara, CA, USA) instrument by chemical ionization (CI) or on a GCMS instrument with electron impact ionization (EI). CHN-analysis was done on an Elementar VarioMICRO Cube analyzer (Elementar, Langenselbold, Germany).

### 4.2. Synthetic Procedures

*3-Endo-bicyclo[3.3.1]nonane-3-carboxylic acid* (**20**). This known compound was prepared starting from 1-cyclohexenylpyrrolidine (**16**) and 2-bromomethyl-acrylic acid benzyl ester (**15**) on a 0.2 mol scale in 69% overall yield following the procedures described in [36] for the analogue, 7-endo-3-(tert-butoxycarbonyl)-3-azabicyclo [3.3.1]nonane-7-carboxylic acid. The ^1^H-NMR spectrum of the product corresponded to that described in [53]. White crystals, m.p. 126 °C (lit. 126.5–127 °C [52]).^1^H-NMR (δ, CDCl_3_, 500 MHz): 11.78 (broad s, 1H, COOH), 2.48 (h, 6.0 Hz, 1H, 3-H), 1.9–2.1 (m, 4H), 1.6–1.7 (m, 1H), 1.55 (d, 13.5 Hz, 1H), 1.25–1.45 (m, 7H), 1.09 (d, 13.5 Hz, 1H). ^13^C-NMR (δ, CD_3_Cl, 124.9 MHz): 183.27 (COOH), 36.56, 32.50, 28.74, 28.48, 24.43, 15.55. LC/MS (CI, neg. scan): *m/z* = 167.2; (CI, pos. scan): *m/z* = 169.2. Anal. Calc. for C_10_H_16_O_2_: C, 71.39; H, 9.59. Found: C, 71.35; H, 9.55.

*3,7′-Endo-3′-(bicyclo[3.3.1]non-3-ylcarbonyl)-3′-azabicyclo[3.3.1]nonane-7′-carboxylic acid* (**13**). Compound **20** (266 mg, 1.6 mmol) was dissolved in dry dichloromethane (0.5 mL) and mixed with oxallyl chloride (5 mg, 4 mmol). The mixture was stirred under protection from air moisture for 8 h. Then the volatile products were removed under vacuum (0.01 mm Hg, 40 °C, 4 h). The residue was dissolved in dioxane (1 mL) and added dropwise to a solution of the amino acid **3** (prepared as described in [1], 244 mg, 1.44 mmol) and Na_2_CO_3_ (0,763 g, 7.2 mmol) in water (1 mL) under stirring and cooling by an ice bath. The mixture was stirred for 8 h at ambient temperature, evaporated on a rotary evaporator, and the residue (the sodium salt of **13**) crystallized from ethanol. The crystallized and air-dried product was dissolved in a minimum amount of water (approximately 0.4 mL) and the solution was acidified by conc. HCl to afford **13** as a white crystalline material. It was filtered and dried in vacuum (0.01 mm. Hg, 40 °C, 4 h). 230 mg, 45% overall yield. Below, the data for the sodium salt of **13** (an analytical sample) are listed. m.p. 233 °C. ^1^H-NMR (δ, D_2_O, 500 MHz): 4.13 (d, 12.5 Hz, 1H), 3.82 (d, 12.5 Hz, 1H), 3.01 (d, 12.5 Hz, 1H), 2.91 (m, 1H), 2.52 (d, 12.5 Hz, 1H), 2.20 (m, 1H), 1.75–2.35 (m, 8H), 1.55–1.75 (m, 3H), 1.15–1.45 (m, 8H), 0.95–-1.10 (m, 3H). ^13^C-NMR (δ, D_2_O, 124.9 MHz): 184.89, 179.73, 52.16, 48.93, 37.80, 32.41, 32.26, 32.18, 28.79, 28.75, 28.26, 28.05, 27.73, 26.35, 25.58, 25.43, 24.39, 24.11, 15.09. LC/MS (CI, neg. scan): *m/z* = 318.2; (CI, pos scan): *m/z* = 320.2. Anal. Calc. for C_19_H_29_NO_3_: C, 71.44; H, 9.15; N, 4.38. Found: C, 71.40; H, 9.13; N, 4.39.

*7′-Endo-3′-[(benzyloxy)carbonyl]-3-azabicyclo[3.3.1]nonane-7′-carboxylic acid* (**21**). Amino acid **3** (169 mg, 1 mmol) and NaHCO_3_ (200 mg, 2.4 mmol) were dissolved in water-dioxane mixture (10 mL, 1:1 *v/v*). To this solution was added benzyl carbonochloridate (carbobenzoxychloride, 0.2 mL) dropwise under stirring. The mixture was stirred for 3 h, and then acidified carefully by 1N HCl till pH 2. The product was extracted with MTBE (3 × 50 mL), the combined extracts were dried over Na_2_SO_4_ and evaporated. The product was purified by column chromatography (silica gel, MTBE as an eluent). Analytical sample was prepared by crystallization from diethyl ether. Yield: 200 mg, 66%. White crystals, m.p. 120 °C. ^1^H-NMR (δ, CDCl_3_, 500 MHz): 10.02 (br s, 1H, COOH), 7.40–7.70 (m, 5H, arom.), 5.12 (br s, 1H), 4.97 (br s, 1H), 4.03 (br s, 2H), 2.92 (d, 11.5 Hz, 2H), 2.60 (br s, 1H), 2.36 (d, 13.0 Hz, 2H), 1.75–2.00 (m, 4H), 1.64 (d, 11.5 Hz, 1H), 1.54 (d, 11.5 Hz, 1H). ^13^C-NMR (δ, CDCl_3_, 124.9 MHz): 178.67, 156.18, 136.47, 128.01, 127.72, 127.47, 66.67, 48.70, 36.96, 30.89, 29.26, 26.21. LC/MS (CI, neg. scan): *m/z* = 302.3; LC/MS (CI, pos. scan): *m/z* = 304.2. Anal. Calc. for C_17_H_21_NO_4_: C, 67.31; H, 6.98; N, 4.62. Found: C, 67.36; H, 6.86; N, 4.60. Chromatographic purification of **21** also yielded a side-product assigned to the structure **22** (6 mg), on the basis of its ^1^H-NMR and MS data.

*7,7′-Endo-3′-({3-[(benzyloxy)carbonyl]-3-azabicyclo[3.3.1]non-7-yl}carbonyl)-3′-azabicyclo[3.3.1]no ane-7′-carboxylic acid* (**22**). m.p. 170 °C: ^1^H-NMR (δ, (CD_3_)_2_SO, 500 MHz): 11.63 (s, 1H, COOH), 7.20-7.50 (m, 5h, arom.), 5.03 (s, 2H, CH_2_Ph), 4.11 (d, 13.0 Hz, 1H), 3.70-3.90 (m, 3H), 2.93 (d, 12.0 Hz, 1H), 2.55–2.75 (m, 3H), 2.32 (m, 2H), 1.35–1.95 (m, 13H), 1.33 (d, 12.0 Hz, 1H), 1.11 (d, 12.0 Hz, 1H), 0.98 (br s, 1H). LC/MS (CI, neg. scan): m/z = 453.4; LC/MS (CI, pos. scan): m/z = 455.2.

*3-azabicyclo[3.3.1]nonane* (**25**), *3-azabicyclo[3.3.1]nonane-2,4 dione* **24** (5 g, 32 mmol, prepared as described in [35]) was dissolved in THF (100 mL) under an argon atmosphere. Lithium aluminum hydride (3.7 g, 98 mmol) was added in portions to the solution, which was then refluxed for 5 h and stirred for an additional 8 h without heating. The excess of the LiAlH_4_ was quenched carefully with water, the product was sublimed to afford **25** as a colorless crystalline product, 2.68 g, 67% yield.

All the spectral data were identical to those described in the literature [54]. m.p. 157 °C. For the use in the next step, the amine was converted to its hydrochloride by dissolving in an excess of 1N HCl, evaporation in vacuum, co-evaporation with water (3 × 20 mL) on a rotary evaporator, drying in a desiccator over P_2_O_5_ for 24 h. White crystals (crystallized from isopropanol), 3-azabicyclo[3.3.1]nonanehydrochloride (**25**·HCl): ^1^H-NMR (δ, (CD_3_)_2_SO, 500 MHz): 9.81 (broad s, 1H, NH), 8.08 (broad s, 1H, NH), 3.14 (d, 13 Hz, 2H), 3.03 (d, 13 Hz, 2H), 1.90–2.10 (m, 3H), 1.40–1.80 (m, 7H). ^13^C-NMR (δ, (CD_3_)_2_SO, 124.9 MHz): 46.40, 29.80, 28.96, 25.22, 19.36. Anal. Calc. for C_8_H_16_ClN: C, 59.43; H, 9.98; N, 8.66. Found: C, 59.40; H, 9.95; N, 8.69.

*7-Endo-benzyl 7-(3′-azabicyclo[3.3.1]non-3′-ylcarbonyl)-3-azabicyclo[3.3.1]nonane-3-carboxylate* (**26**). The Cbz-protected amino acid **21** (95 mg, 0.31 mmol) and DIPEA (81 mg, 0.63 mmol) were dissolved in acetonitrile (1 mL). (2-(1H-benzotriazol-1-yl)-1,1,3,3-tetramethyluronium hexafluorophosphate (HBTU, 128 mg, 0.34 mmol) was added to the solution under stirring, and the stirring continued for 5 min. Then the mixture was combined with a solution of the amine hydrochloride (**25**·HCl, prepared as described above, 61 mg, 0.37 mmol) and DIPEA (73 mg, 0.56 mmol) in acetonitrile (1 mL). The resulting mixture was stirred for 8 h, and then evaporated on a rotary evaporator. The residue was applied to a chromatographic column (silica gel) and pure **26** was eluted with hexane-ethylacetate 2:1 *v/v*). Colorless oil, crystallized on standing, 88% yield (112 mg). An analytical sample was prepared by crystallization from petroleum ether. m.p. 157 °C. ^1^H-NMR (δ, (CDCl_3_, 500 MHz): 7.25–7.45 (m, 5H, arom.), 5.20 and 5.09 (two d, 2H), 4.57 (m, 1H), 4.02 (d, 12.0 Hz, 1H), 3.95 (d, 12.0 Hz, 1H), 3.88 (d, 12.0 Hz, 1H), 3.29 (d, 12.0 Hz, 1H), 2.75–2.85 (m, 3H), 2.74 (broad s, 1H), 1.80–2.15 (m, 7H), 1.50–1.80 (m, 9H), 1.43 (broad s, 1H), 1.26 (m, 1H). ^13^C-NMR (δ, (CD_3_)_2_SO, 124.9 MHz): 177.47, 177.09, 136.55, 128.15, 127.56, 127.36, 50.43, 35.75, 32.80, 30.20, 29.51, 29.00, 27.00, 26.21., 26.20, 25.97. LC/MS (CI, pos. scan): *m/z* = 411.2. Anal. Calc. for C_25_H_34_N_2_O_3_: C, 73.14; H, 8.35; N, 6.82. Found: C, 73.11; H, 8.39; N, 6.80.

*7-Endo-3′-(3-azabicyclo[3.3.1]non-7-ylcarbonyl)-3′-azabicyclo[3.3.1]nonane* (**14**). The Cbz-protected amide **26** (38 mg, 0.1 mmol) was dissolved in methanol (3 mL). Palladium on charcoal (10%, 60 mg) was added to the reaction vessel, which was then filled with hydrogen and the mixture was shaken under 1 atm H_2_ for 8 h. Then the mixture was filtered, evaporated in high vacuum (0.01 mm Hg) without heating. The crude product **14** easily forms the carbonate upon handling on air, therefore it was mixed with 5% aqueous NaOH solution (5 mL) and extracted with dichloromethane (3 × 20 mL) under an argon atmosphere. The combined dichloromethane extracts were evaporated in high vacuum without heating, the residue was dried in high vacuum for 5 h. Yellowish oil, 27.6 mg (quantitative yield). ^1^H-NMR (δ, CD_3_OD, 500 MHz): 4.55 (d, 15.0 Hz, 1H), 4.08 (d, 15.0 Hz, 1H), 3.36 (d, 15.0 Hz, 1H), 3.03 (m, 1H), 2.85 (d, 15.0 Hz, 1H), 2.69 and 2.61 (two d, 14.5 Hz, 4H), 1.60–2.20 (m, 17H), 1.55 and 1.51 (two d, 14.5 Hz, 2H), 1.46 (broad s, 1H), 1.38 (d, 14.5 Hz, 1H). For the peaks assignments, see Supporting Information. ^13^C-NMR (δ, CD_3_OD, 124.9 MHz): 176.47, 51.81, 51.76, 50.71, 32.61, 31.93, 30.85, 30.36, 28.65, 28.25, 28.08, 26.88, 25.64, 25.59, 20.13. LC/MS (CI, pos. scan): *m/z* = 277.2.

*7,7′-Endo-3′-[(3-{[2-(trimethylsilyl)ethoxy]carbonyl}-3-azabicyclo[3.3.1]non-7-yl)carbonyl]-3′-azabicyclo[3.3.1]nonane-7′-carboxylic acid* (**27**). Amino acid **3** (100 mg, 0.59 mmol) was suspended in THF (2 mL) and triethylamine (120 mg, 1.2 mmol) was added to the suspension under stirring. The mixture was then cooled to −20 °C and 2-(trimethylsilyl)ethylcarbonochloridate (214 mg, 1.2 mmol) was slowly added under stirring, keeping the mixture temperature not higher than −10 °C. After the addition, the mixture was stirred at −10 °C for 15 min and at ambient temperature for 30 min. The mixture was then again cooled in an ice-water bath, and a suspension of **3** (100 mg, 0.59 mmol) in a triethylamine (60 mg, 0.6 mmol) solution in THF (2 mL) was added to the stirred mixture in one portion. The resulting mixture was stirred overnight at ambient temperature. The volatile products were removed in vacuum, the residue was triturated with aqueous citric acid solution (5%). The crude product was extracted with MTBE (3 × 50 mL), the combined extracts were evaporated and the residue was purified by reverse-phase HPLC to afford **27** as colorless oil which crystallized on standing. Yield: 178.2 mg, 65%. m.p. 198 °C. ^1^H-NMR (δ, CDCl_3_, 500 MHz): 9.83 (br. s, 1H), 4.36 (br. s, 1H), 4.13 (br. s, 2H), 3.80–4.05 (br. m, 3H), 3.12 (br. s, 1H), 2.65–2.82 (br. m, 5H), 2.53 (br. s, 1H), 2.24 (br. m, 2H), 1.75–2.15 (br. m, 9H), 1.64 (br. s, 1H), 1.57 (br. s, 2H), 1.21 (br. s, 1H), 0.98 (br. m, 2H), −0.1 (br. m, 9H). ^13^C-NMR (δ, CDCl_3_, 124.9 MHz): (all the signals are broadened) 117.72, 117.27, 157.53, 63.64, 50.73, 47.37, 36.83, 33.33, 33.12, 31.31, 30.02, 29.30, 29.05, 28.53, 27.26, 27.07, 26.94, 26.78, 25.81, 25.52, 25.32, -1.47. LC/MS (CI, neg. scan): m/z = 463.2, 454.2, 465.2; (CI, pos. scan): m/z = 466.2, 467.2, 468.2. Anal. Calc. for C_24_H_40_N_2_O_5_Si: C, 62.03; H, 8.68; N, 6.03. Found: C, 62.08; H, 8.64; N, 6.00.

*7,7′-Endo-3′-(3-azabicyclo[3.3.1]non-7-ylcarbonyl)-3′-azabicyclo[3.3.1]nonane-7′-carboxylic acid hydrochloride* (**2**·HCl). Compound **27** (50 mg, 0.11 mmol) was dissolved in a mixture of dry diethyl ether and dichloromethane (1:1 *v/v*, 1 mL) and a solution of HCl in dry dioxane (~10%, 0.5 mL) was added. The mixture was left standing at ambient temperature for 8 h, the formed white crystals of the product (**2**·HCl) were filtered, washed thoroughly with dry diethyl ether on the filter, dried in high vacuum (0.05 mm Hg) for 6 h at 25 °C). Yield: 18 mg (46%). m.p. 198 °C (with decomposition). ^1^H-NMR (δ, D_2_O, 500 MHz): 4.06 (d, 13.5 Hz, 1H), 3.85 (d, 13.5 Hz, 1H), 3.10–3.33 (m, 3H), 2.90–3.15 (m, 3H), 2.74 (dd, 13.5 and 2.0 Hz, 1H), 2.57 (h, 2.0 Hz, 1H), 1.85-2.20 (overlapped m, 11H), 1.73 (dd, 13.5 and 3.0 Hz, 1H), 1.50-1.70 (m, 4H), 1.34 (q, 3.0 Hz, 1H). ^13^C-NMR (δ, D_2_O, 124.9 MHz): 180.58, 179.33, 50.75, 49.01, 48.32, 47.98, 35.51, 29.83, 29.26, 29.10, 28.72, 27.11, 26.36, 26.30, 26.28, 26.09, 23.74, 23.39. Anal. Calc. for C_18_H_29_ClN_2_O_3_: C, 60.58; H, 8.19; N, 7.85. Found: C, 60.54; H, 8.22; N, 7.83.

#### 4.2.1. Kinetic Measurements for Hydrolysis Reaction of Compound 2 in D_2_O

Hydrolysis was carried out at 23 °C in NMR sample tubes, monitored by ^1^H-NMR spectra (recorded by single scans). Six buffer solutions were prepared in D_2_O: acetate buffers (pD 3.81 and 4.85), phosphate buffers (pD 6.68 and 7.95), and carbonate buffers (pD 9.45 and 10.68); the pD values were measured at 23 °C immediately before the measurements. The buffer concentrations were 250 mM in each case, the ionic strengths were adjusted with NaCl to 1 M. The compound **2**·HCl was weighed in an NMR sample tube (5–7 mg), and the measurements started by addition of the corresponding buffer solution to the compound (in the amount to achieve the compound concentration 25 mM). The content of the tube was vigorously shaken, starting immediately the recording of the time. The first ^1^H-NMR spectrum was measured as quickly after the buffer addition as practical (~2 min), then the spectra were measured at intervals, depending on the reaction progress. The spectra were run as single scans, in order to avoid integral-value distortions due to relaxation effects. The unambiguously assigned non-overlapping peaks of the starting compound and its hydrolysis product were carefully integrated. The complete data sets (Tables S1–S12) were used to build the kinetic curves and calculate *k_obs_* and the half-lives for each compound (see the Supporting Information).

#### 4.2.2. Estimation of the Kinetic Isotope Effect by Comparison of the Hydrolysis Rates of 2·HCl in D_2_O and H_2_O

Two reactions were set up in two NMR sample tubes simultaneously by dissolving 4.2 mg of the **2**·HCl in each tube in 0.5 cm^3^ D_2_O or H_2_O. The ^1^H-NMR spectra of the samples were run at intervals (as single scans, in order to avoid integral-value distortions due to relaxation effects). For the spectral measurements in H_2_O, excitation sculpting technique [55] was used for the water signal suppression. The unambiguously assigned non-overlapping peaks of the starting compound and its hydrolysis product were carefully integrated. Representative spectra can be seen in Figures S13 and S14.

#### 4.2.3. X-ray Data for Compound 13

Crystal Data for C_19_H_29_NO_3_ (*M* = 319.43 g/mol): monoclinic, space group *P*2_1_/*c* (no. 14), *a* = 6.9409(2) Å, *b* = 10.8409(3) Å, *c* = 22.2437(6) Å, β = 93.2098(12)°, *V* = 1671.11(8) Å^3^, *Z* = 4, *T* = 180(2) K, μ(CuKα) = 0.673 mm^-1^, *D*_calc_ = 1.270 g/cm^3^. The diffraction pattern was indexed as two domains with 19230 measured reflections (domain 1 only), 19298 reflections (domain 2 only) and 3794 reflections (overlapped) (7.96° ≤ 2 ≤ 133.37°), *R*_int_ = 0.0399. 2954 unique reflections were used in all calculations. The final *R*1 was 0.0599 (*I* > 2σ(*I*)) and *wR*2 was 0.1593 (all data).

CCDC 1884473 contains the supplementary crystallographic data for this paper. These data can be obtained free of charge via https://www.ccdc.cam.ac.uk/structures (or from the CCDC, 12 Union Road, Cambridge CB2 1EZ, UK; Fax: +44 1223 336033; E-mail: deposit@ccdc.cam.ac.uk).

## Figures and Tables

**Table 1:** Kinetic parameters for the hydrolysis of **2**·HCl in acidic D_2_O buffers at different pD values, 23 °C.

pD Value	Half-life of 2·HCl, min	*k_obs_*, min^−1^	*Log(k_obs_)*
3.81	18,138	3.821 × 10^−5^	−4.418
4.85	4668	1.485 × 10^−4^	−3.828
6.68	3748	1.849 × 10^−4^	−3.733

**Table 2:** Kinetic parameters for the hydrolysis of **2**·HCl in alkaline D_2_O buffers at different pD values, 23 °C.

pD Value	Half-life of 2·HCl, min	*k_obs_*, min^−1^	*Log(k_obs_)*
7.95	18,802	3.686 × 10^−5^	−4.433
9.45	91,125	7.605 × 10^−6^	−5.119
10.68	126,114	5.496 × 10^−6^	−5.260

## Supplementary Materials

The following are available online. Figures S1–S36, NMR spectra and chromato-mass traces for the compounds described in the main text; Tables S1–S12, Figures S37–S48, kinetic data on the hydrolysis of **2**·HCl in different buffers; Figures S49 and S50, Representative ^1^H-NMR data set used for estimation of the kinetic isotope effect on the **2**·HCl hydrolysis reaction; Figure S51; The CIF file for compound **13**.
