# Supplementary material for: Fast Amide Bond Cleavage Assisted by a Secondary Amino and a Carboxyl Group—A Model for yet Unknown Peptidases?
Source: Molecules. 2019 Feb 5;24(3):572. doi: 10.3390/molecules24030572 (PMC6384577; doi:10.3390/molecules24030572)
Supplement: Supplementary file 1 [file molecules-24-00572-s001.zip › Supporting_Information.pdf]

SUPPORTING INFORMATION

Fast Amide Bond Cleavage Assisted by a Secondary Amino and a Carboxyl Group – a Model for yet Unknown Peptidases?

Igor V. Komarov[1]*, Aleksandr Yu. Ishchenko[2], Aleksandr Hovtvianitsa[2], Viacheslav Stepanenko[2], Serhii Kharchenko[2], John E. Davies[3], Andrew D. Bond[3], and Anthony J. Kirby[3,*]

[1] Taras Shevchenko National University of Kyiv, Institute of High Technologies, Vul. Volodymyrska 64/13, 01601 Kyiv, Ukraine; ik214@yahoo.com

[2] Enamine Ltd., Vul. Chervonotkatska 78, 02094 Kyiv, Ukraine; igor.komarov@enamine.net

[3] University Chemical Laboratory, University of Cambridge, Lensfield Road, CB2 1EW Cambridge, UK; ajk1@cam.ac.uk

* Correspondence: ajk1@cam.ac.uk; ik214@yahoo.com

Table of content

NMR spectra and chromato-mass traces for the compounds described in the main text.............. S2-S37

Kinetic data on the hydrolysis of 2·HCl in different buffers...S38-S49

Representative ^{1}H-NMR data set used for estimation of the kinetic isotope effect of the 2·HCl hydrolysis reaction...S50-S51

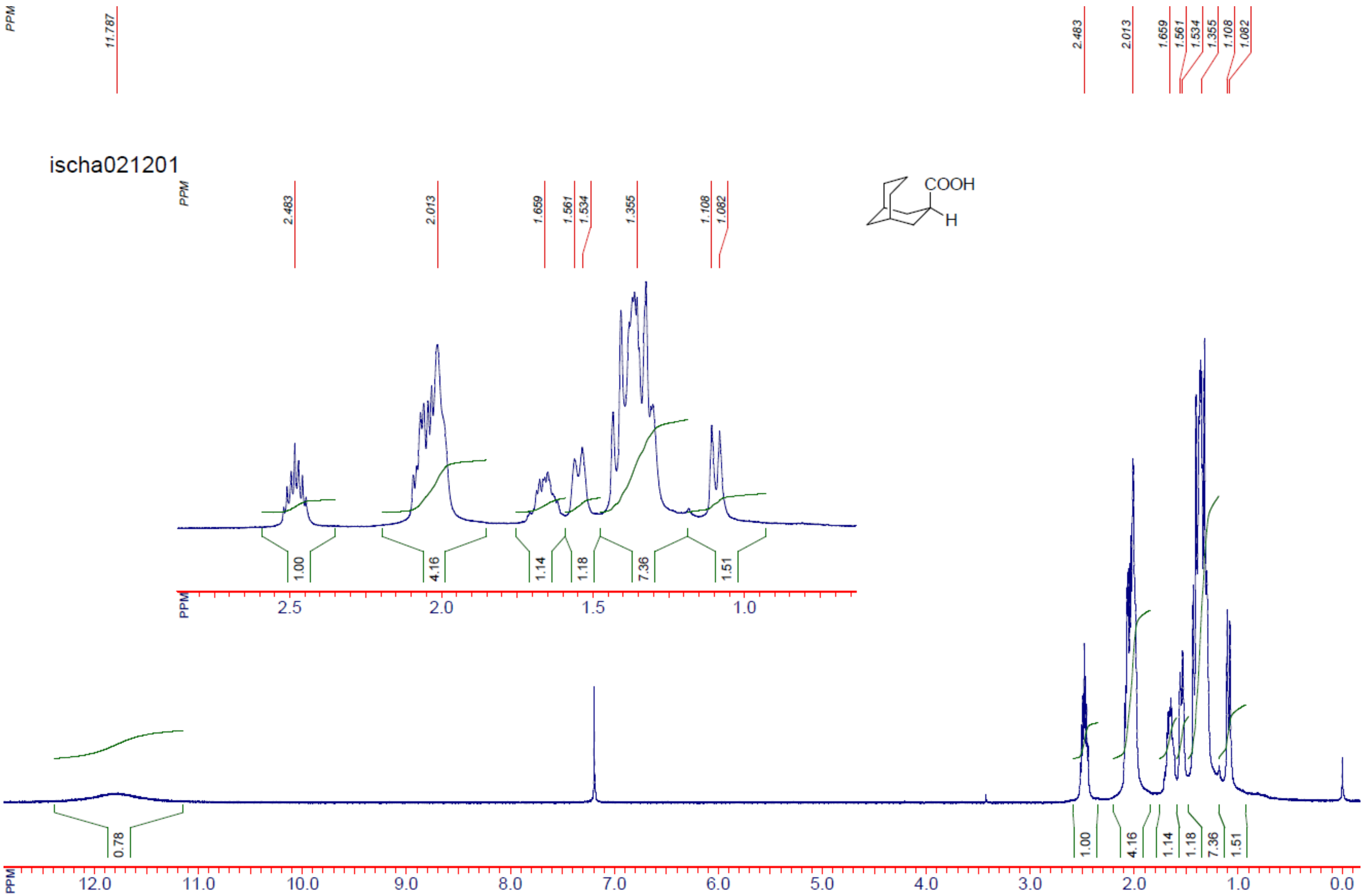

**File name: ischa021201**	Operator: root	SF: 499.9500 MHz	NSC: 1	PW: 0.00 usec, RG: 33	SI: 32768
Date: 02-Dec-2014	Solvent: cdcl3	SW: 8993 Hz	TE: 0 K	AQ: 1.82 sec, RD: 0.00 sec	Parameter file, XWIN-NMRVersion 3.5

Figure S1. ^{1}H-NMR spectrum of compound **20**.

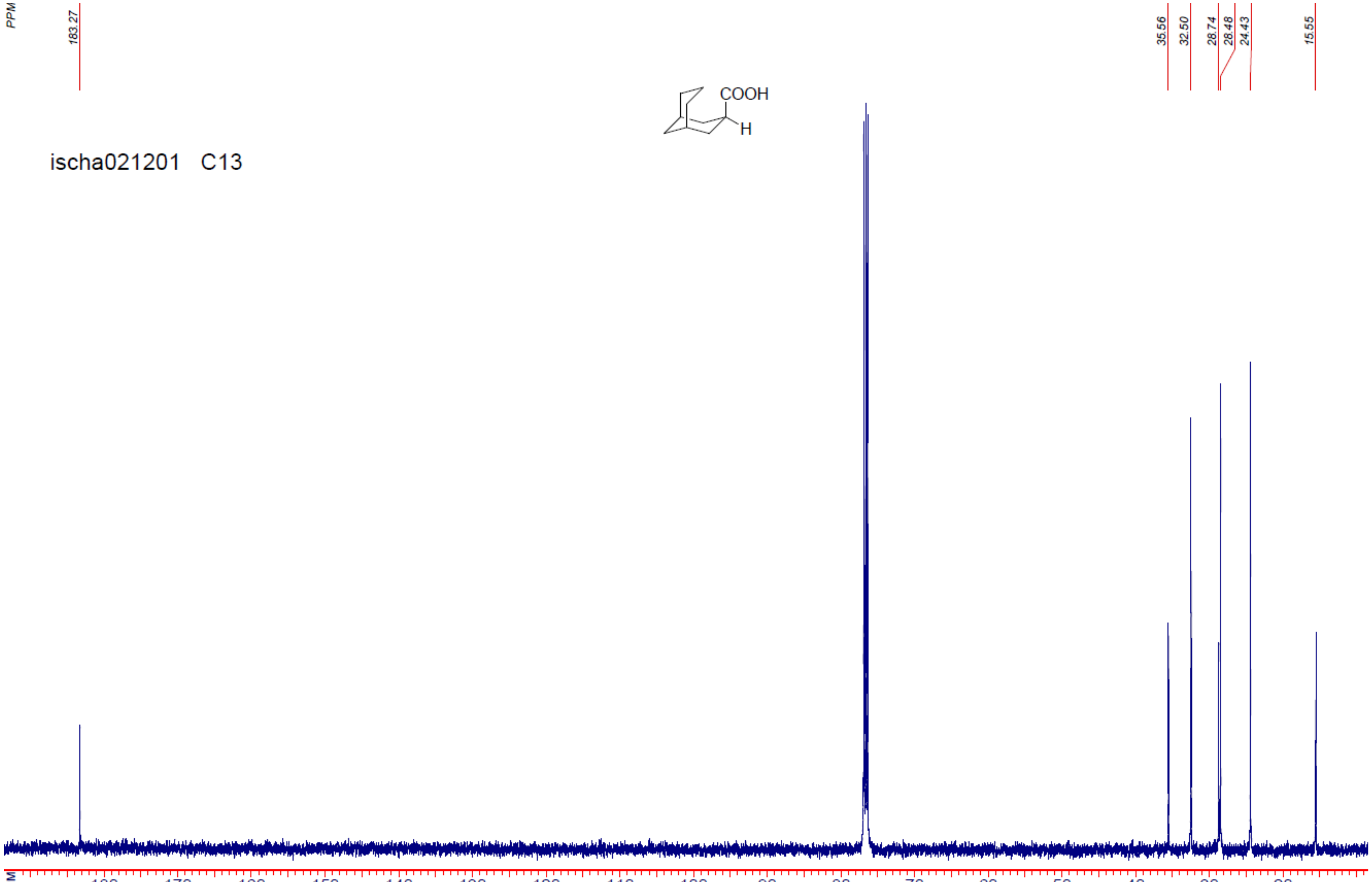

**File name: ischa021201 C13**	Operator: root	SF: 125.7126 MHz	NSC: 366	PW: 0.00 usec, RG: 51200	SI: 65536
Date: 02-Dec-2014	Solvent: CDCl3	SW: 32680 Hz	TE: 0 K	AQ: 1.57 sec, RD: 0.00 sec	Parameter file, XWIN-NMRVersion 3.5

Figure S2. ^{13}C-NMR spectrum of compound **20**.

```
Data Path : C:\msdchem\1\data\11_12\
Data File : CLN22363.D
Acq On    : 12 Nov 2014  12:54
Operator  :
Sample    : CLN22363
Misc      : CH3OH
ALS Vial  : 42   Sample Multiplier: 1

Search Libraries:   C:\Database\EMPTY.L              Minimum Quality:   0

Unknown Spectrum:   Apex
Integration Events: ChemStation Integrator - autoint1.e
```

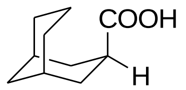

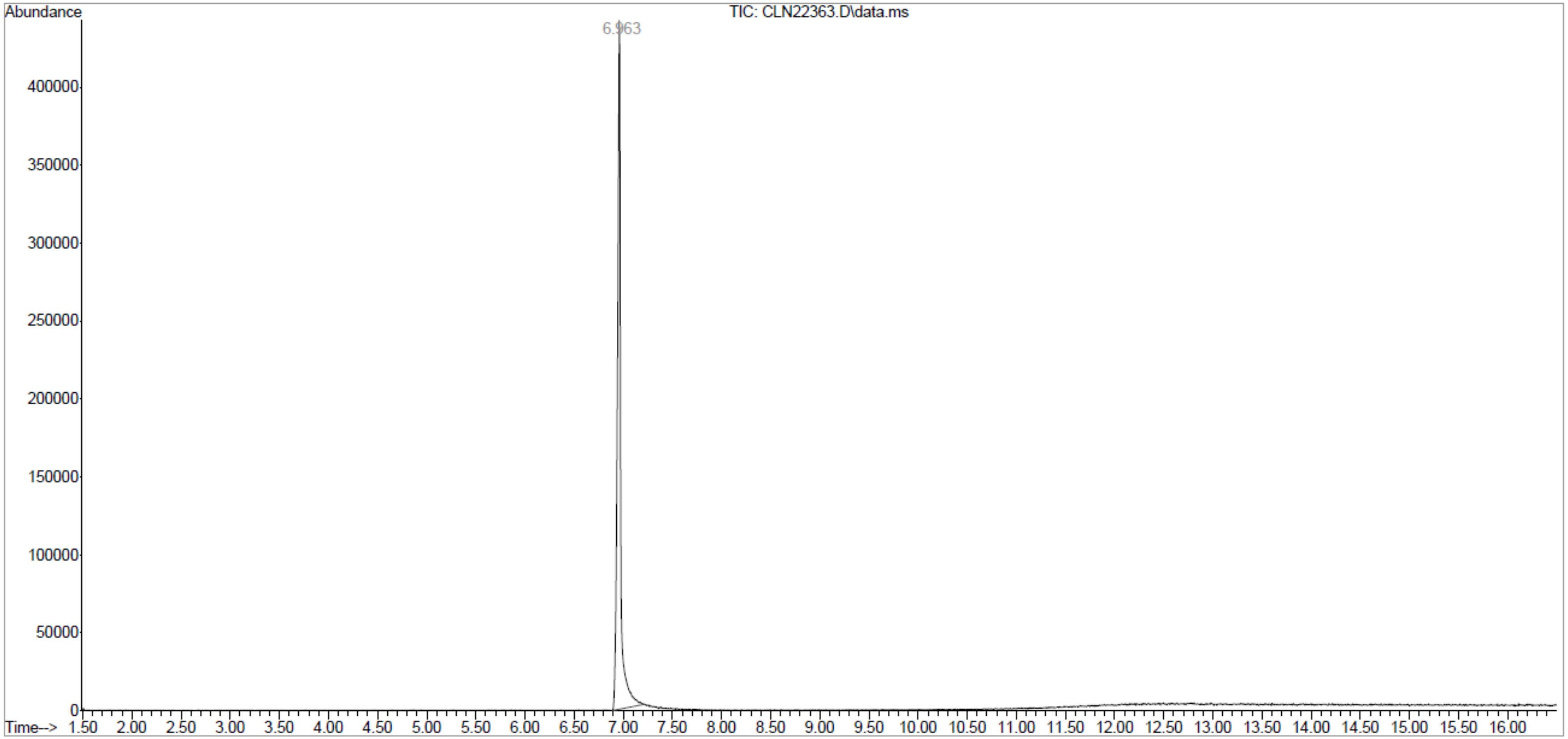

Figure S3. GC-MS trace of compound **20** (continued overleaf).

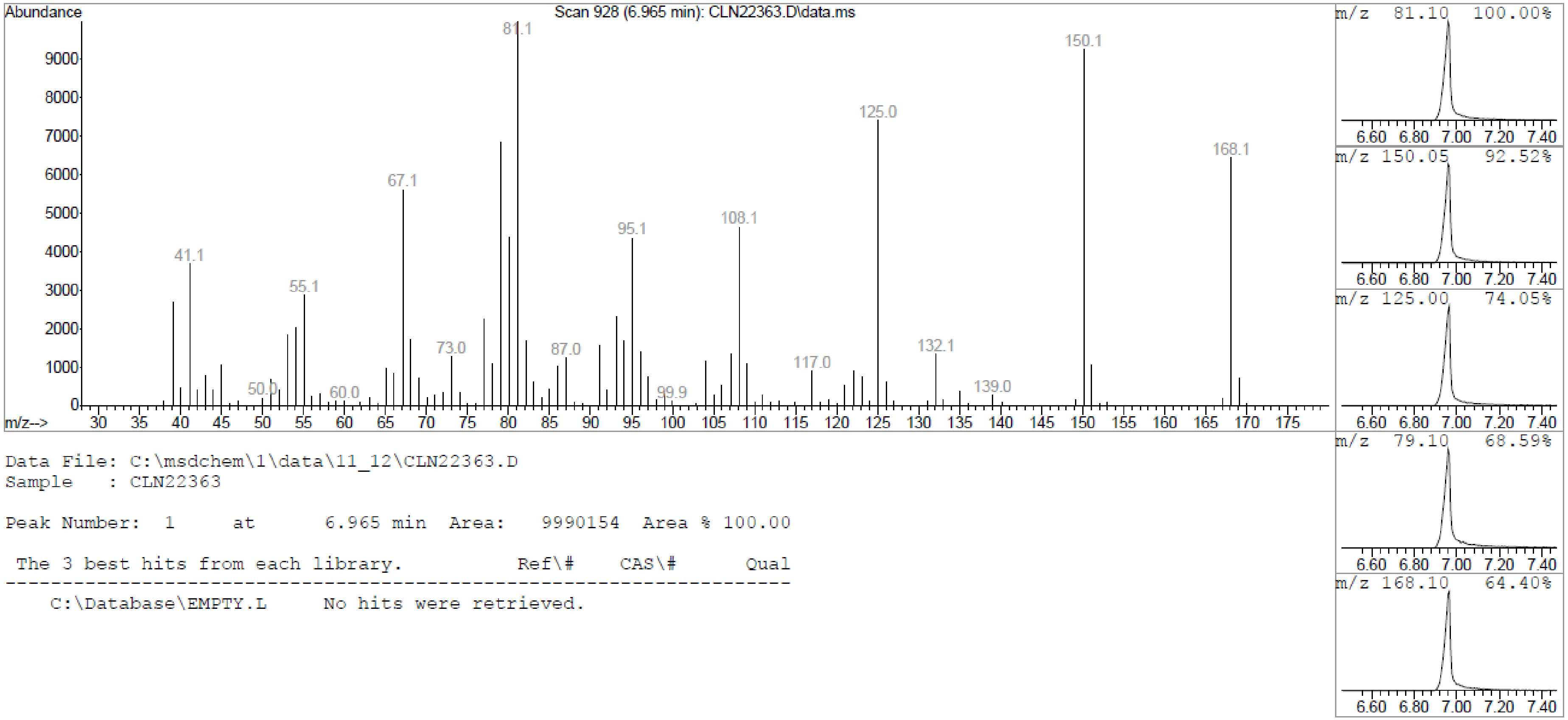

Figure S4. GC-MS trace of compound **20**.

Error: Peaks not found!

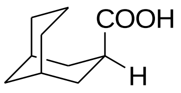

Mol Wt 0
Exact Mass

Error: Peaks not found!

RT 1.269

RT 1.269

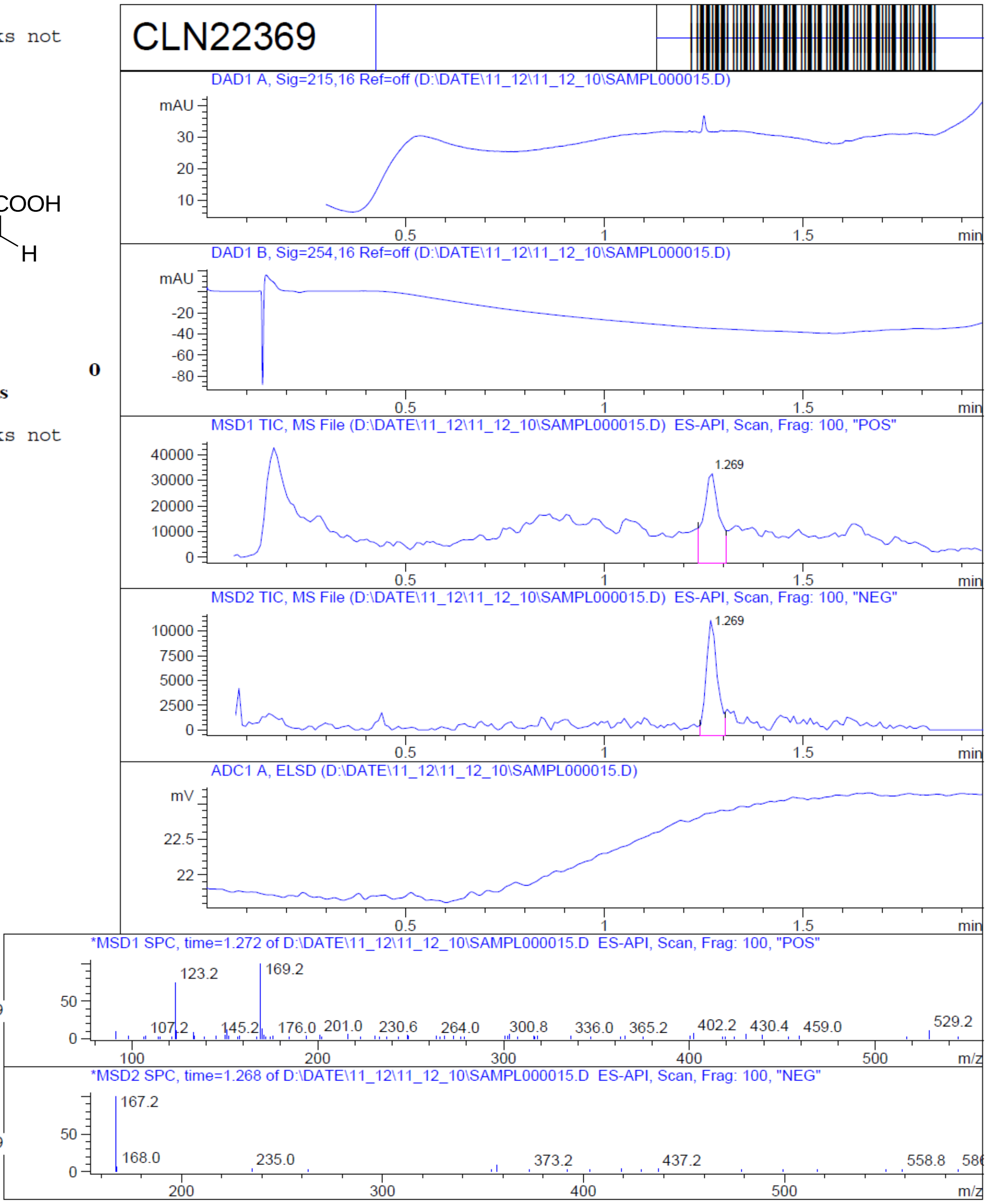

Inj.Date 11/12/2014 L P2-A-08 -5- Acq. Method C:\Chem32\-> ->

Figure S5. LC-MS trace of compound **20**.

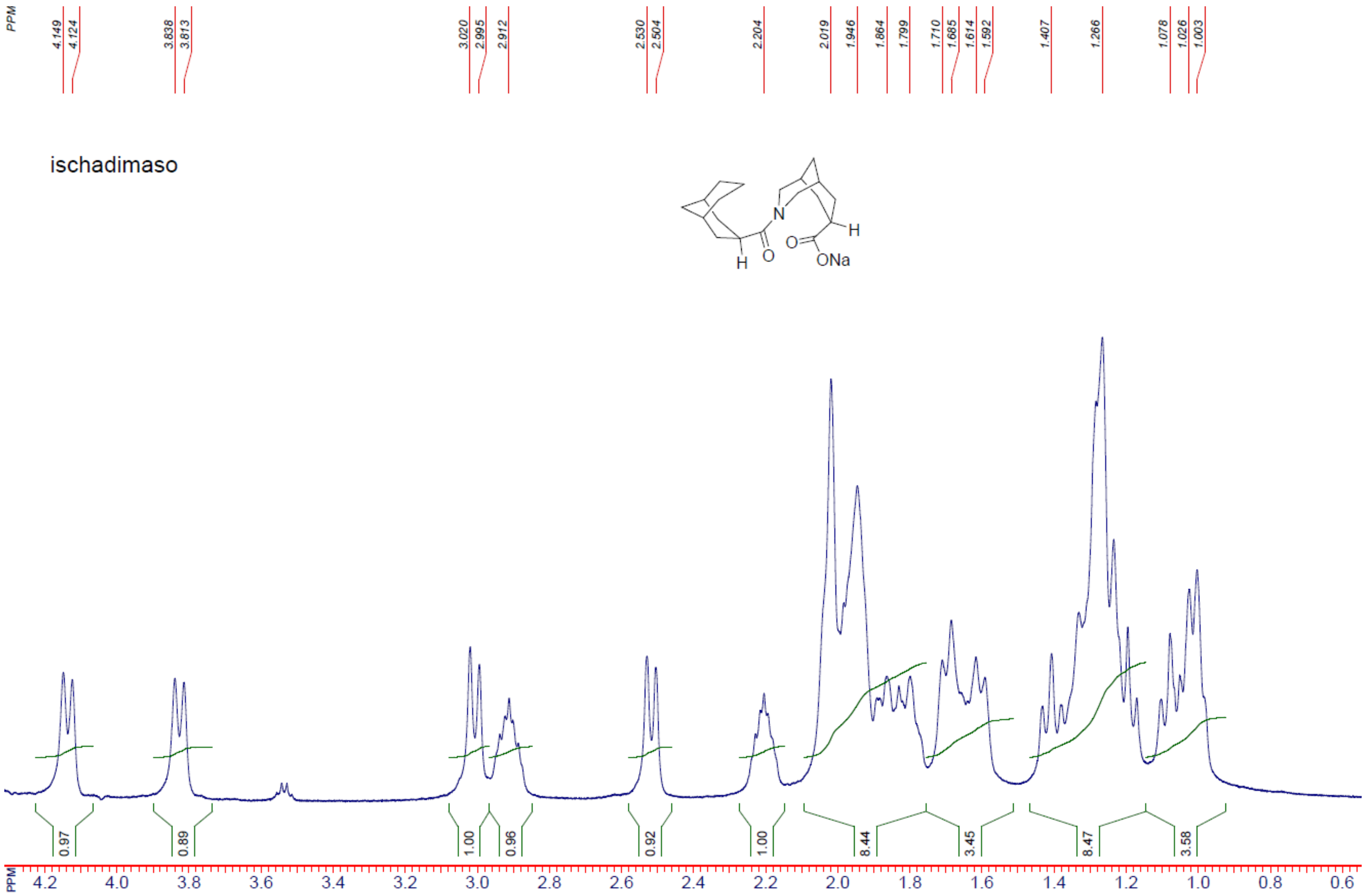

**File name: ischadimaso**	Operator: root	SF: 499.9500 MHz	NSC: 1	PW: 0.00 usec, RG: 77	SI: 32768
Date: 15-Jan-2015	Solvent: D2O	SW: 8993 Hz	TE: 0 K	AQ: 1.82 sec, RD: 0.00 sec	Parameter file, XWIN-NMRVersion 3.5

Figure S6. ^{1}H-NMR spectrum of sodium salt of compound **13**.

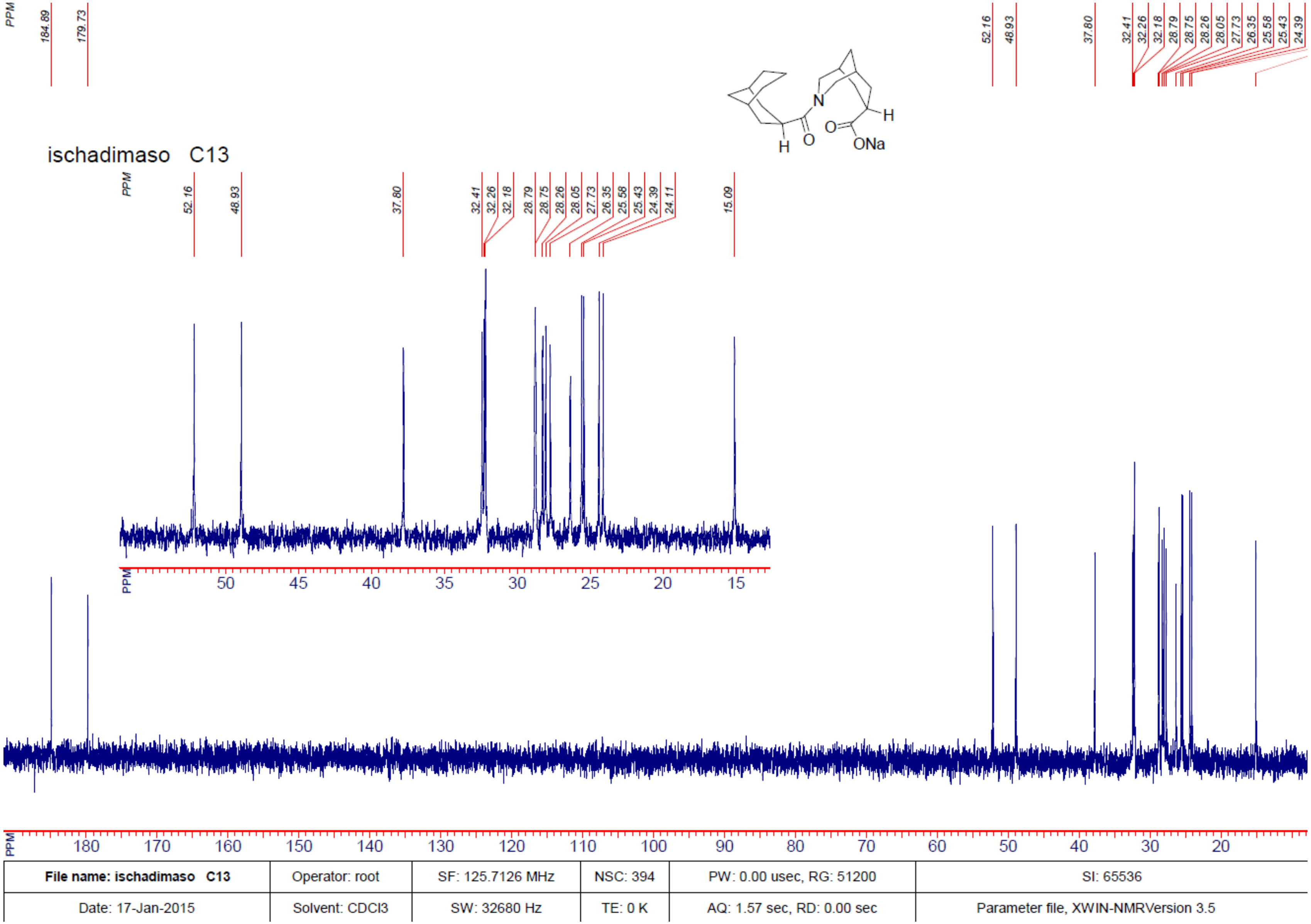

Figure S7. ^{13}C-NMR spectrum of sodium salt of compound **13**.

MaxPeak: 91.08%
Ret_Time: 1.384 min

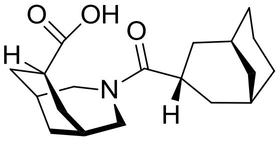

Mol Wt 0
Exact Mass

#	Time	Area%
1	1.180	8.92
2	1.384	91.08

RT 1.397

RT 1.212

RT 1.396

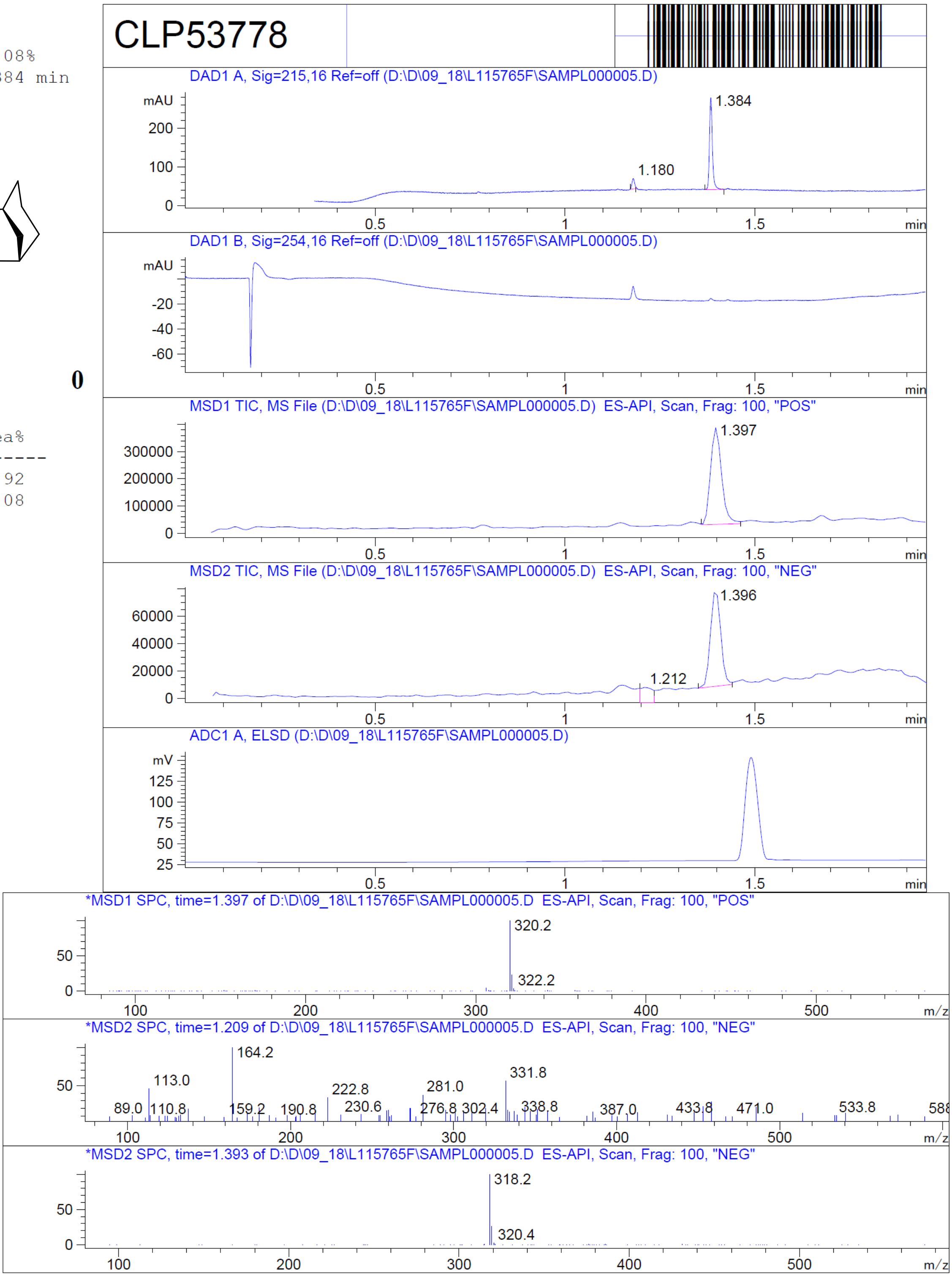

Inj.Date 9/18/2018 N P2-A-04 -5- Acq. Method C:\CHEM32\-> ->

Figure S8. LC-MS trace of compound **13**.

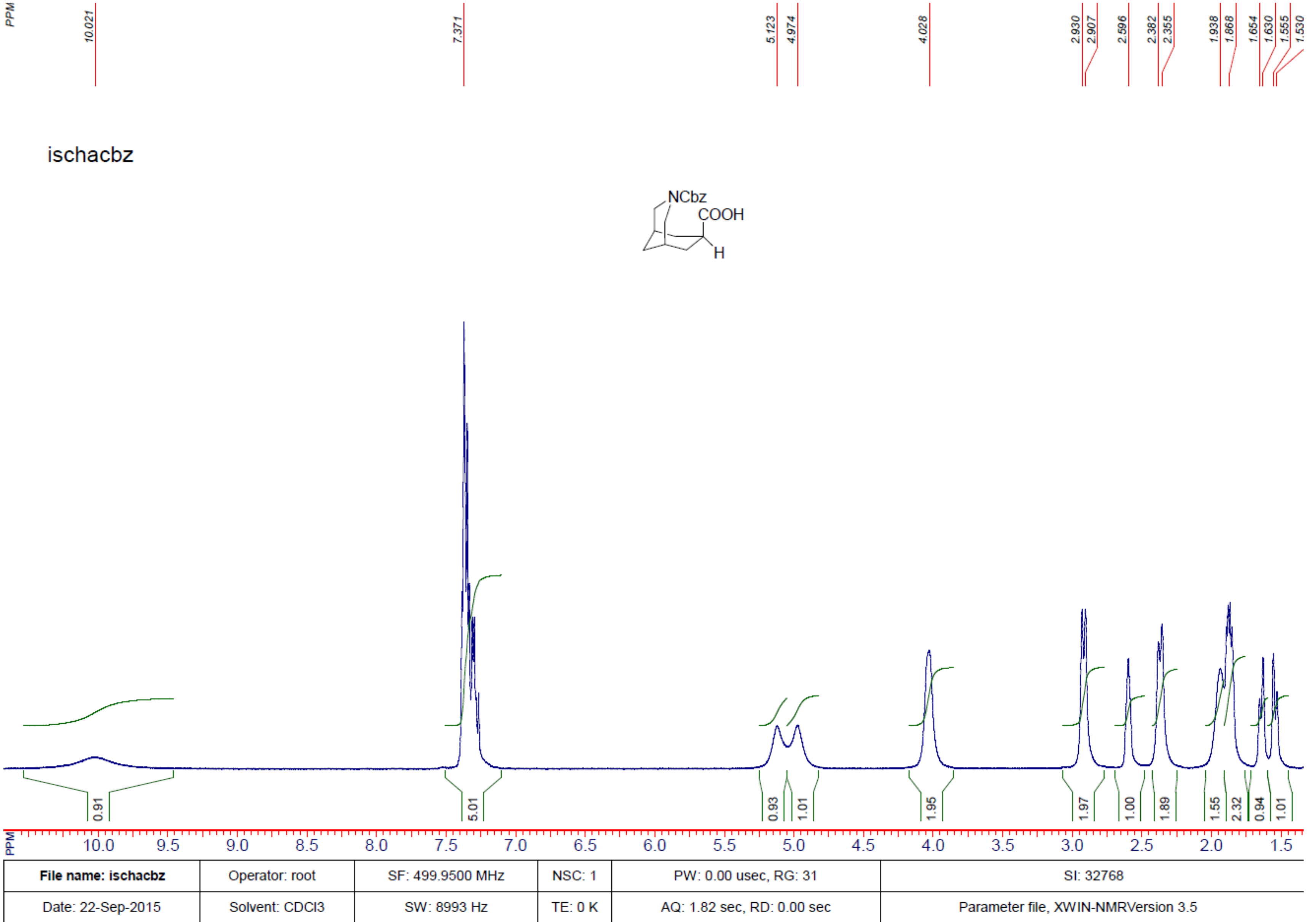

Figure S9. ^{1}H-NMR spectrum of compound **21**.

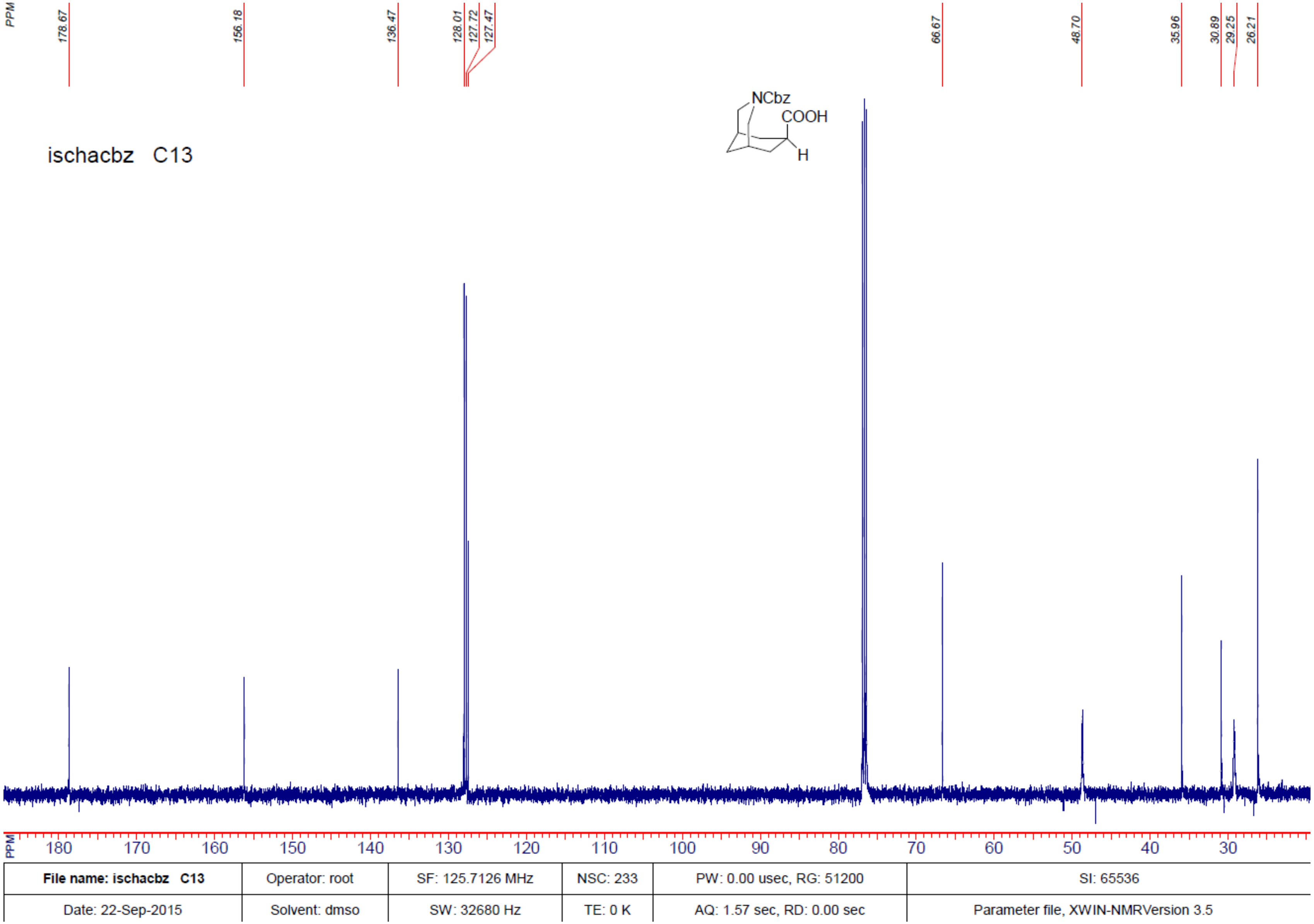

Figure S10. ^{13}C-NMR spectrum of compound **21**.

MaxPeak: 100.00%
Ret_Time: 1.223 min

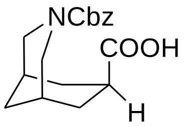

Mol Wt 0
Exact Mass

#	Time	Area%
1	1.223	100.00

RT 1.252

RT 1.253

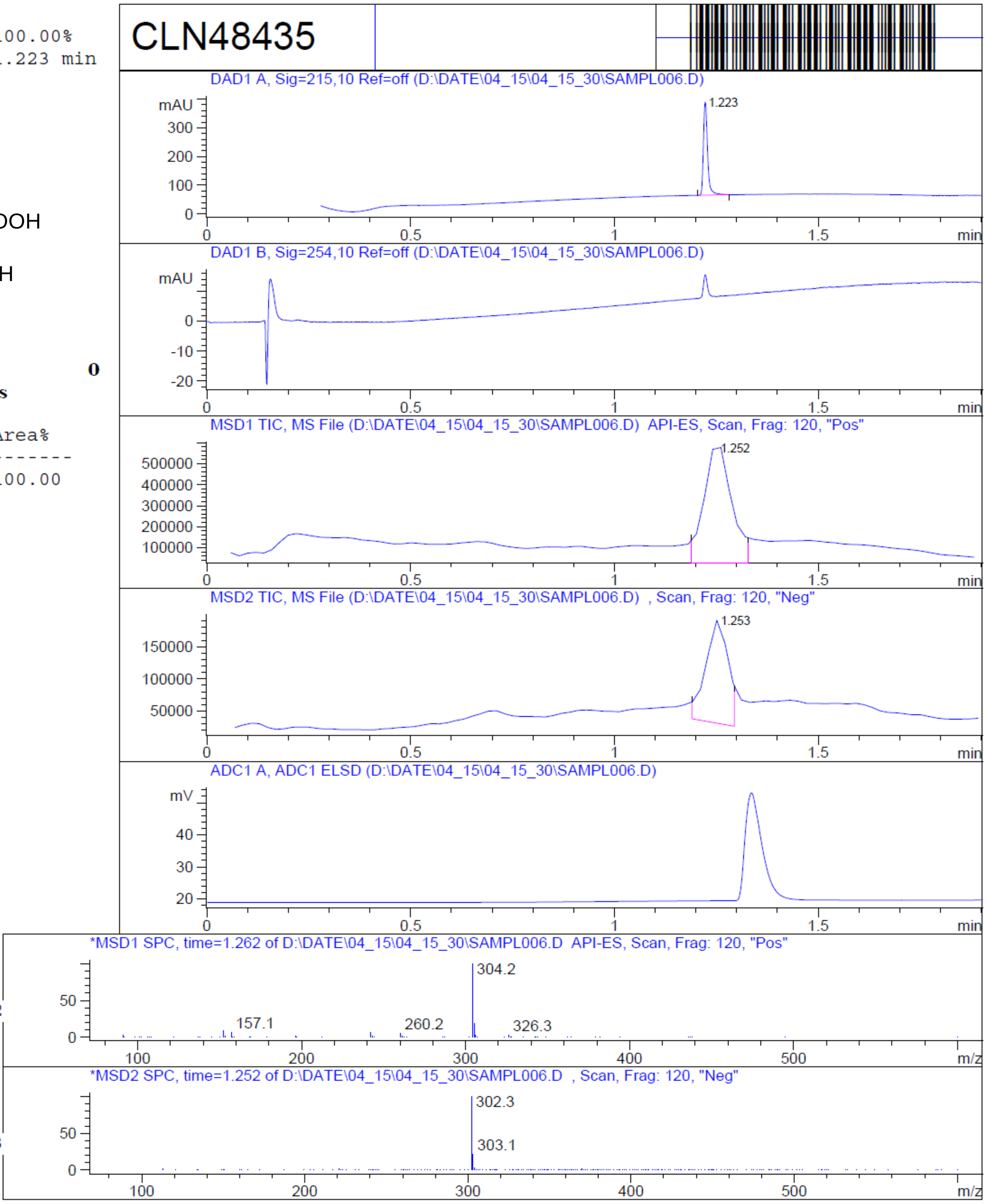

Figure S11. LC-MS trace of compound **21**.

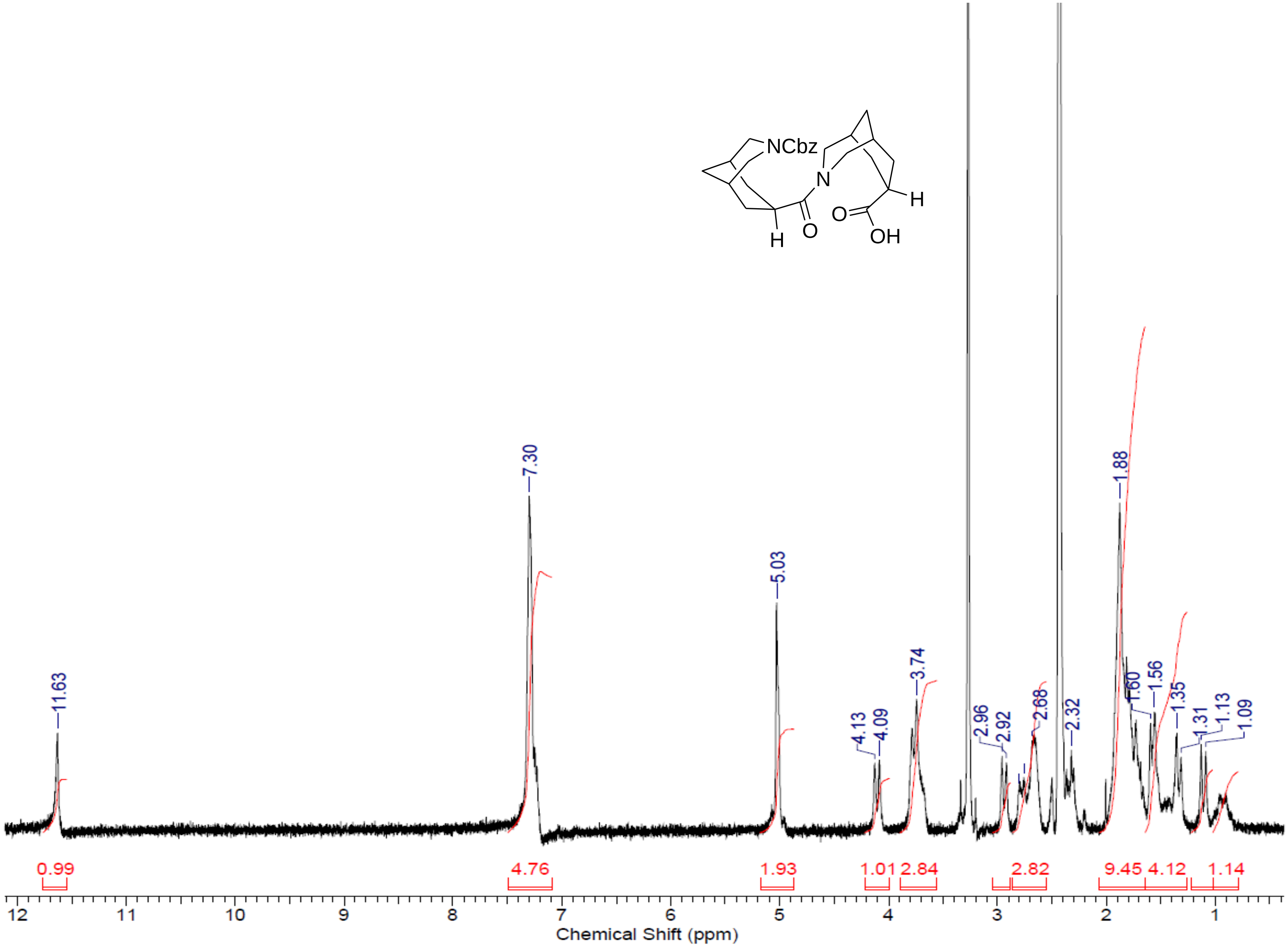

Figure S12. ^{1}H-NMR spectrum of compound **22**.

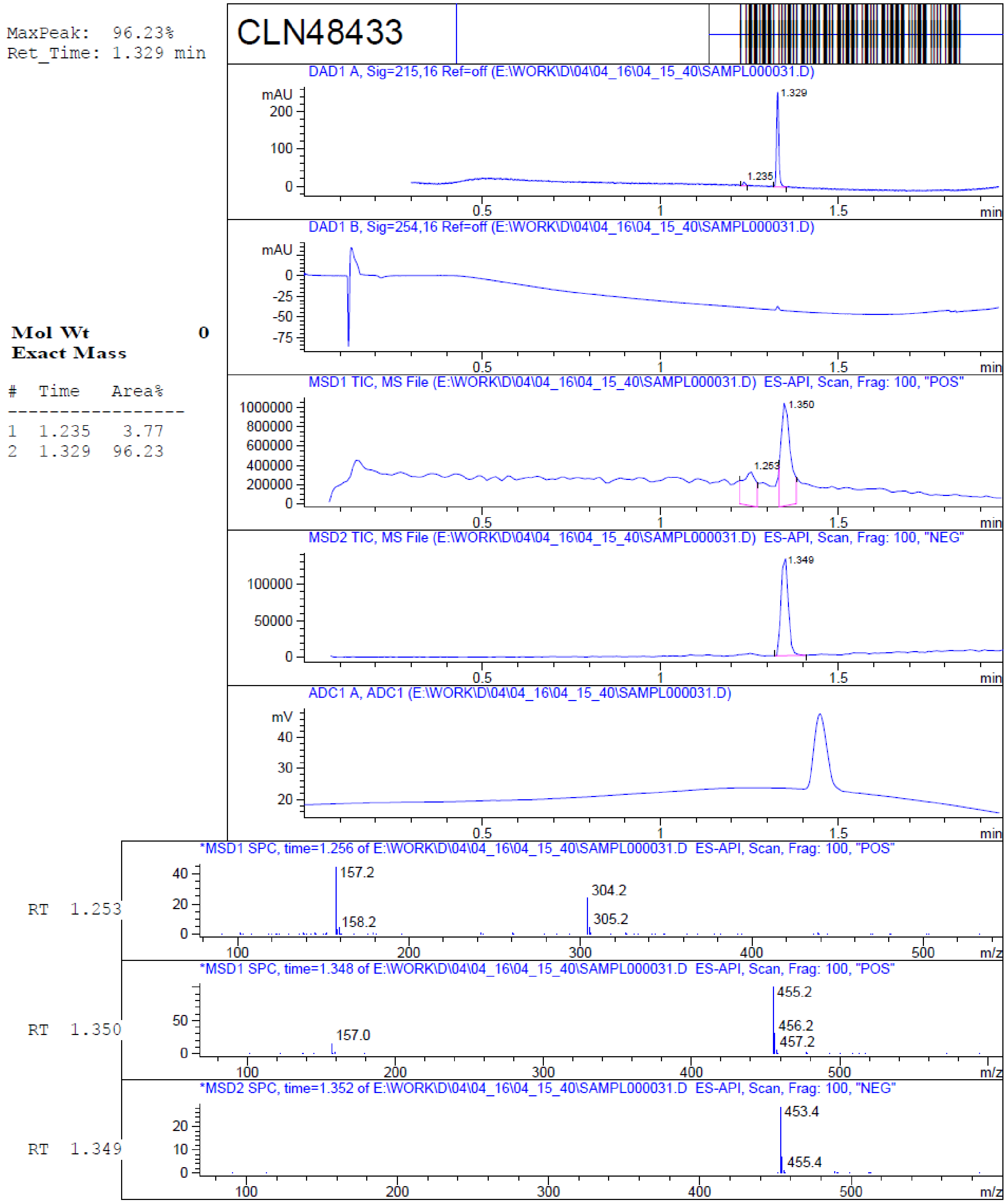

Figure S13. LC-MS trace of compound **22**.

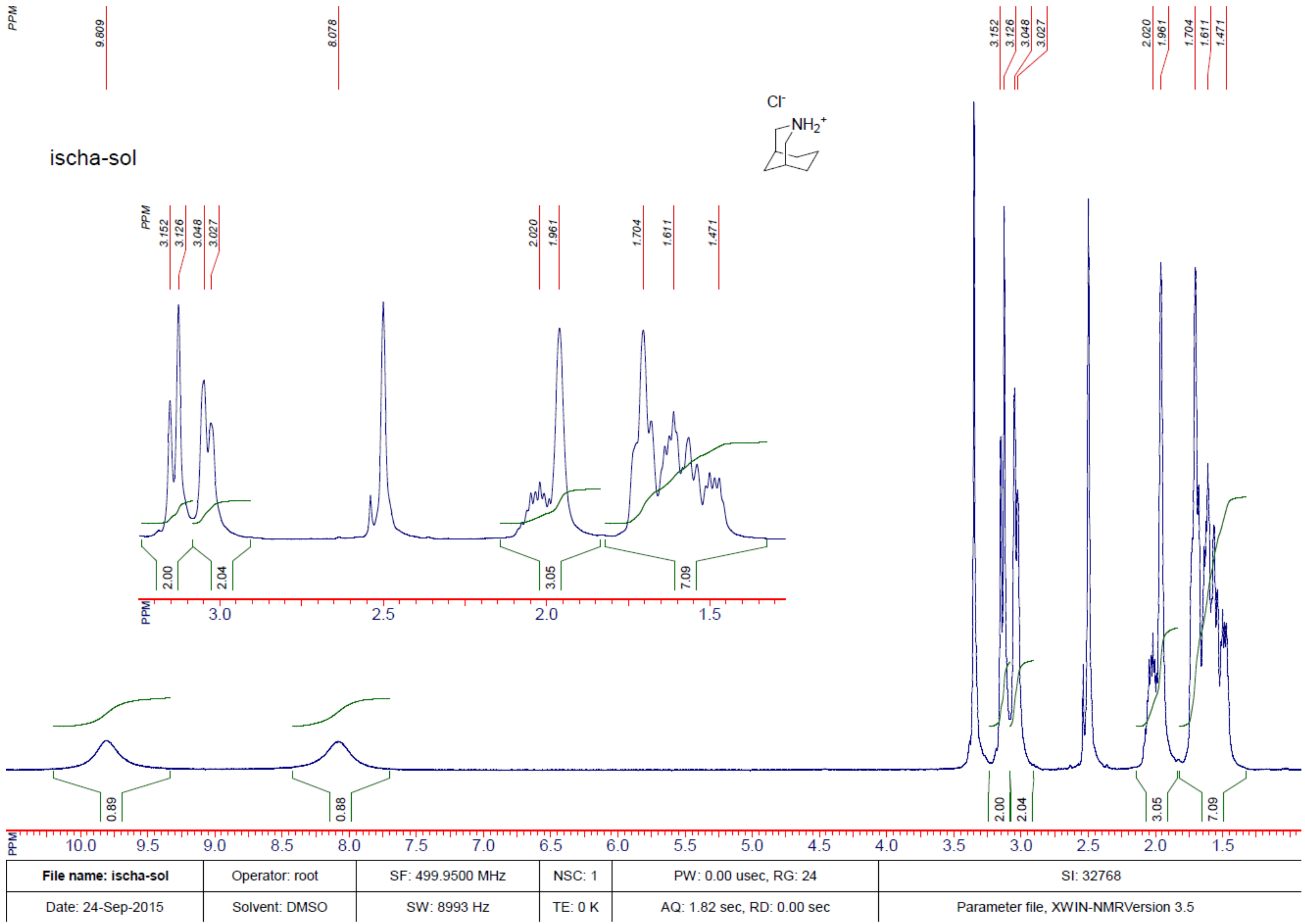

Figure S14. ^{1}H-NMR spectrum of compound **25** (hydrochloride).

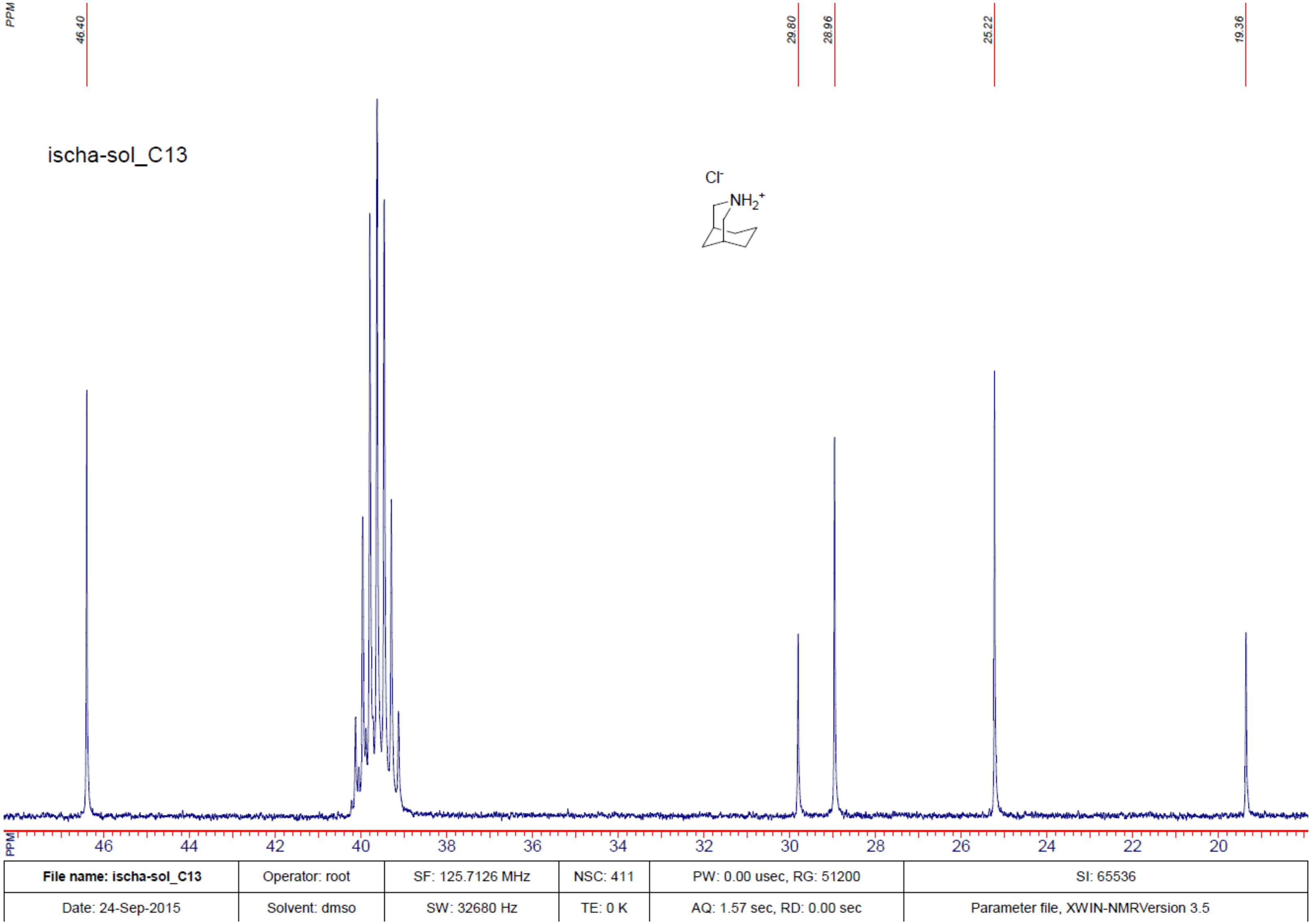

Figure S15. ^{13}C-NMR spectrum of compound **25** (hydrochloride).

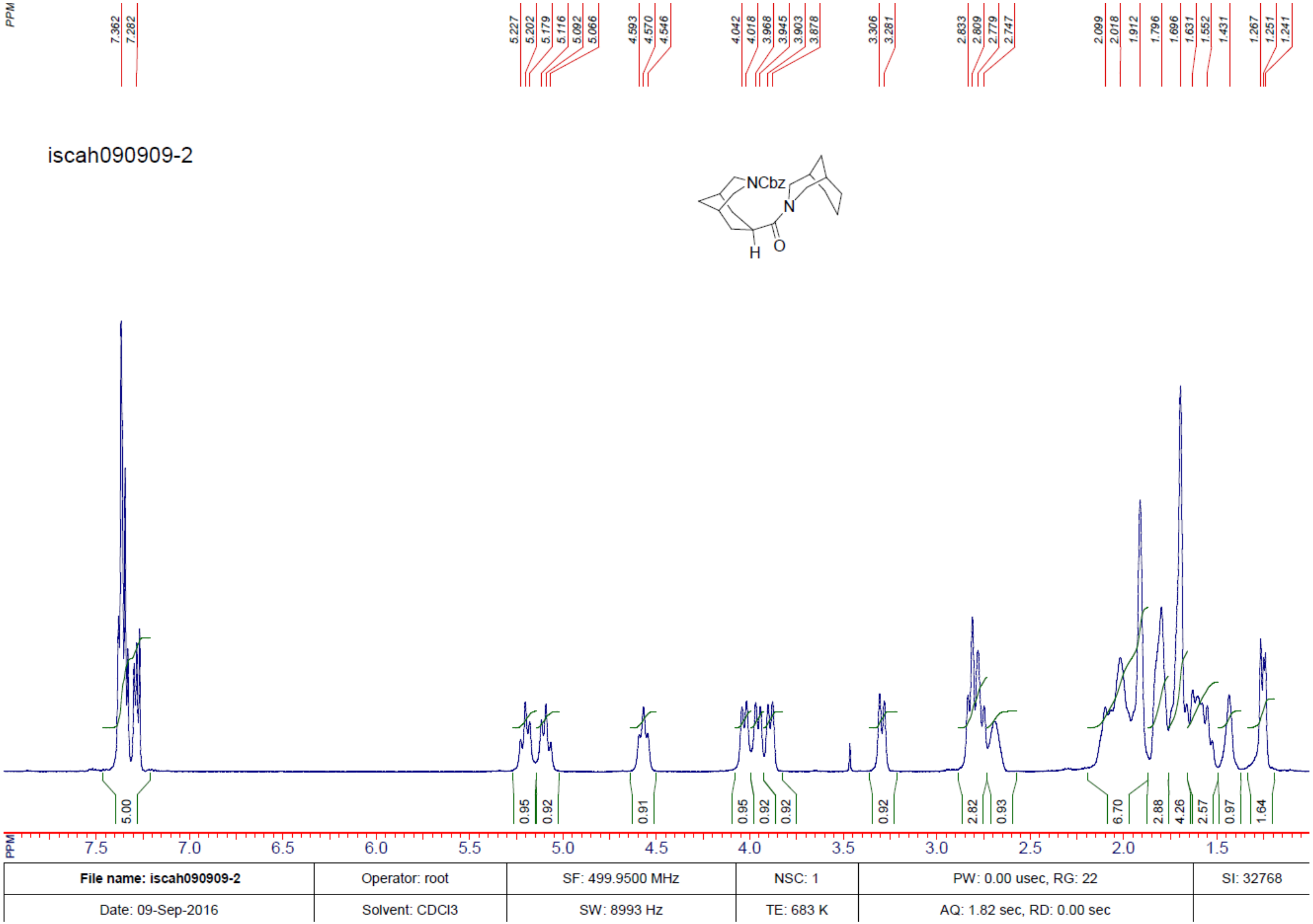

Figure S16. ^{1}H-NMR spectrum of compound **26**.

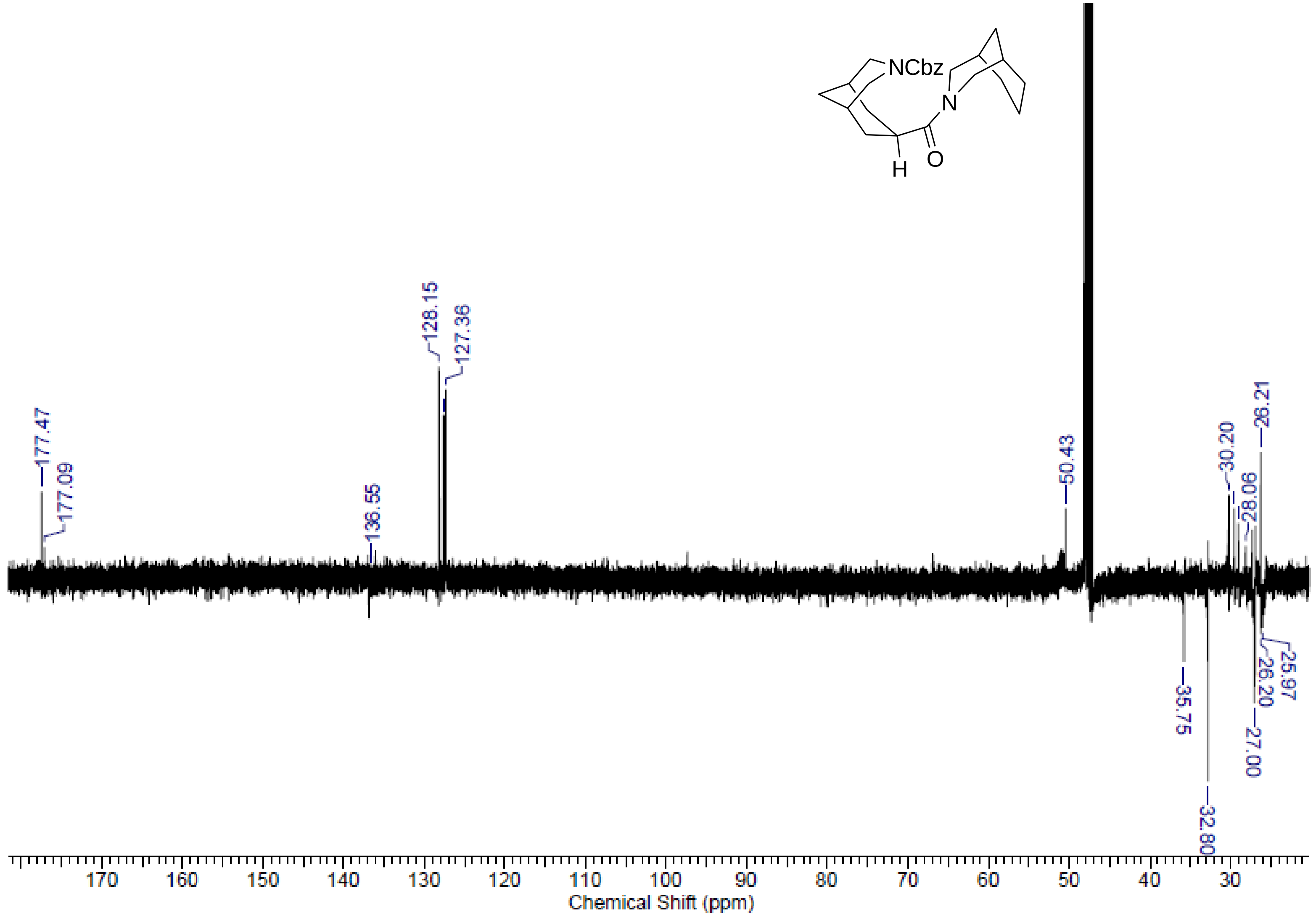

Figure S17. ^{13}C-NMR spectrum (INEPT) of compound **26**.

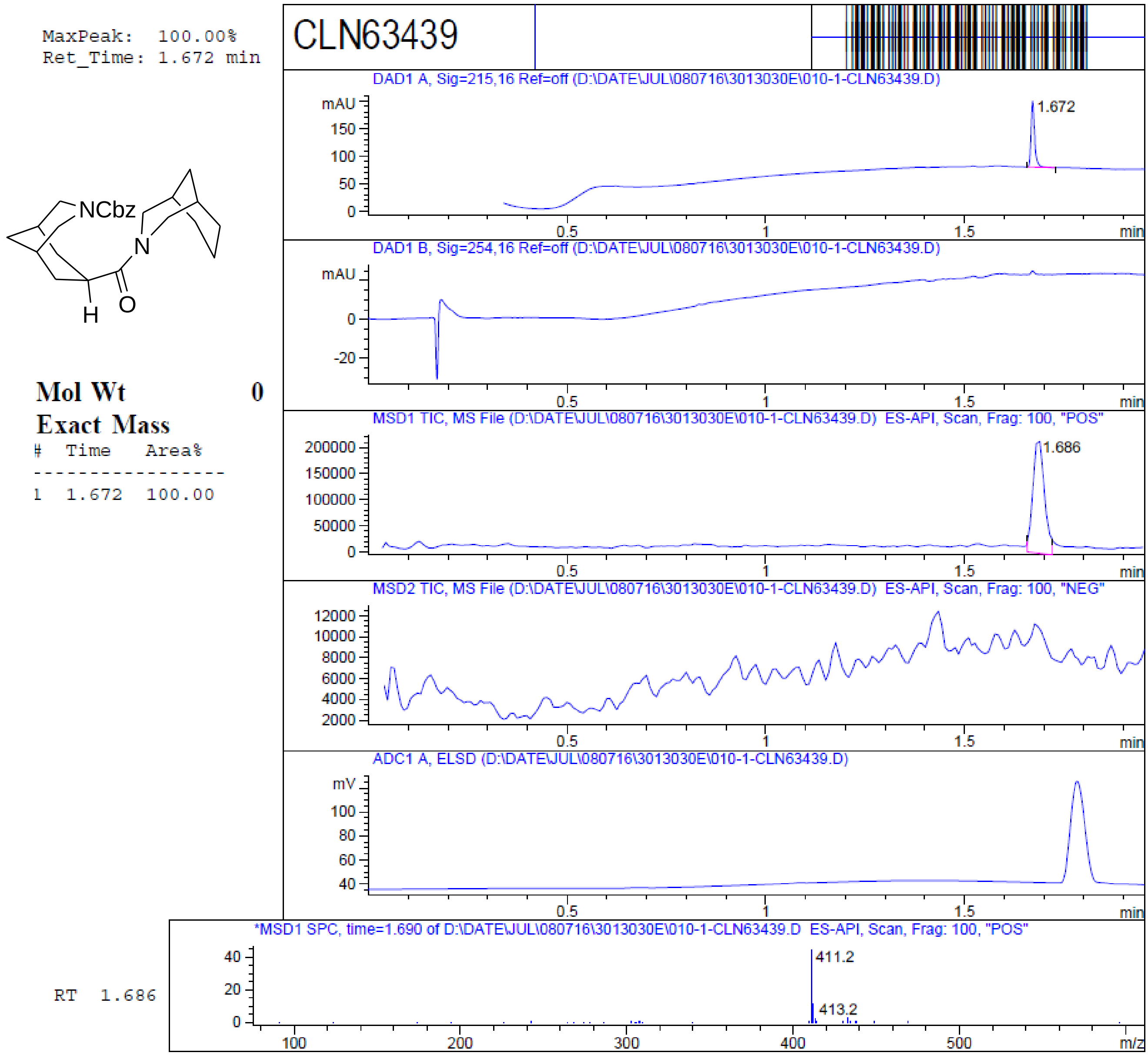

Figure S18. LC-MS trace of compound **26**.

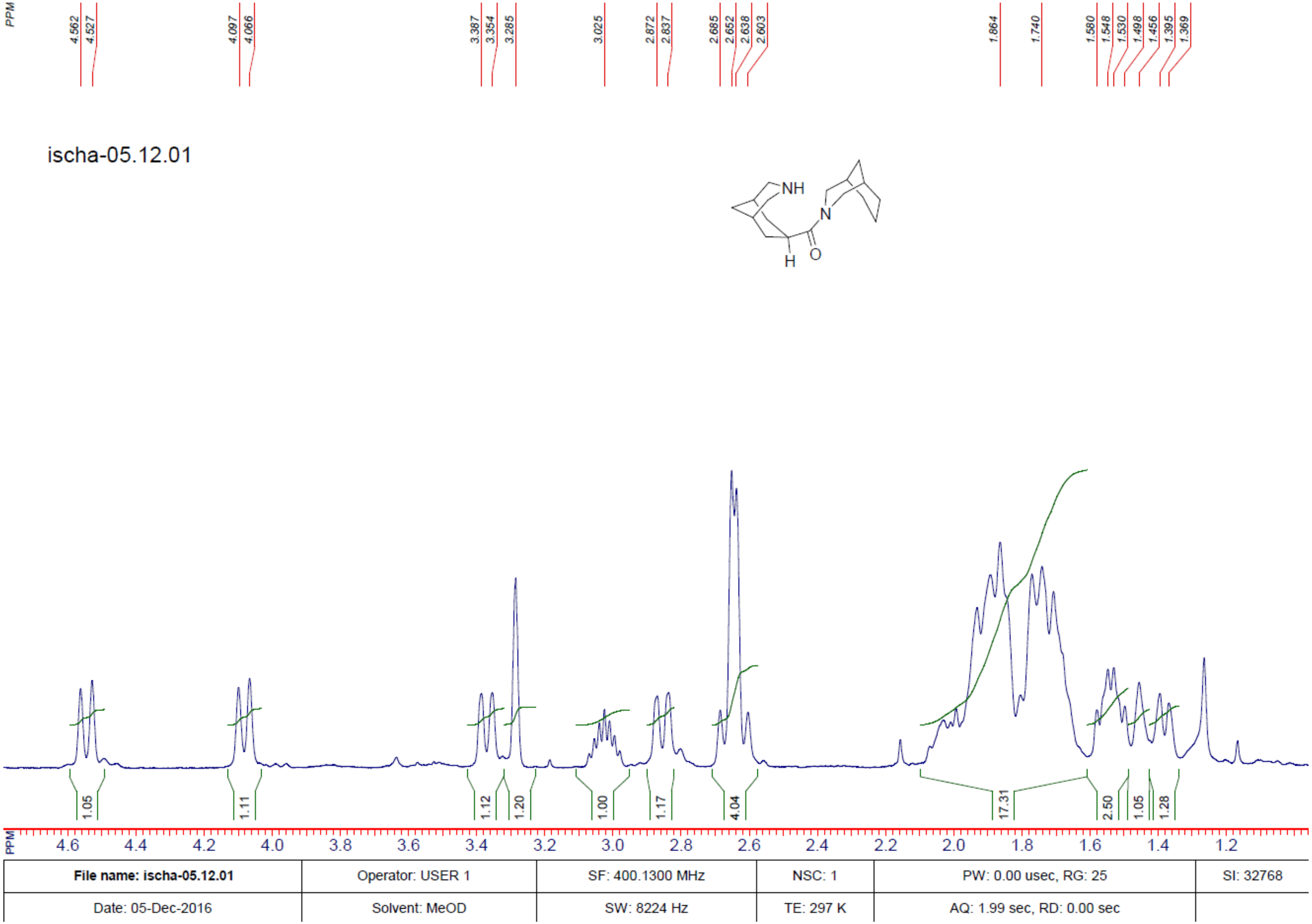

Figure S19. ^{1}H-NMR spectrum of compound **14**.

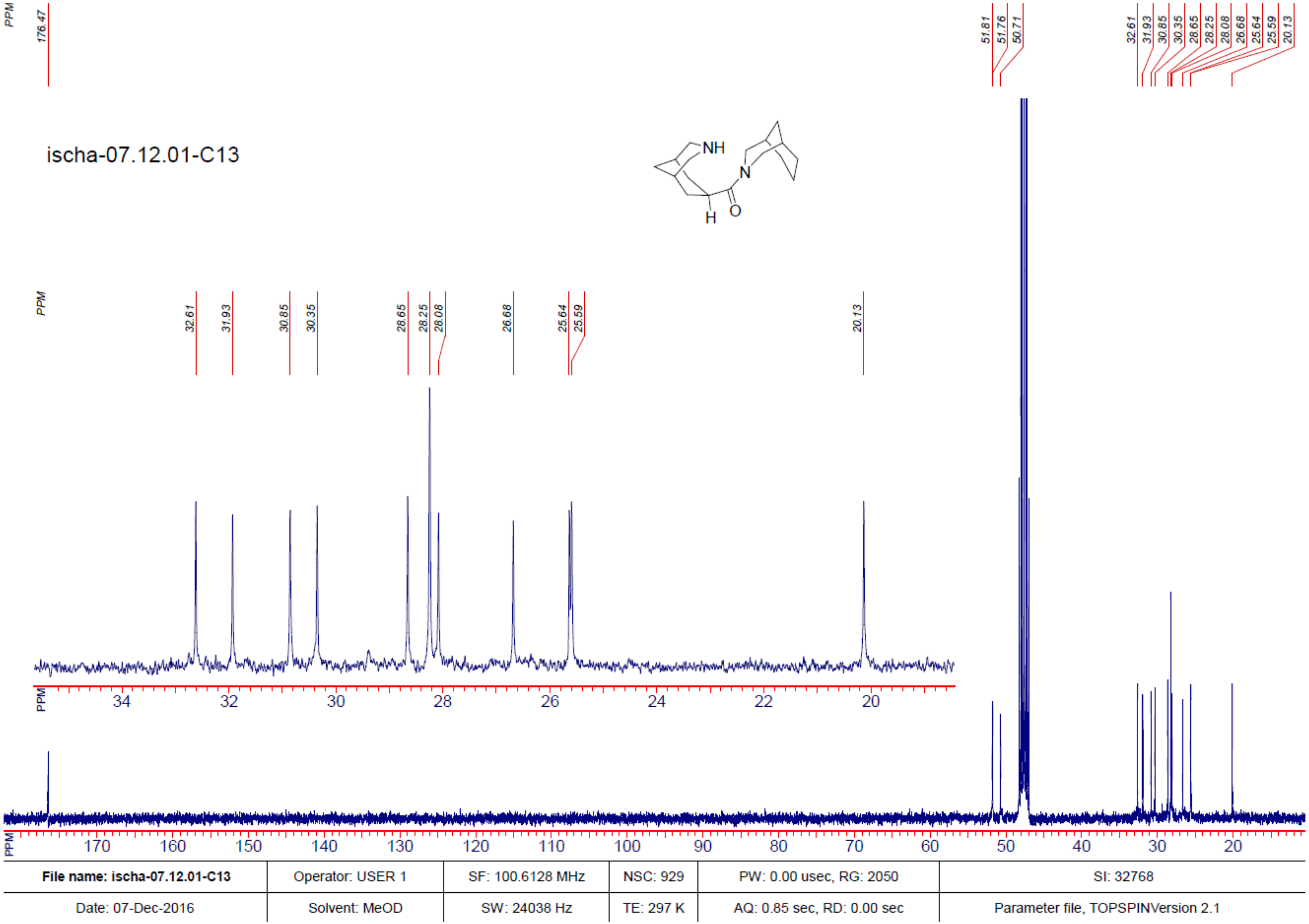

Figure S20. ^{13}C-NMR spectrum of compound **14.**

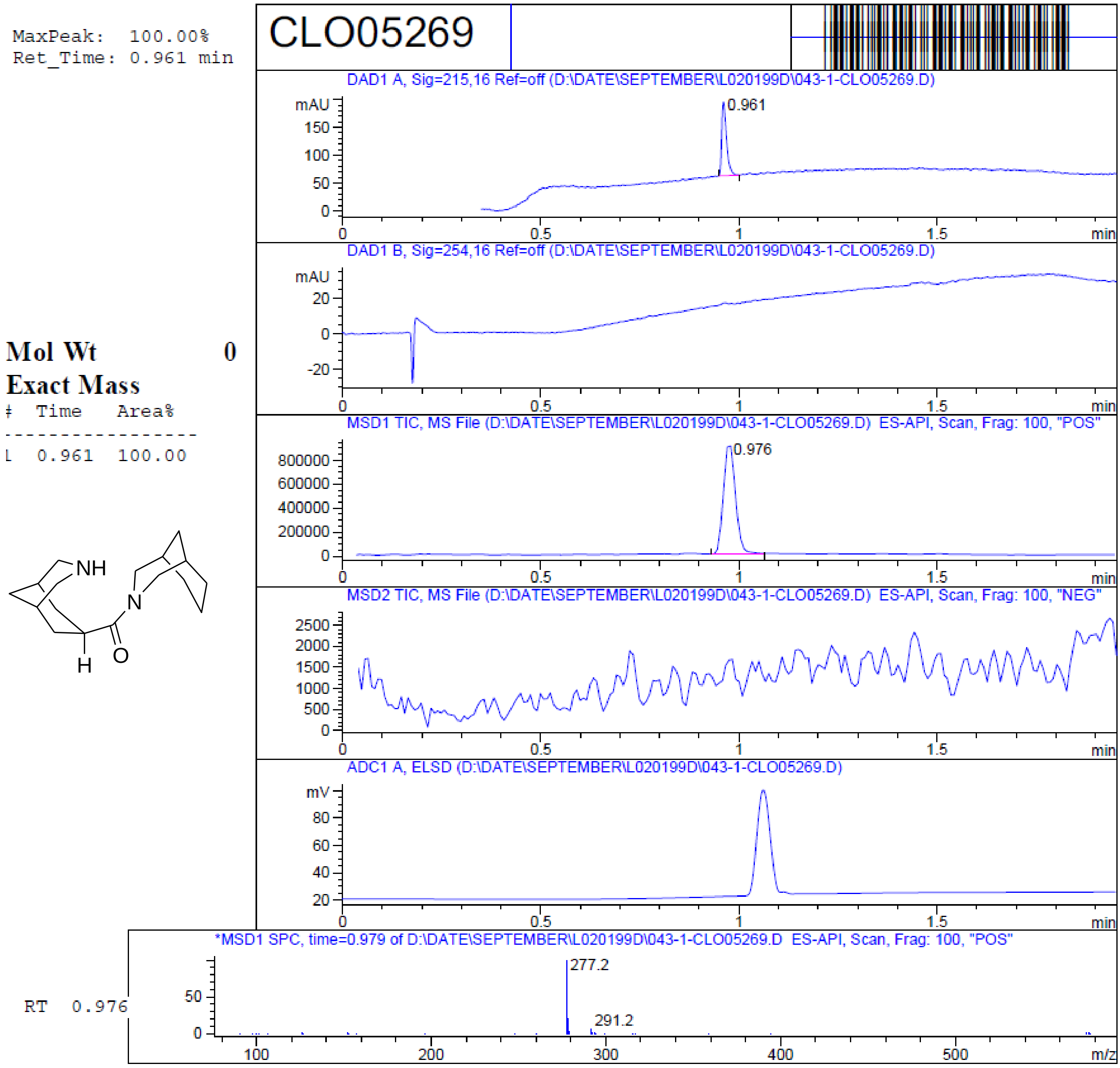

Figure S21. LC-MS trace of compound **14**.

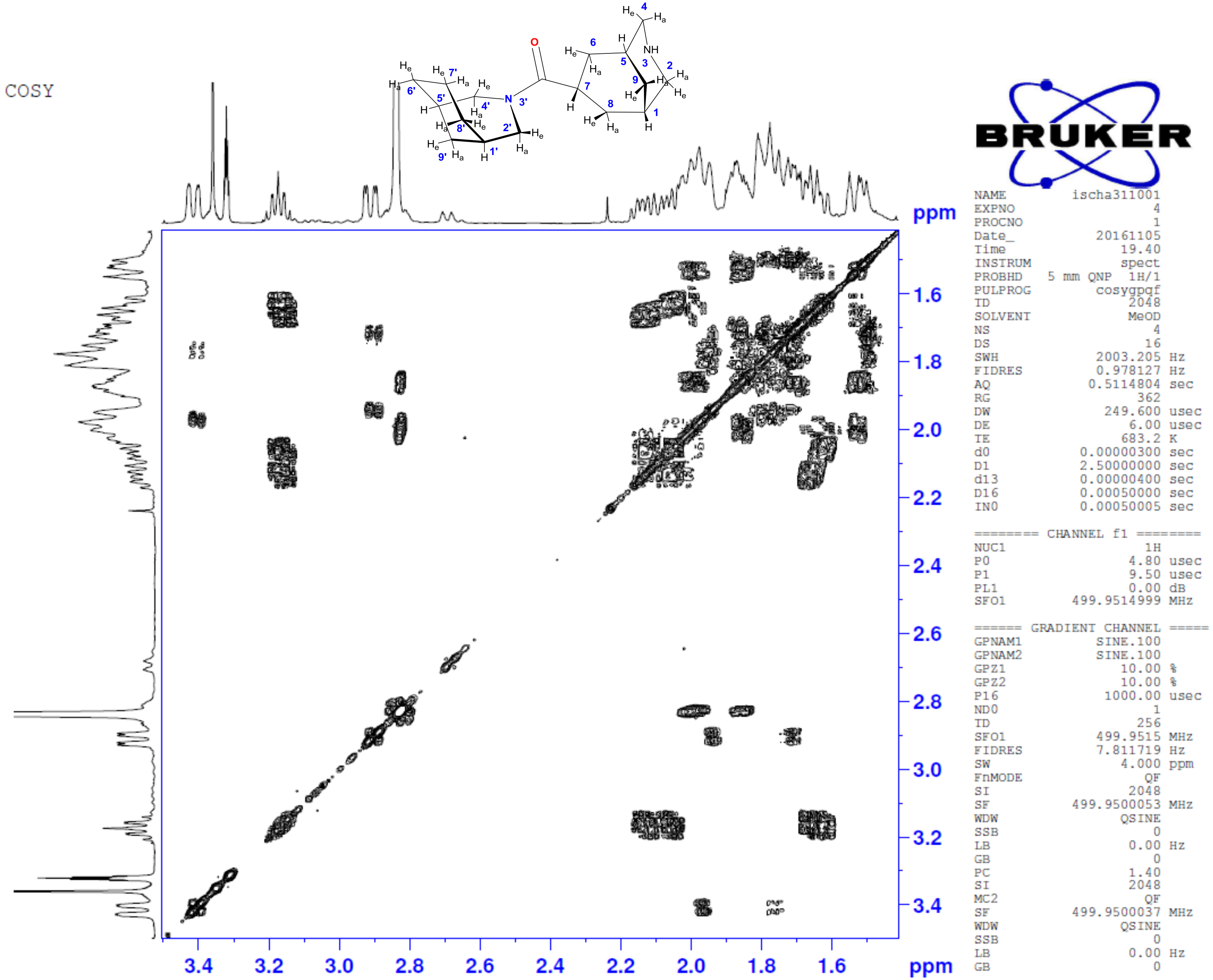

Figure S22. H,H-COSY spectrum of compound **14**.

HSQC

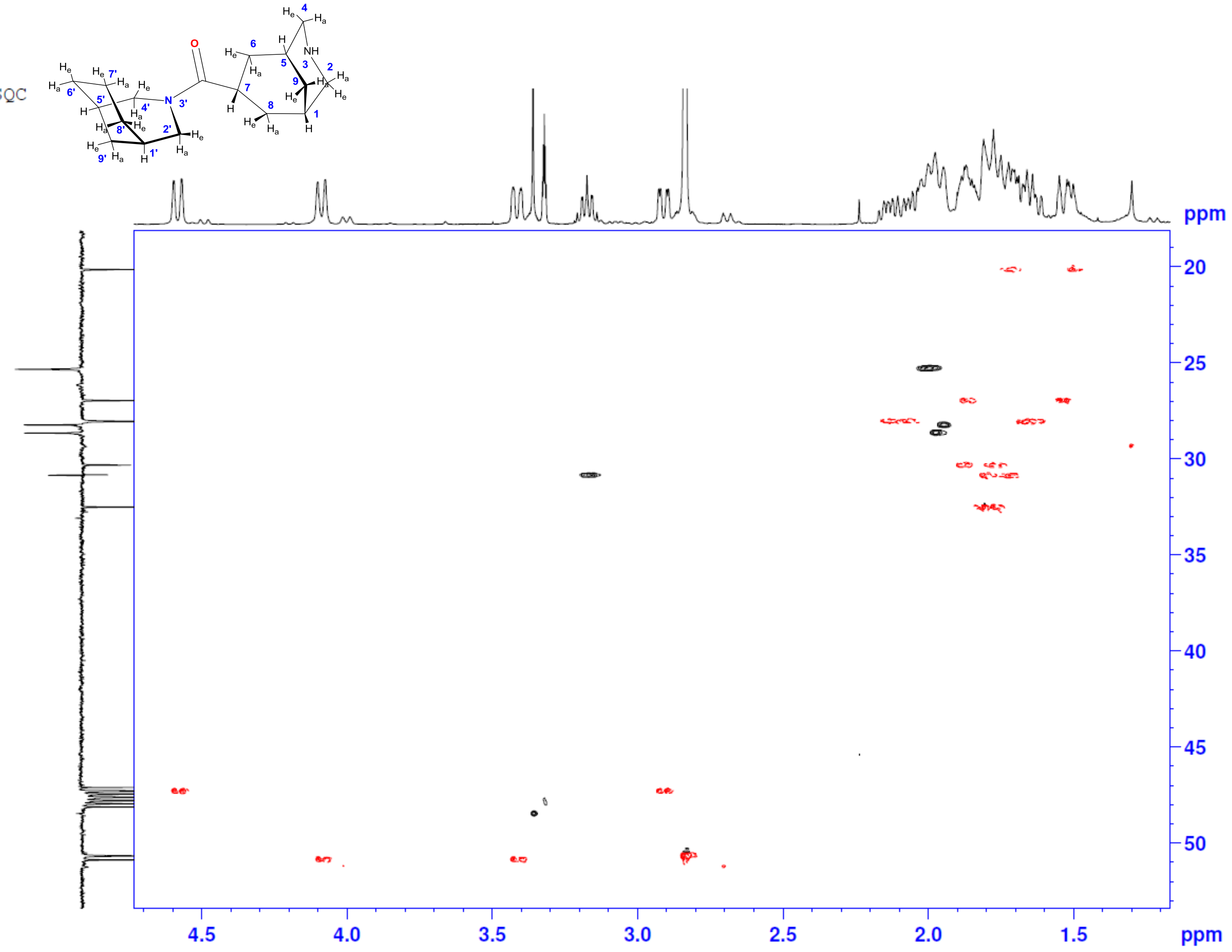

Figure S23. C,H-HSQC spectrum of compound **14**.

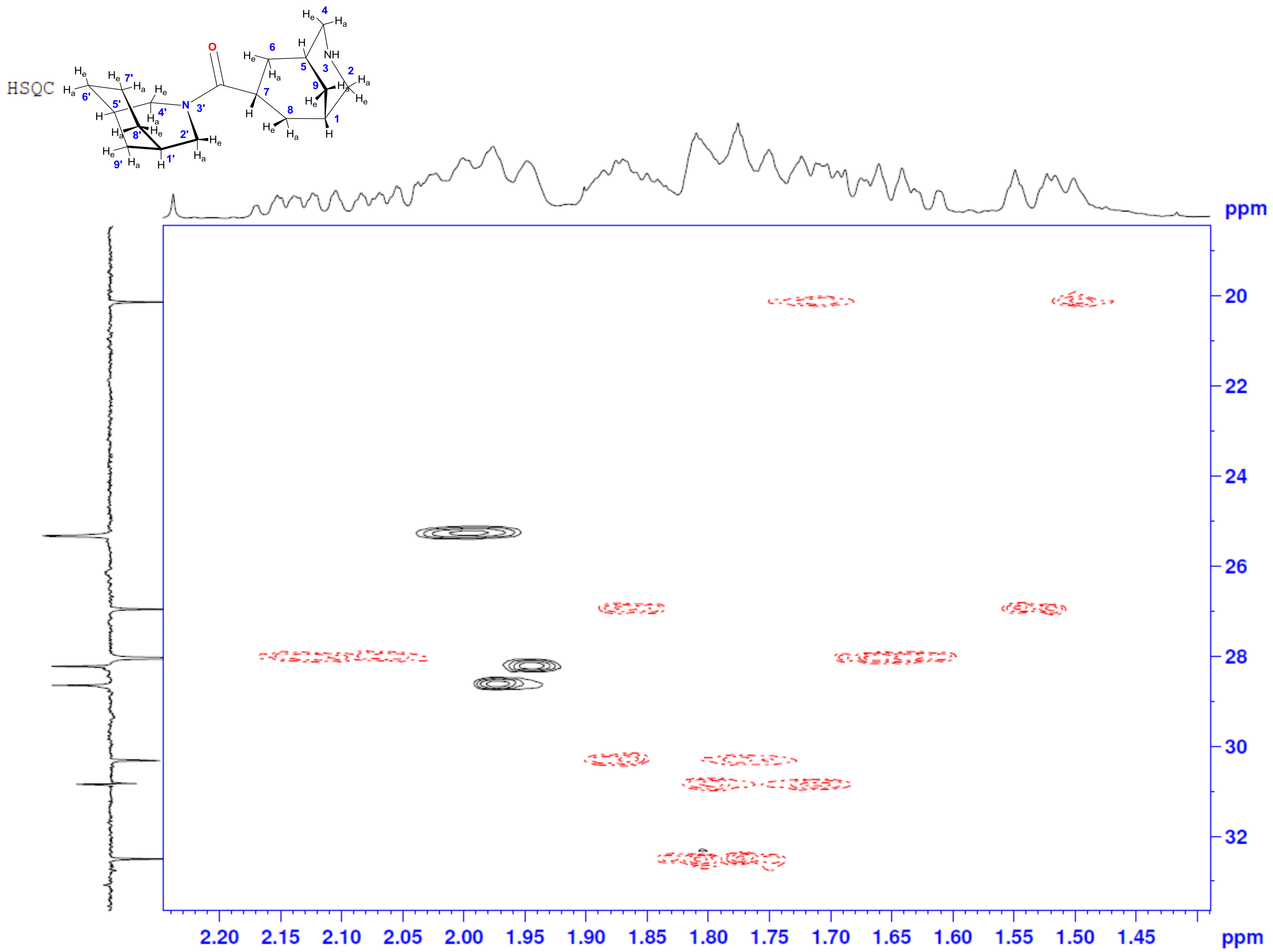

Figure S24. C,H-HSQC spectrum of compound **14** (expansion).

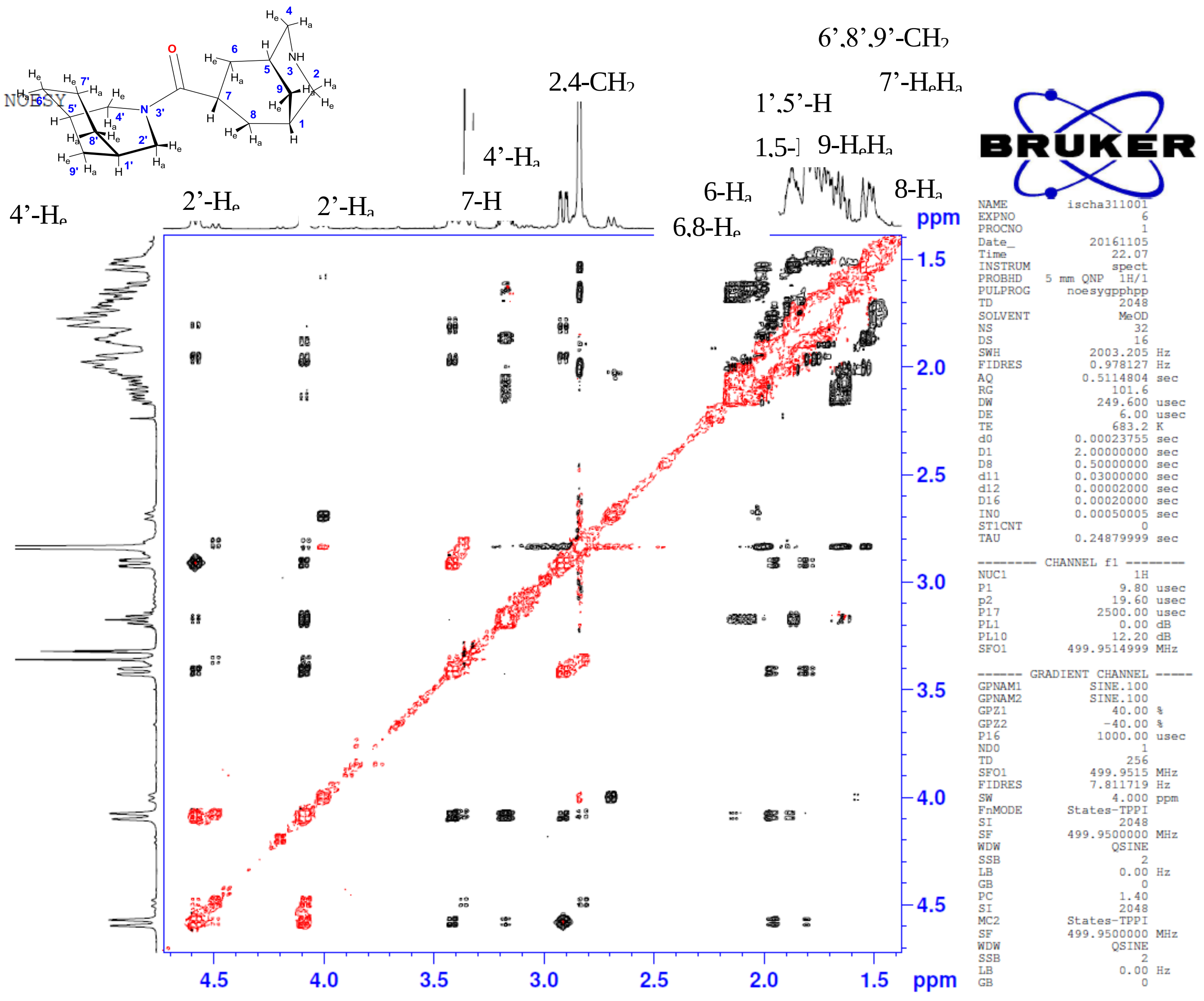

Figure S25. NOESY spectrum of compound **14**.

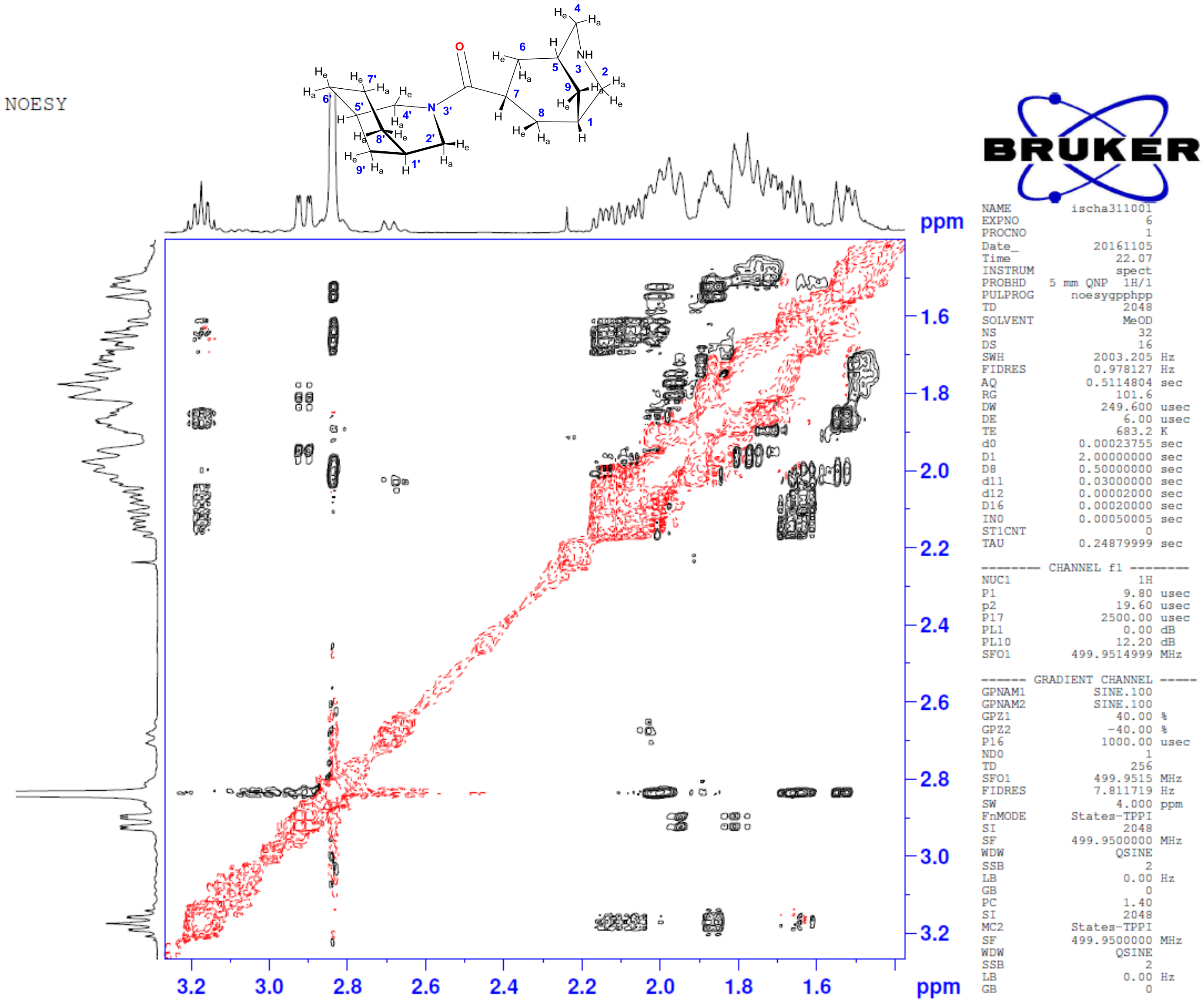

Figure S26. NOESY spectrum of compound **14** (expansion).

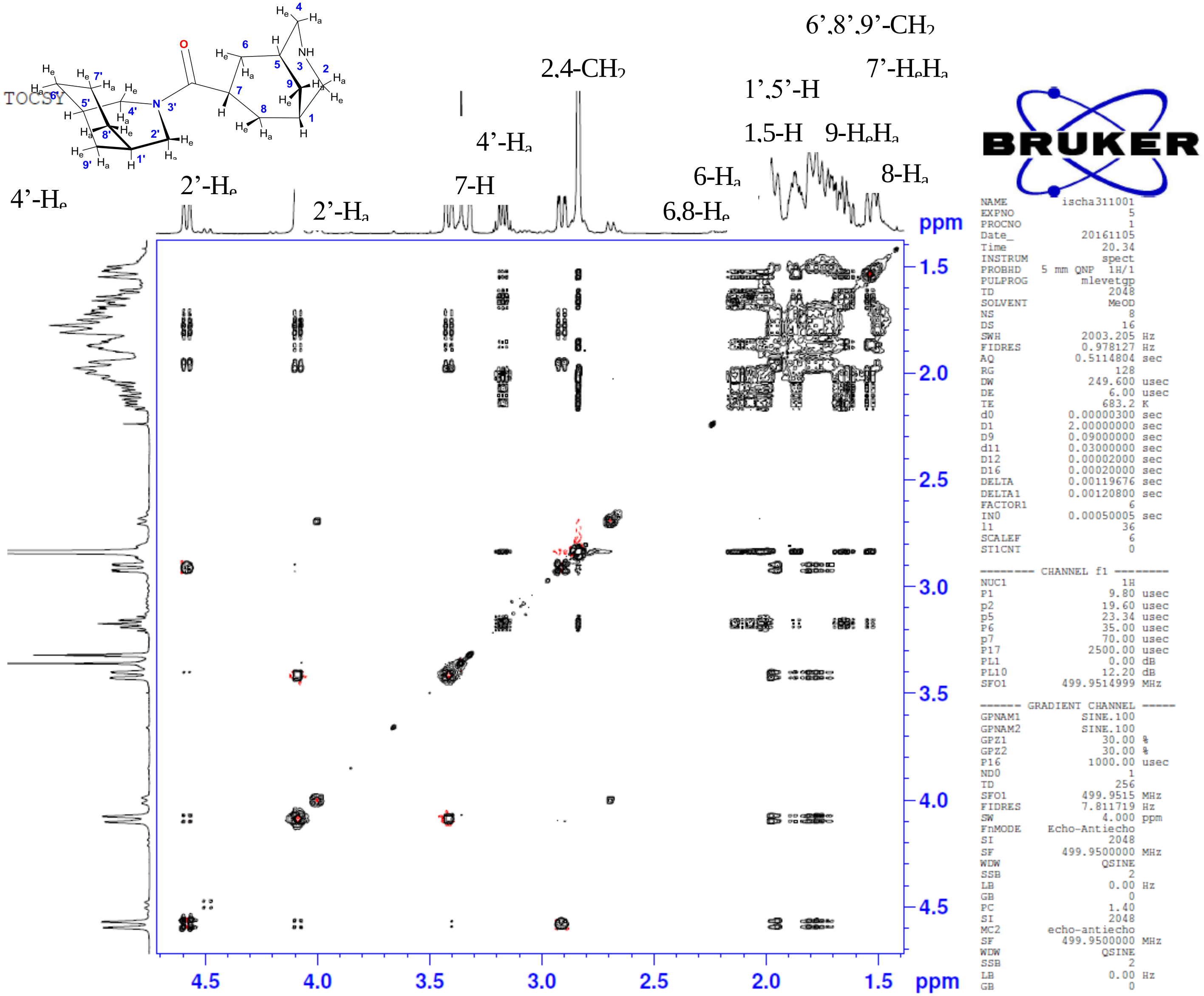

Figure S27. TOCSY spectrum of compound **14**.

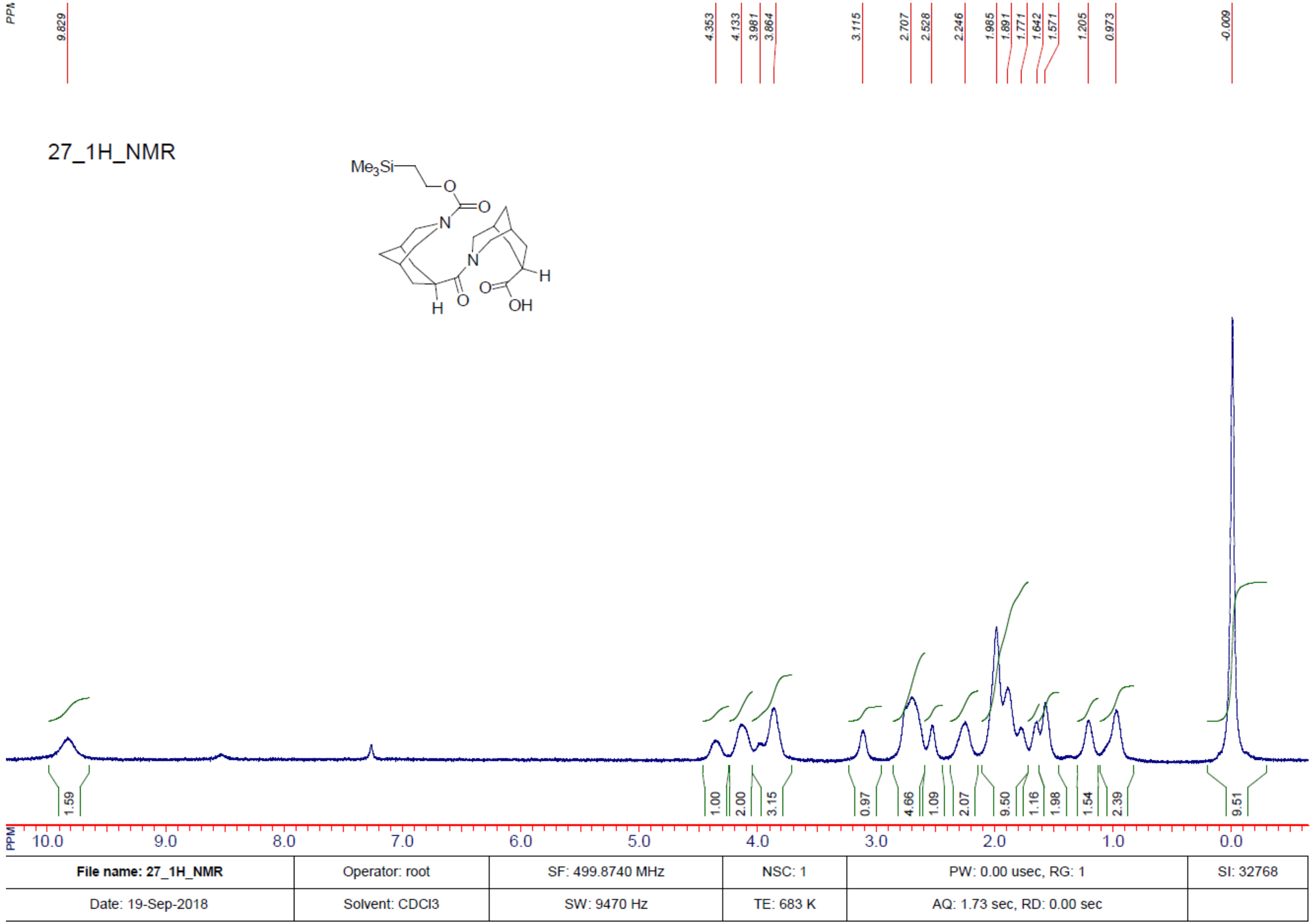

Figure S28. ^{1}H-NMR spectrum of compound **27**.

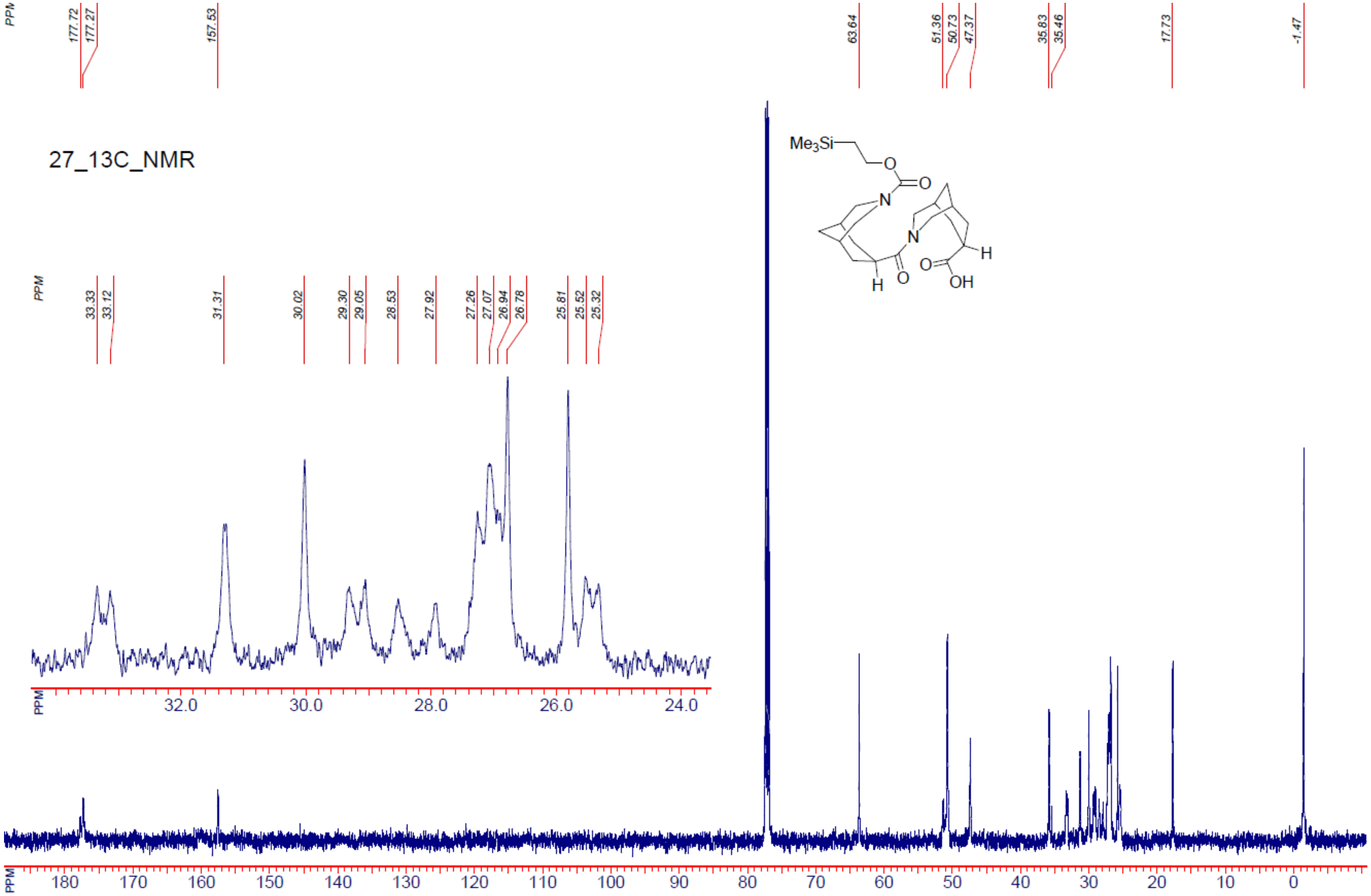

**File name: 27_13C_NMR**	Operator: root	SF: 125.6934 MHz	NSC: 200	PW: 0.00 usec, RG: 51200	SI: 65536
Date: 19-Sep-2018	Solvent: CDCl3	SW: 32680 Hz	TE: 683 K	AQ: 1.57 sec, RD: 0.00 sec	

Figure S29. ^{13}C-NMR spectrum of compound **27**.

MaxPeak: 96.00%
Ret_Time: 1.592 min

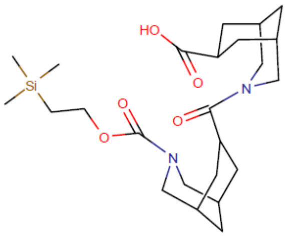

Mol Wt 464.67

Exact Mass 464.33

#	Time	Area%
1	1.592	96.00
2	1.615	4.00

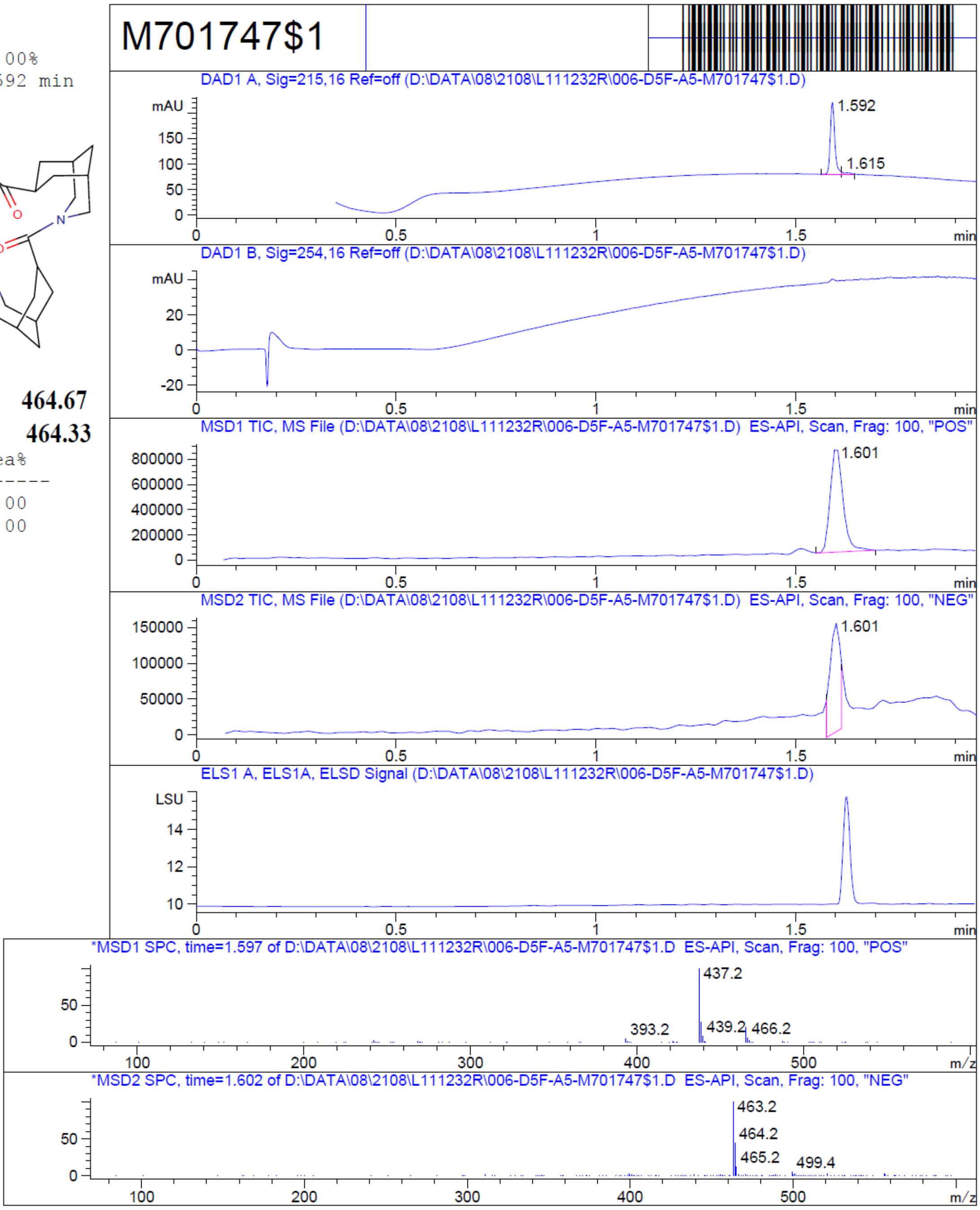

RT 1.601

RT 1.601

Inj.Date 8/21/2018 LB <invalid> -16- Acq. Method C:\Chem32\-> ->

Figure S30. LC-MS trace of compound **27**.

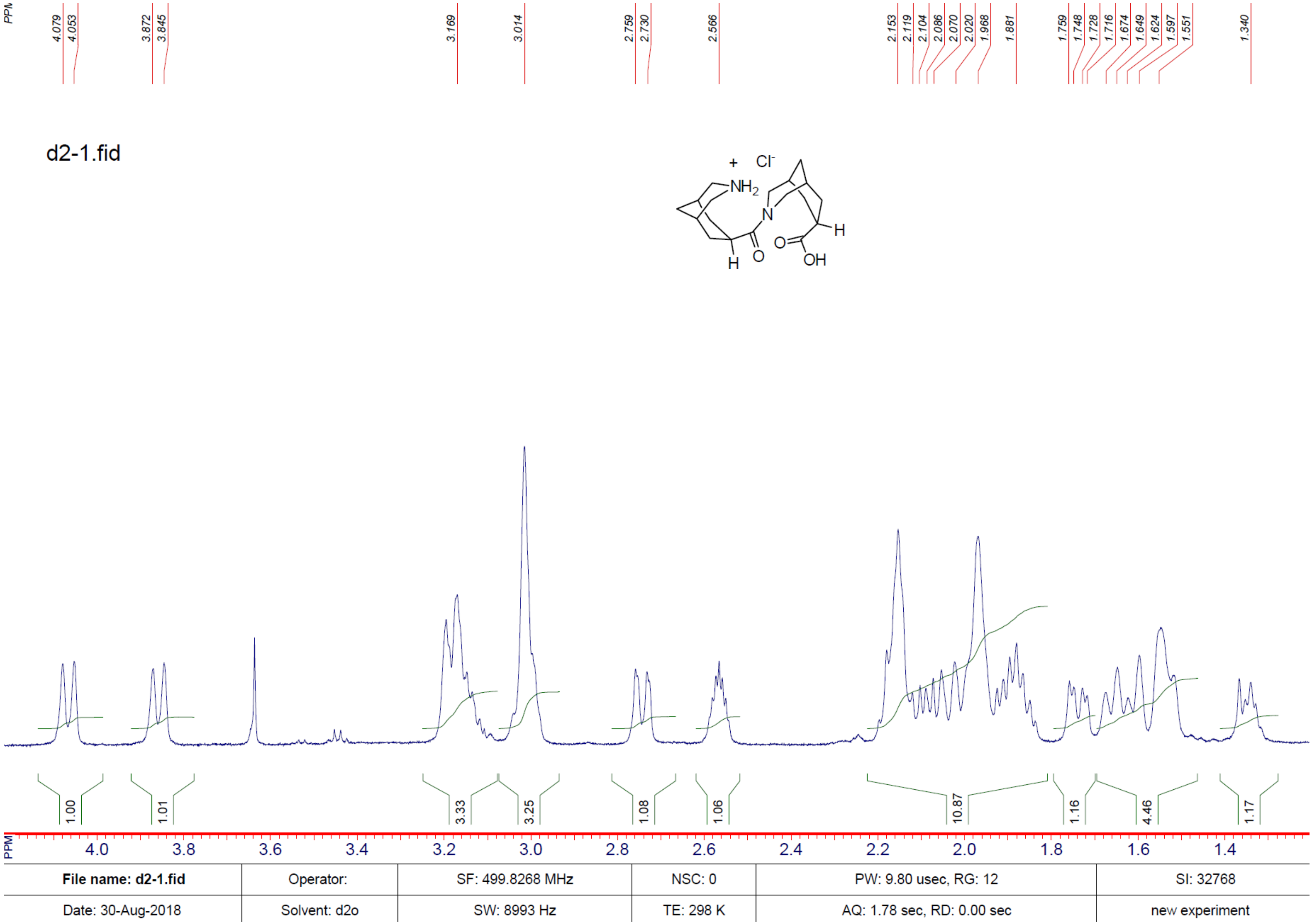

Figure S31. ^{1}H-NMR spectrum of compound **2**·HCl.

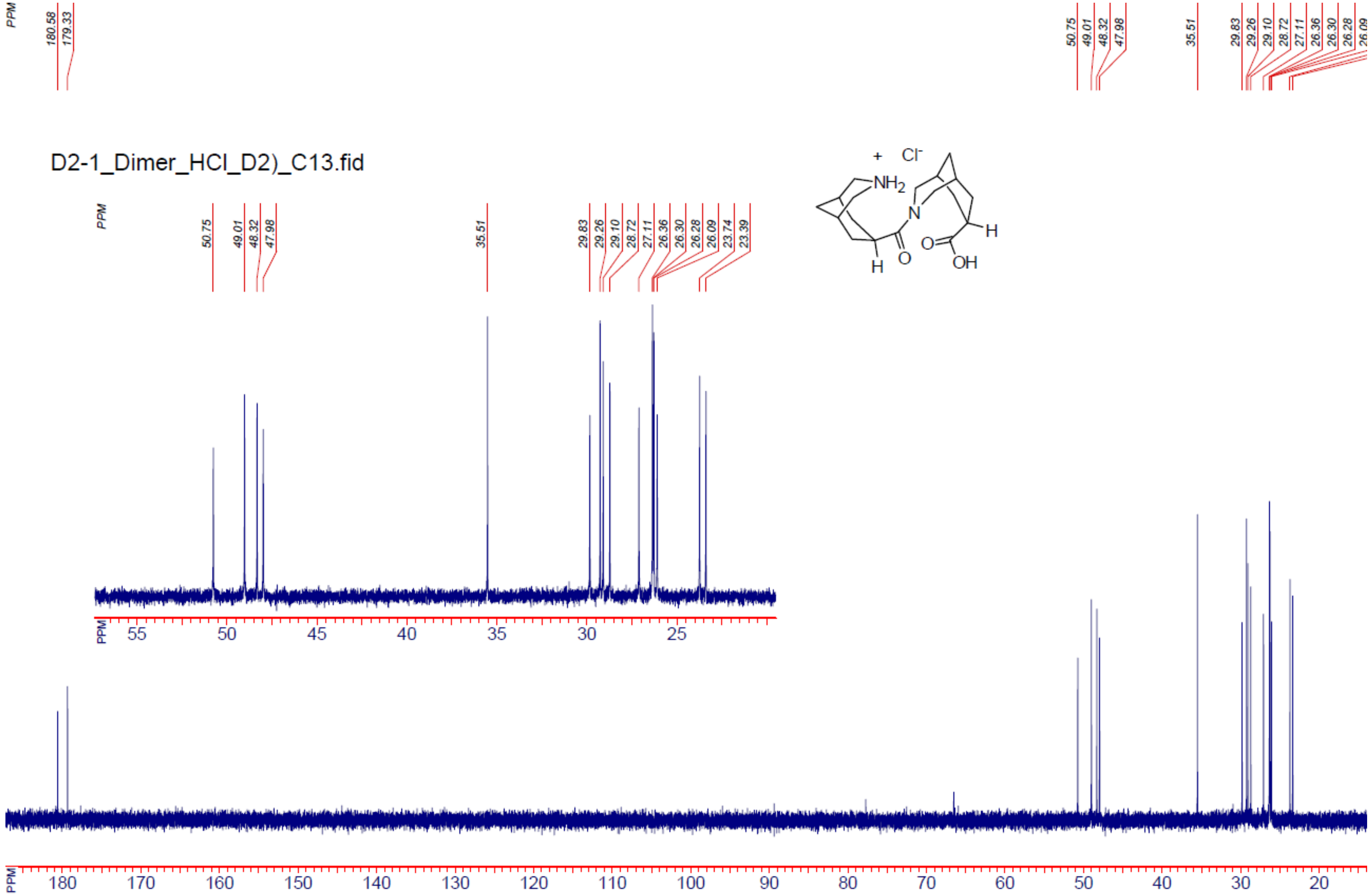

**File name: D2-1_Dimer_HCl_D2)_C13.fid**	Operator:	SF: 125.6957 MHz	NSC: 0	PW: 3.33 usec, RG: 60	SI: 65536
Date: 30-Aug-2018	Solvent: d2o	SW: 32895 Hz	TE: 298 K	AQ: 0.97 sec, RD: 0.00 sec	Automated Probe tuning parameter

Figure S32. ^{13}C-NMR spectrum of compound **2**·HCl.

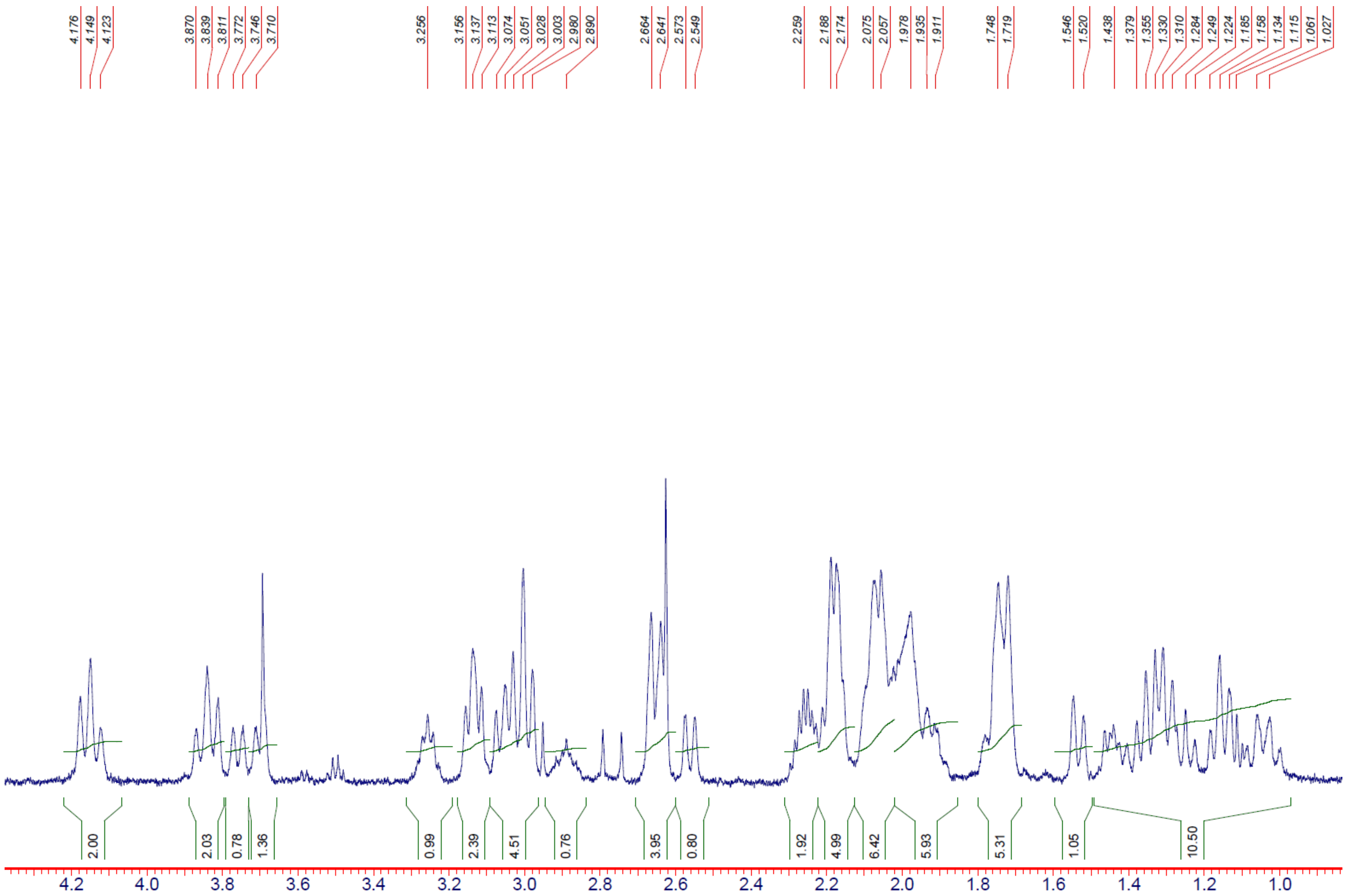

Figure S33. ^{1}H-NMR spectrum of the solution of **2** in carbonate D_2O buffer (pD 10.68) immediately after the preparation.

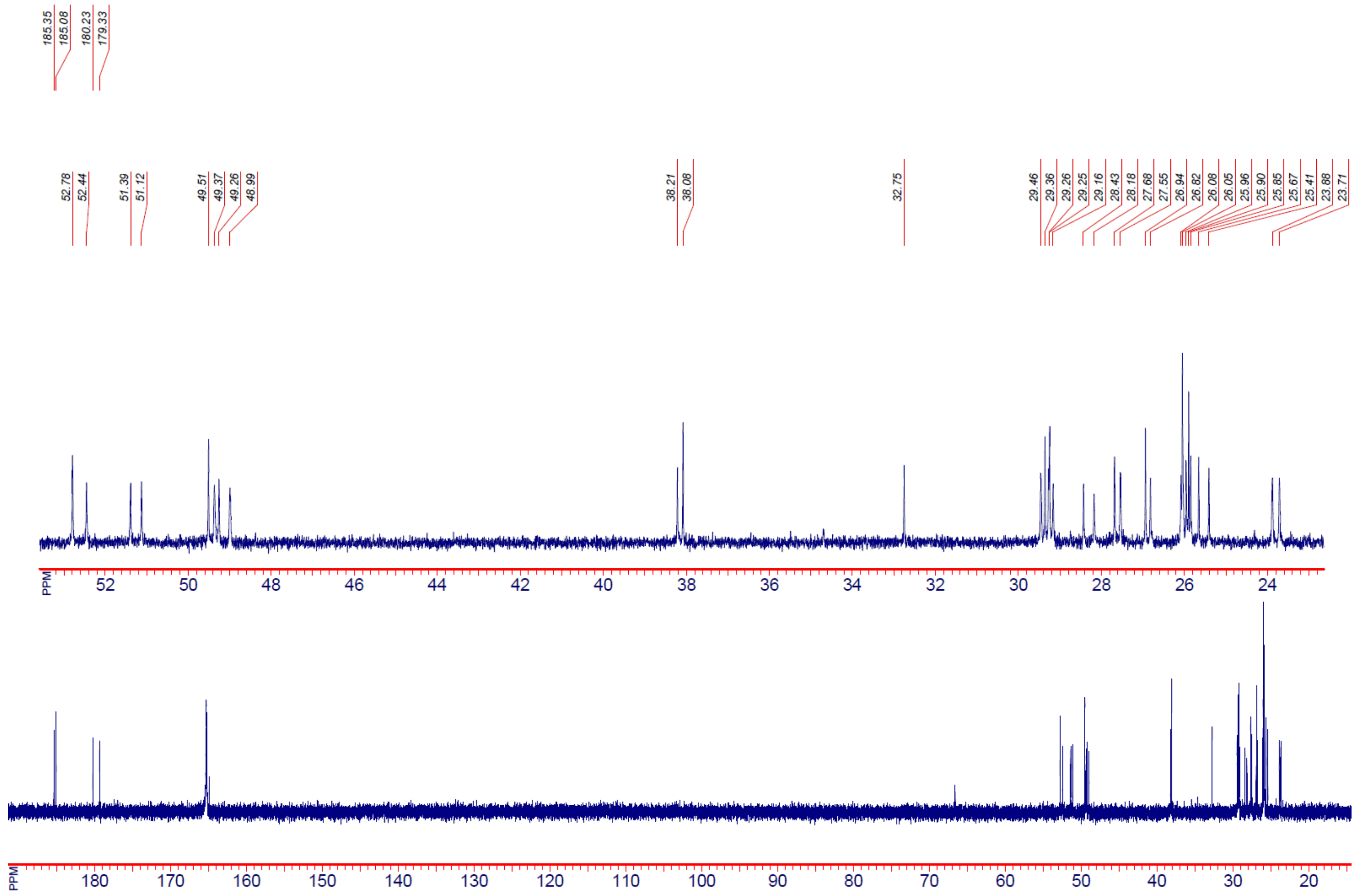

Figure S34.^{13}C-NMR spectrum of the solution of **2** in carbonate D_2O buffer (pD 10.68) immediately after the preparation.

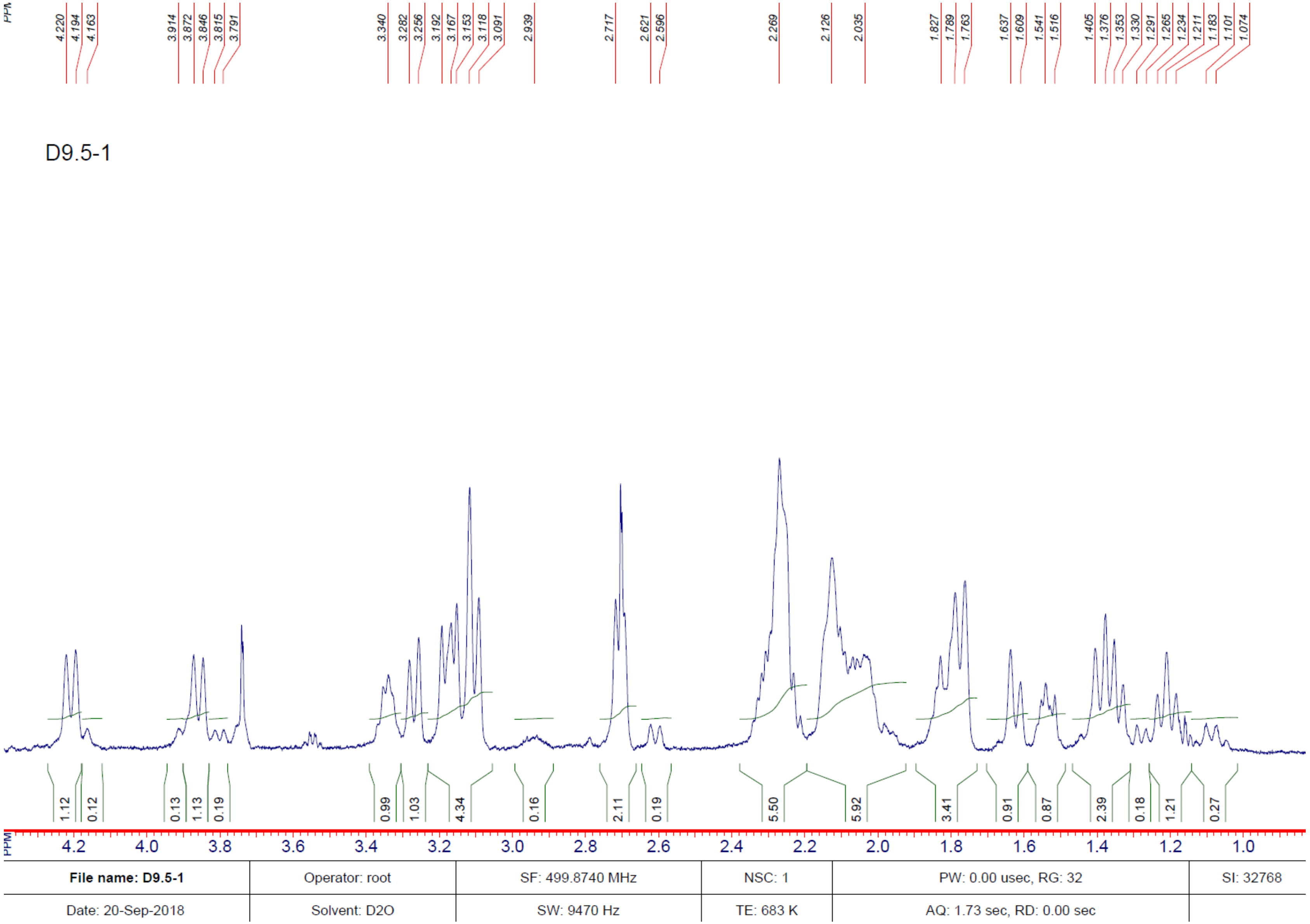

Figure S35.^{1}H-NMR spectrum of the solution of **2** in carbonate D_2O buffer (pD9.45) immediately after the preparation.

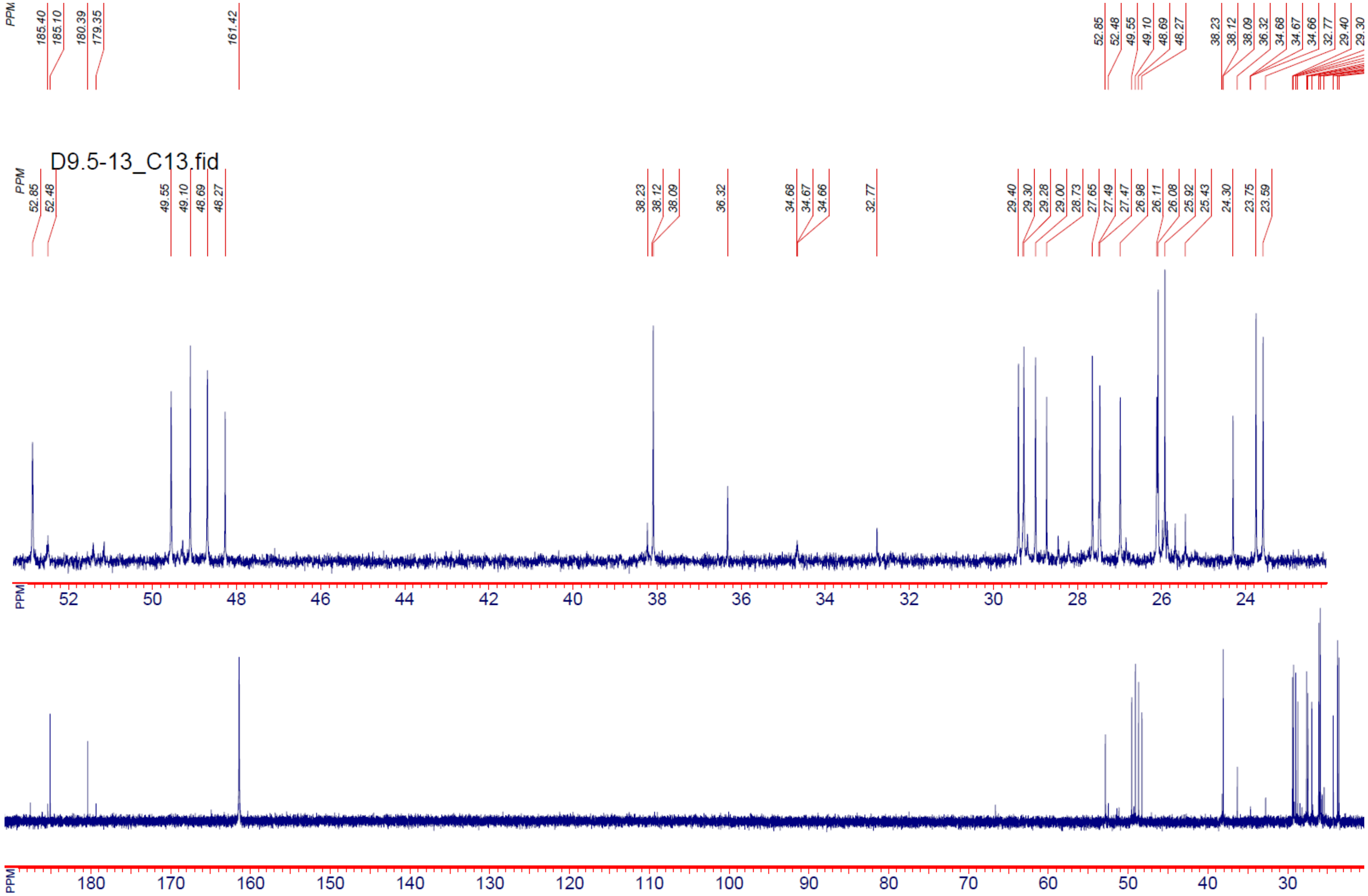

**File name: D9.5-13_C13.fid**	Operator:	SF: 125.6957 MHz	NSC: 0	PW: 3.33 usec, RG: 60	SI: 65536
Date: 10-Oct-2018	Solvent: d2o	SW: 32895 Hz	TE: 298 K	AQ: 0.97 sec, RD: 0.00 sec	Automated Probe tuning parameter

Figure S36.^{13}C-NMR spectrum of the solution of **2** in carbonate D_2O buffer (pD9.45) immediately after the preparation.

Kinetic data on the hydrolysis of 2·HCl in acetate D_2O buffer, pD 3.81.

Table S1. Molar percent of **2**·HCl vs time (min) in acetate buffer (pD 3.81, 23 °C)

Time, min	0	273	3105	4606	7168	10950	15848	16127	17131
Molar % of **2**·HCl	100	97	81	79	74	61	57	51	49

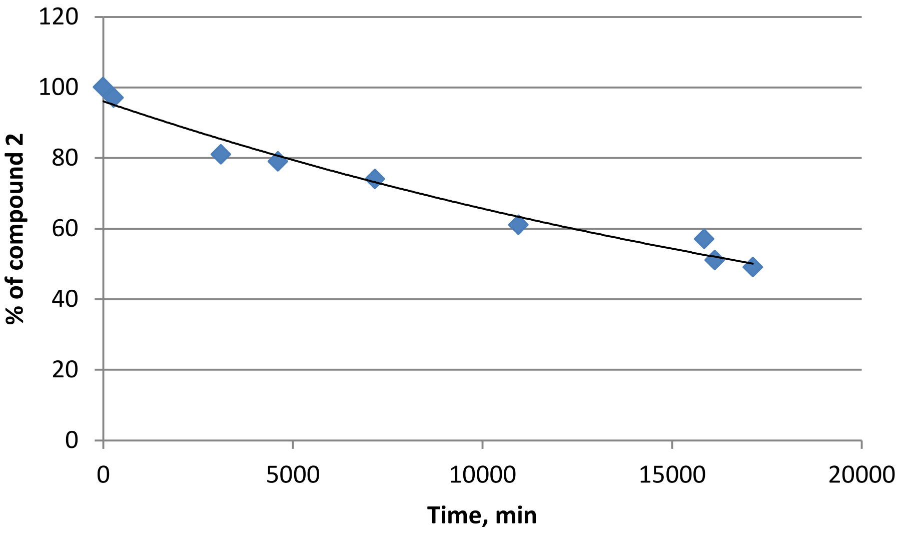

Figure S37. Exponential fit of the data (Table S1).

Table S2. Fitting parameters and corresponding kinetic constants.

Equation	y0	A1	t1	R^2	Half-life, $t_{1/2}$, min	k_{obs}, min^{-1}	$\log(k_{obs})$
y = A1·exp(-x/t1) + y0	34,43121± 11,0332	63,99936± 10,30033	12830,87499± 4149,08408	0,97726	18138	$3.821 \cdot 10^{-5}$	-4.418

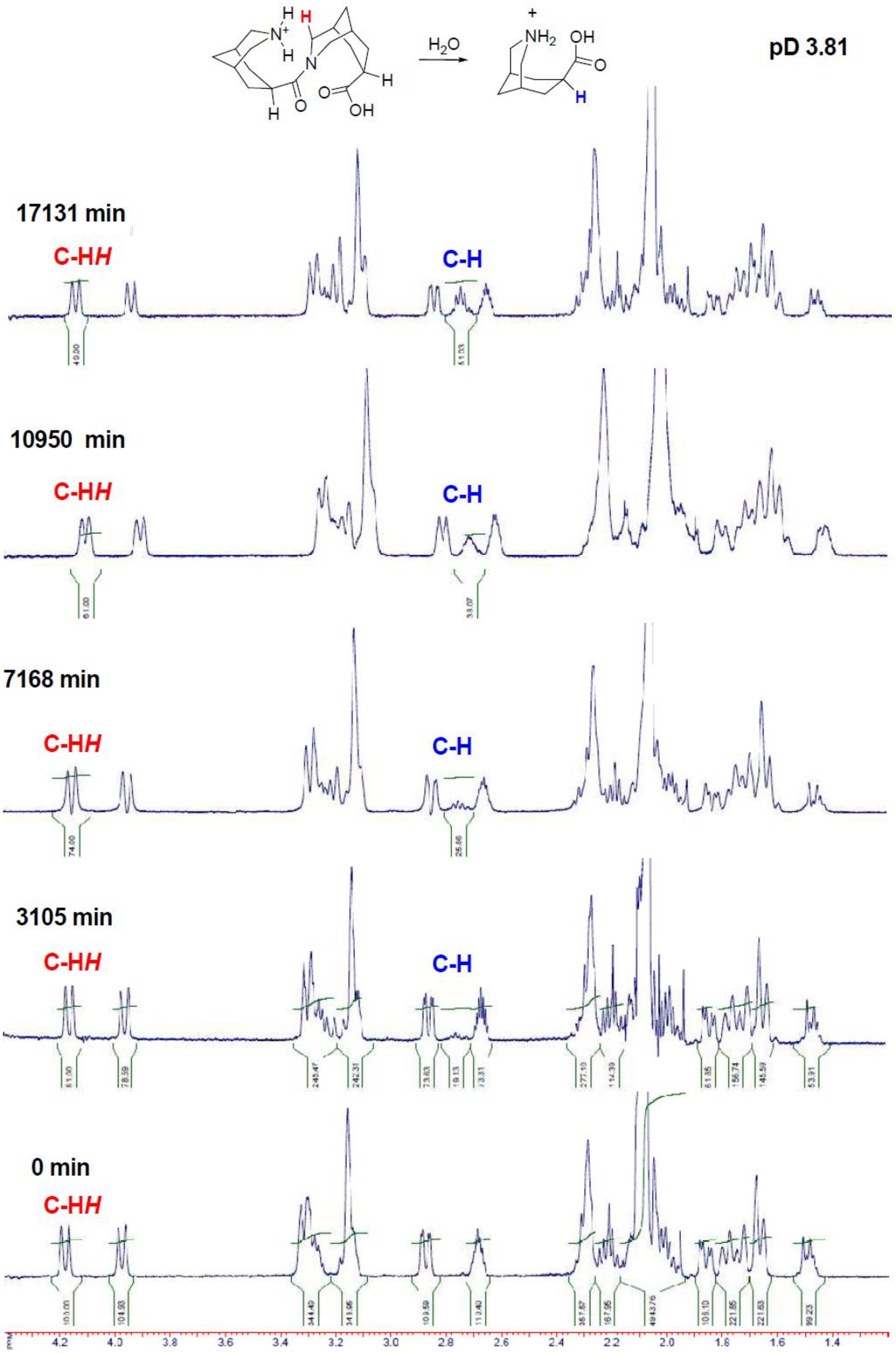

Figure S38. Representative spectral data set for hydrolysis of **2**·HCl in acetate D_2O buffer, pD 3.81, 23 °C.

Kinetic data on the hydrolysis of 2·HCl in acetate D_2O buffer, pD4.85.

Table S3. Molar percent of **2**·HCl vs time (min) in acetate buffer (pD 4.85, 23 °C)

Time, min	0	275	3110	4609	7150	10953	13148	16191	17136
Molar % of **2**·HCl	100	96	62	45	39	23	20	11	5

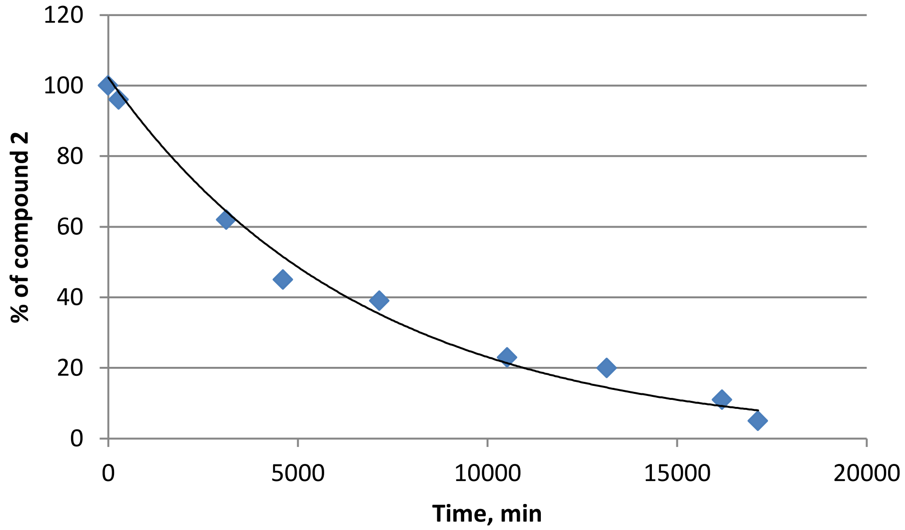

Figure S39. Exponential fit of the data (Table S3).

Table S4. Fitting parameters and corresponding kinetic constants.

Equation	y0	A1	t1	R^2	Half-life, $t_{1/2}$, min	k_{obs}, min^{-1}	$\log(k_{obs})$
y = A1·exp(-x/t1) + y0	2,69584± 4,7715	96,60028± 4,81107	6537,81499± 895,81784	0,98792	4668	$1.485 \cdot 10^{-4}$	-3.828

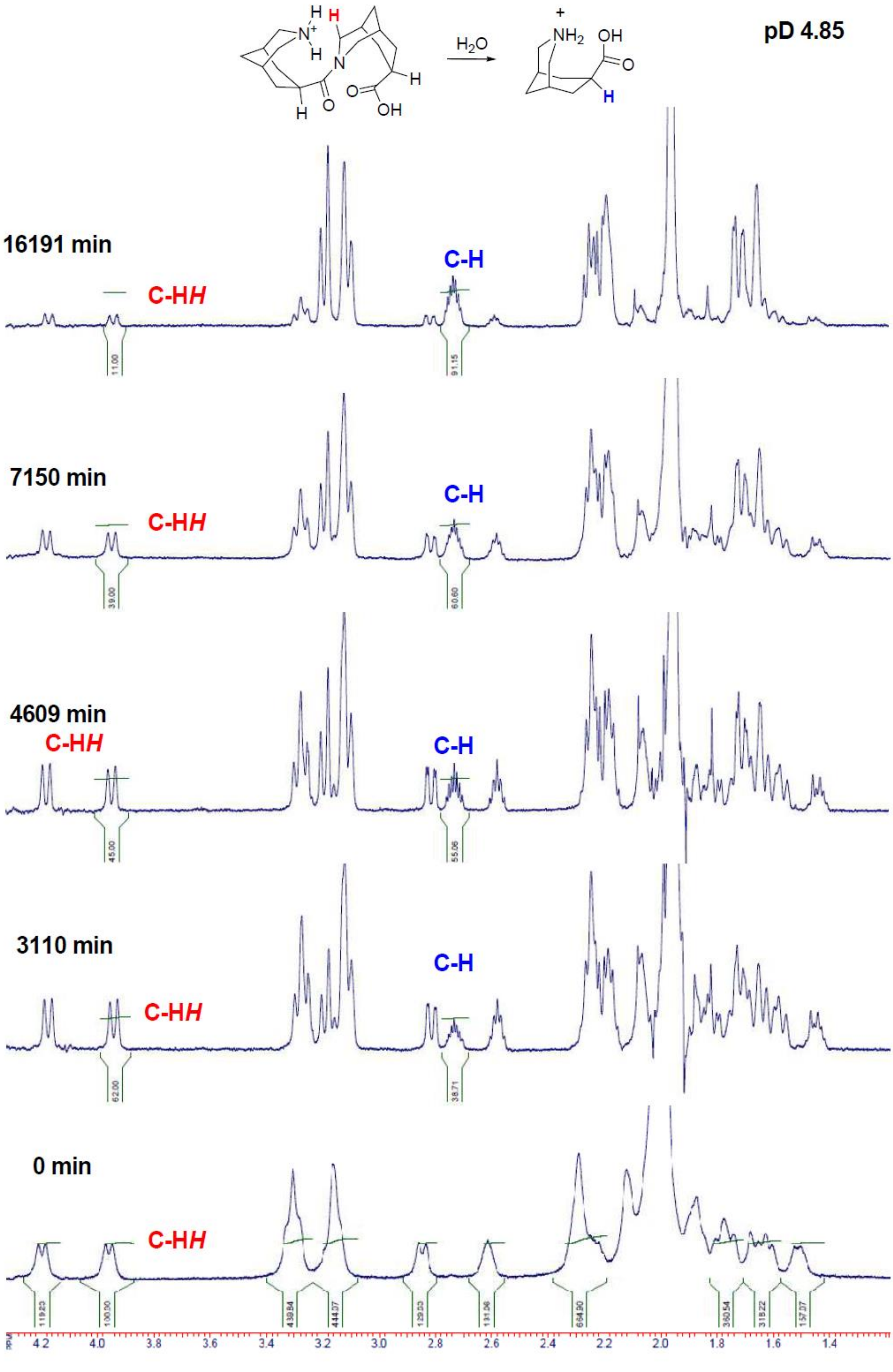

Figure S40. Representative spectral data set for hydrolysis of **2**·HCl in acetate D_2O buffer, pD4.85, 23 °C.

Kinetic data on the hydrolysis of 2·HCl in phosphateD$_2$O buffer, pD6.68.

Table S5. Molar percent of **2**·HCl vs time (min) in phosphate buffer (pD6.68, 23 °C)

Time, min	0	60	313	1745	3302	5871	9645	11844	14884
Molar % of **2**·HCl	100	98	96	69	56	32	28	17	12

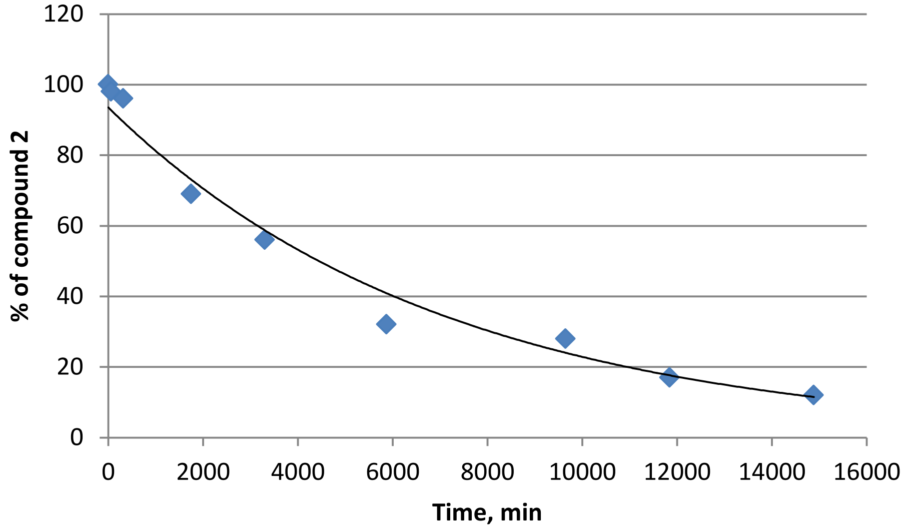

Figure S41. Exponential fit of the data (Table S5).

Table S6. Fitting parameters and corresponding kinetic constants.

Equation	y0	A1	t1	R^2	Half-life, $t_{1/2}$, min	k_{obs}, min^{-1}	log(k_{obs})
y = A1·exp(-x/t1) + y0	11,32544± 3,65066	88,61318± 3,75123	4520,24104± 565,27656	0,99053	3748	$1.849 \cdot 10^{-4}$	-3.733

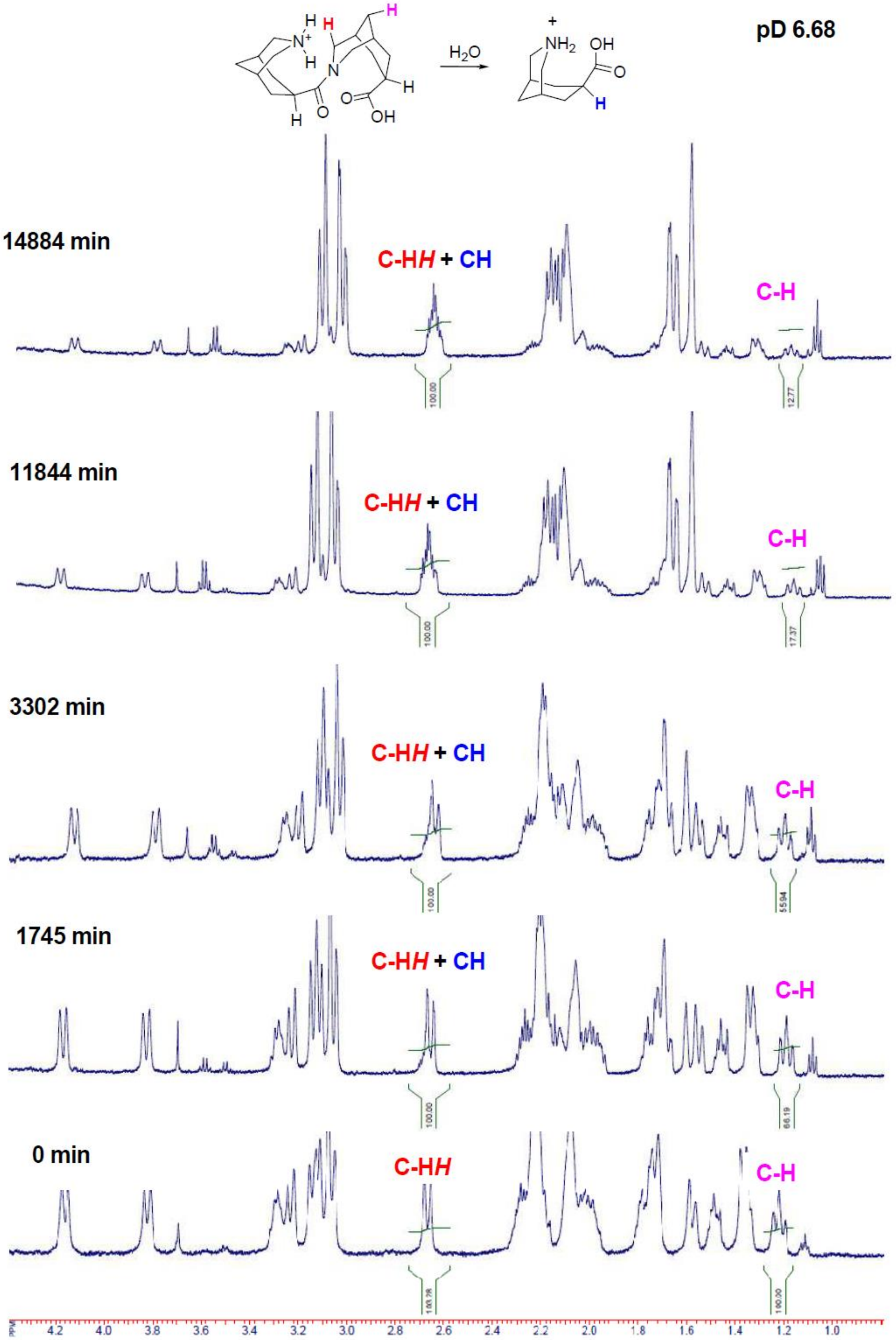

Figure S42. Representative spectral data set for hydrolysis of **2**·HCl in phosphate D_2O buffer, pD6.68, 23 °C.

Kinetic data on the hydrolysis of 2·HCl in phosphate D_2O buffer, pD7.95.

Table S7. Molar percent of **2** vs time (min) in phosphate buffer (pD7.95, 23 °C)

Time, min	0	375	746	1521	2924	5104	9022	14445
Molar % of **2**·HCl	100	95	93	90	83	78	65	55

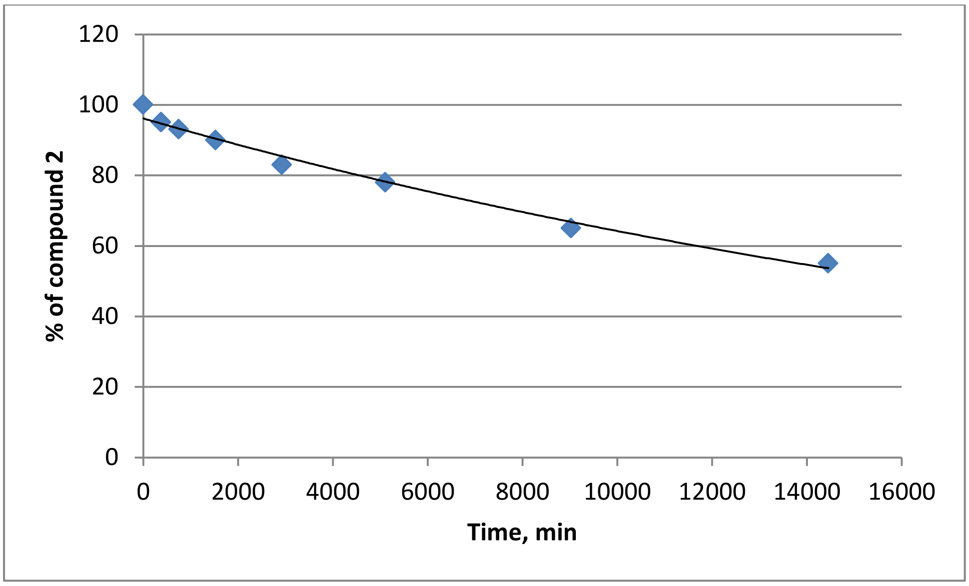

Figure S43. Exponential fit of the data (Table S7).

Table S8. Fitting parameters and corresponding kinetic constants.

Equation	y0	A1	t1	R^2	Half-life, $t_{1/2}$, min	k_{obs}, min^{-1}	$log(k_{obs})$
y = A1·exp(-x/t1) + y0	39,32703± 7,62041	58,542± 7,2248	11049,631± 2505,00153	0,99065	18802	$3.686 \cdot 10^{-5}$	-4.433

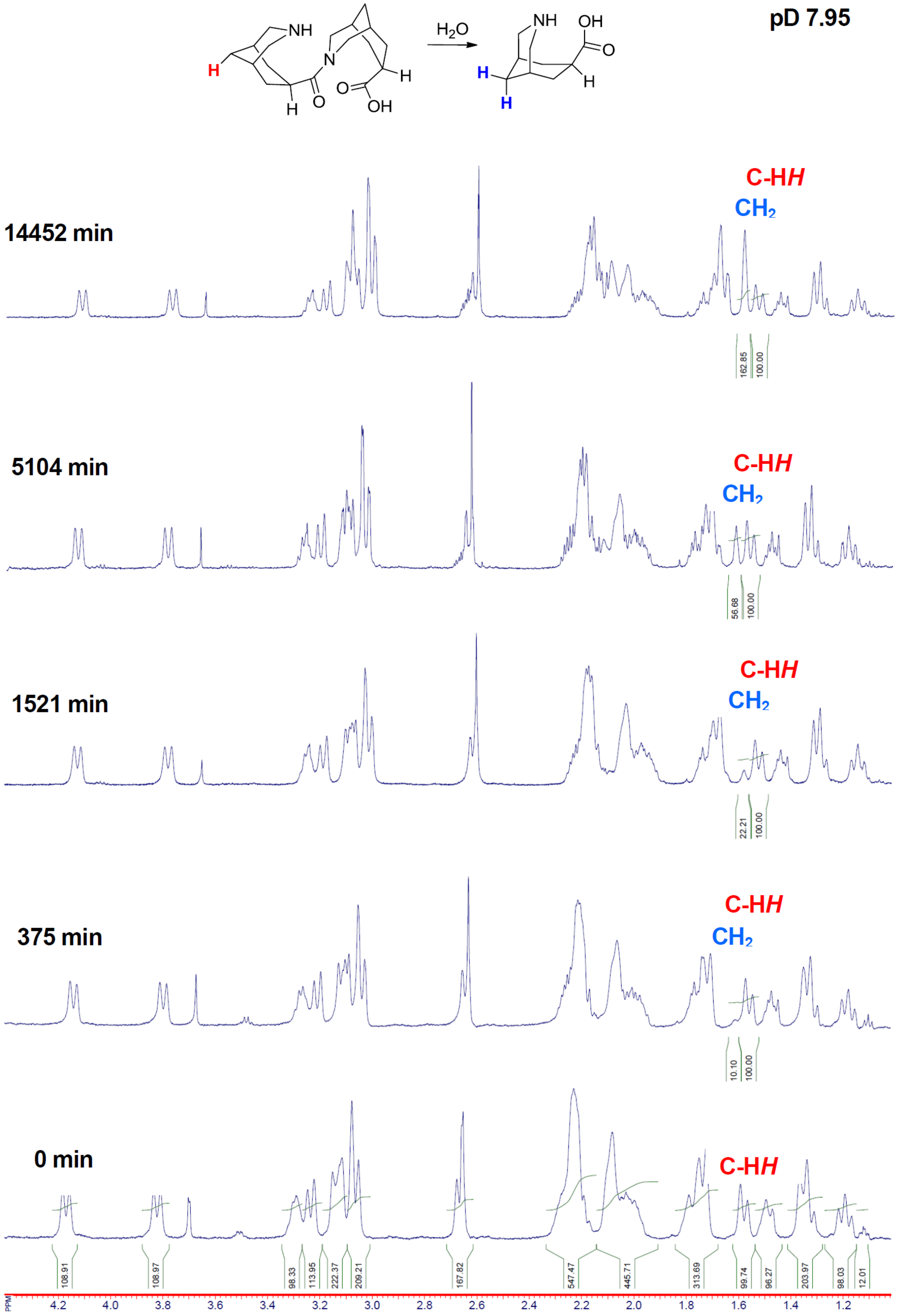

Figure S44. Representative spectral data set for hydrolysis of **2**·HCl in phosphate D_2O buffer, pD7.95, 23 °C.

Kinetic data on the hydrolysis of 2·HCl in carbonate D_2O buffer, pD 9.45.

Table S9. Molar percent of **2** vs time (min) in carbonate buffer (pD 9.45, 23 °C)

Time, min	0	1932	2864	6009	6780	8185	10368	14287	19713
Molar % of 2·HCl	100	97.5	97	96	93	92	90	85	83

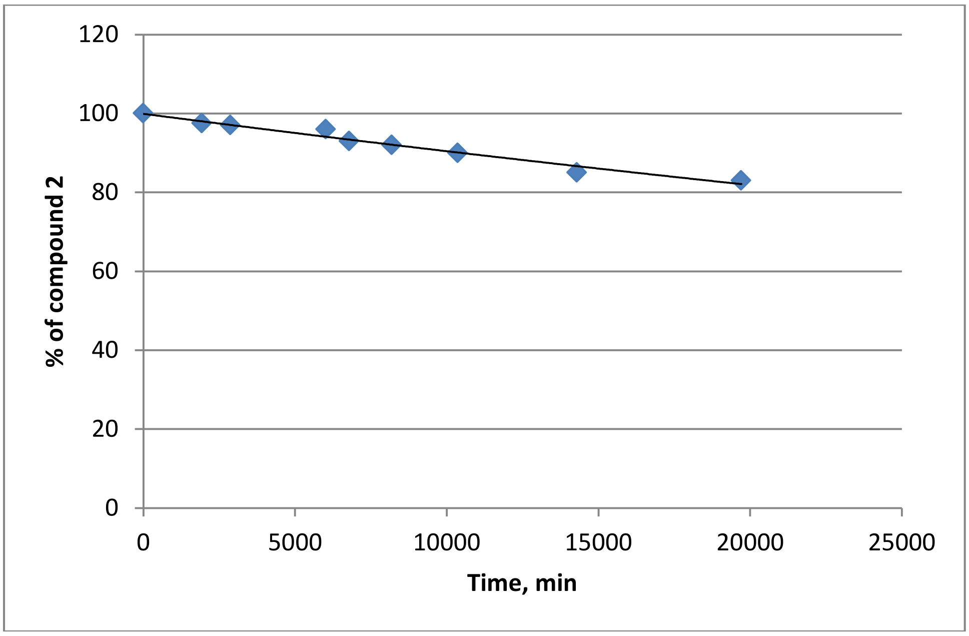

Figure S45. Exponential fit of the data (Table S9).

Table S10. Fitting parameters and corresponding kinetic constants.

Equation	y0	A1	t1	R^2	Half-life, $t_{1/2}$, min	k_{obs}, min^{-1}	log(k_{obs})
y = A1·exp(-x/t1) + y0	37,98426± 69,45674	62,1325± 68,92624	58252,386± 76398,515	0,96279	91125	$7.605 \cdot 10^{-6}$	-5.119

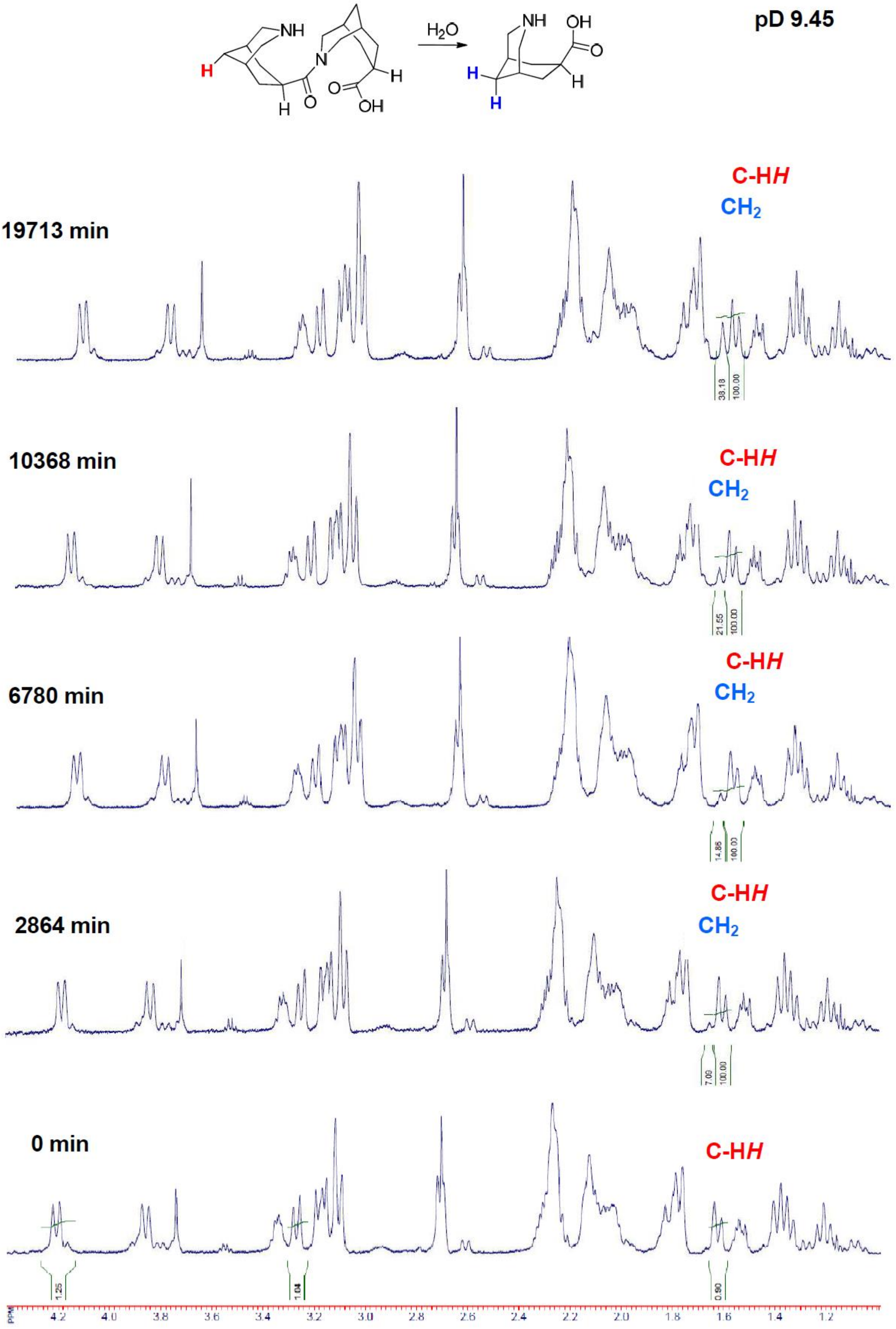

Figure S46. Representative spectral data set for hydrolysis of **2**·HCl in phosphate D_2O buffer, pD 9.45, 23 °C.

Kinetic data on the hydrolysis of 2·HCl in carbonate D_2O buffer, pD 10.68.

Table S11. Molar percent of **2** vs time (min) in carbonate buffer (pD 10.68, 23 °C)

Time, min	0	1925	2863	19625
Molar % of **2**·HCl	100	99	98	88

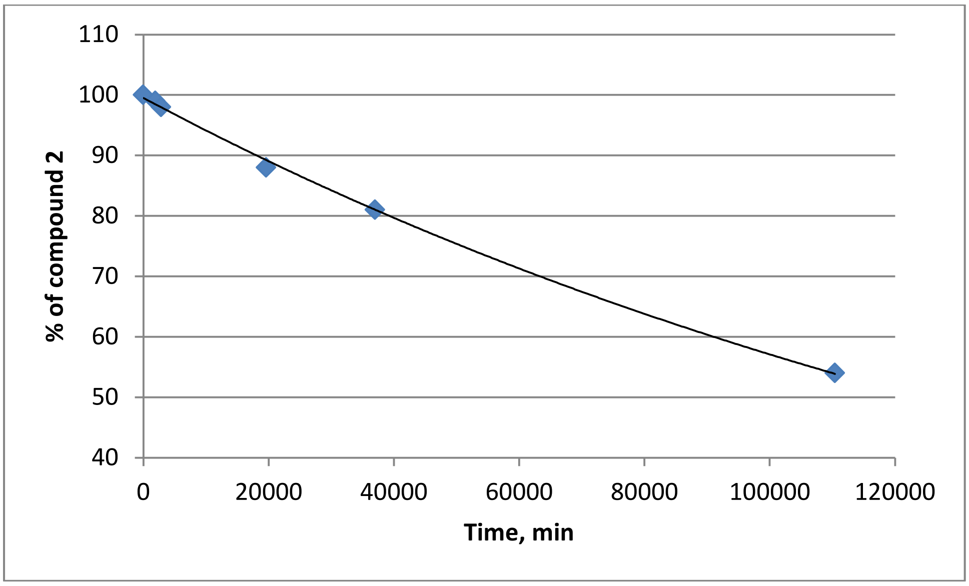

Figure S47. Exponential fit of the data (Table S11).

Table S12. Fitting parameters and corresponding kinetic constants.

Equation	y0	A1	t1	R^2	Half-life, $t_{1/2}$, min	k_{obs}, min^{-1}	$\log(k_{obs})$
y = A1·exp(-x/t1) + y0	15,58528± 9,53246	84,25563± 9,34923	140851,4166± 23258,65209	0,9986	126114	~$5.496 \cdot 10^{-6}$	-5.260

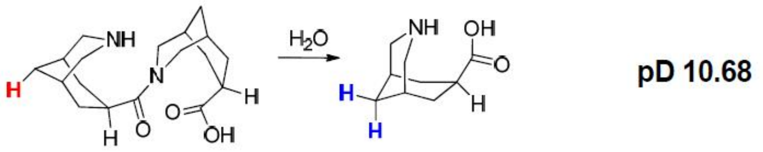

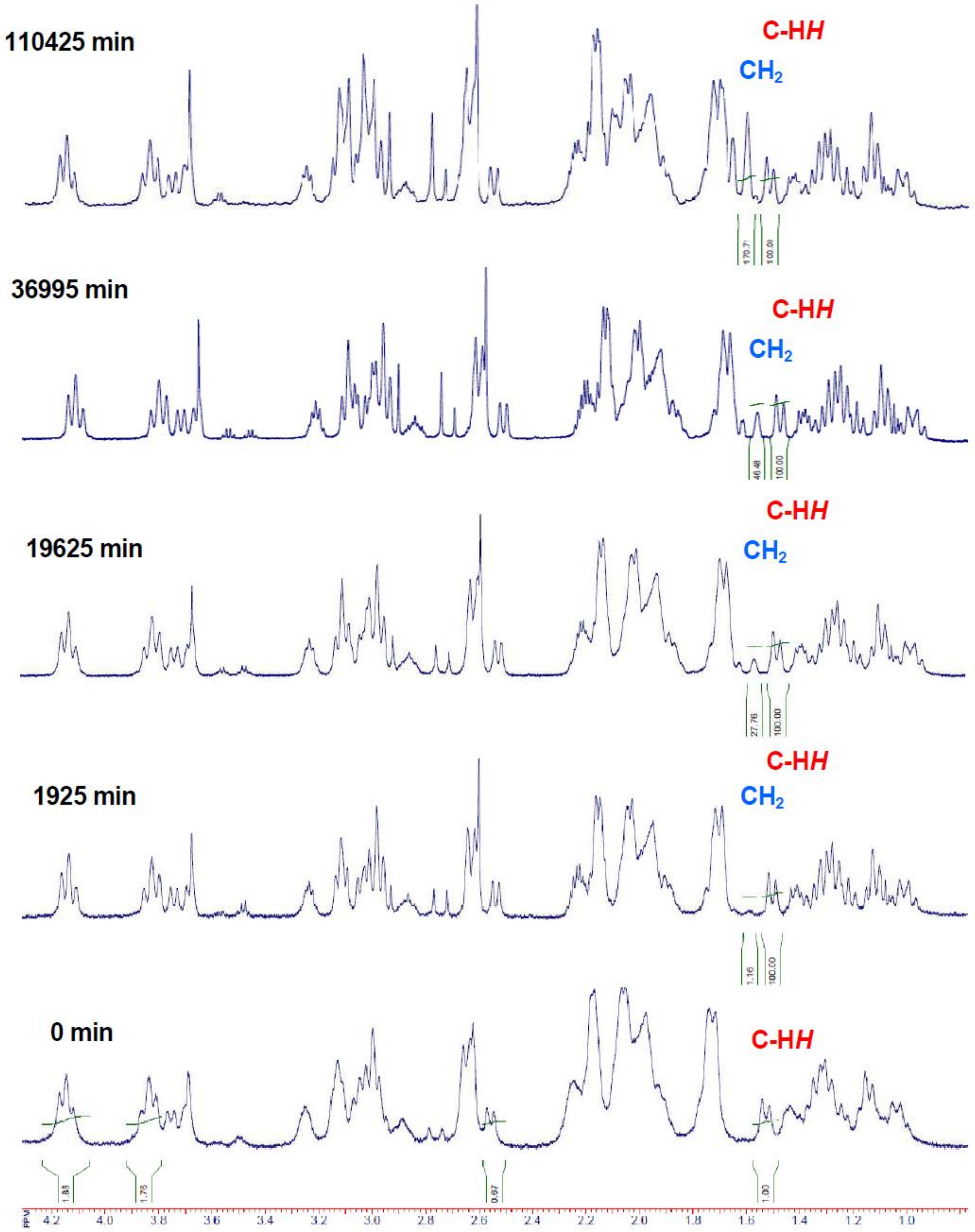

Figure S48. Representative spectral data set for hydrolysis of **2**·HCl in phosphate D_2O buffer, pD 10.68, 23 °C.

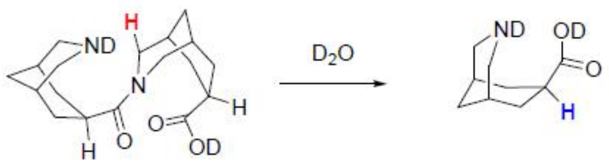

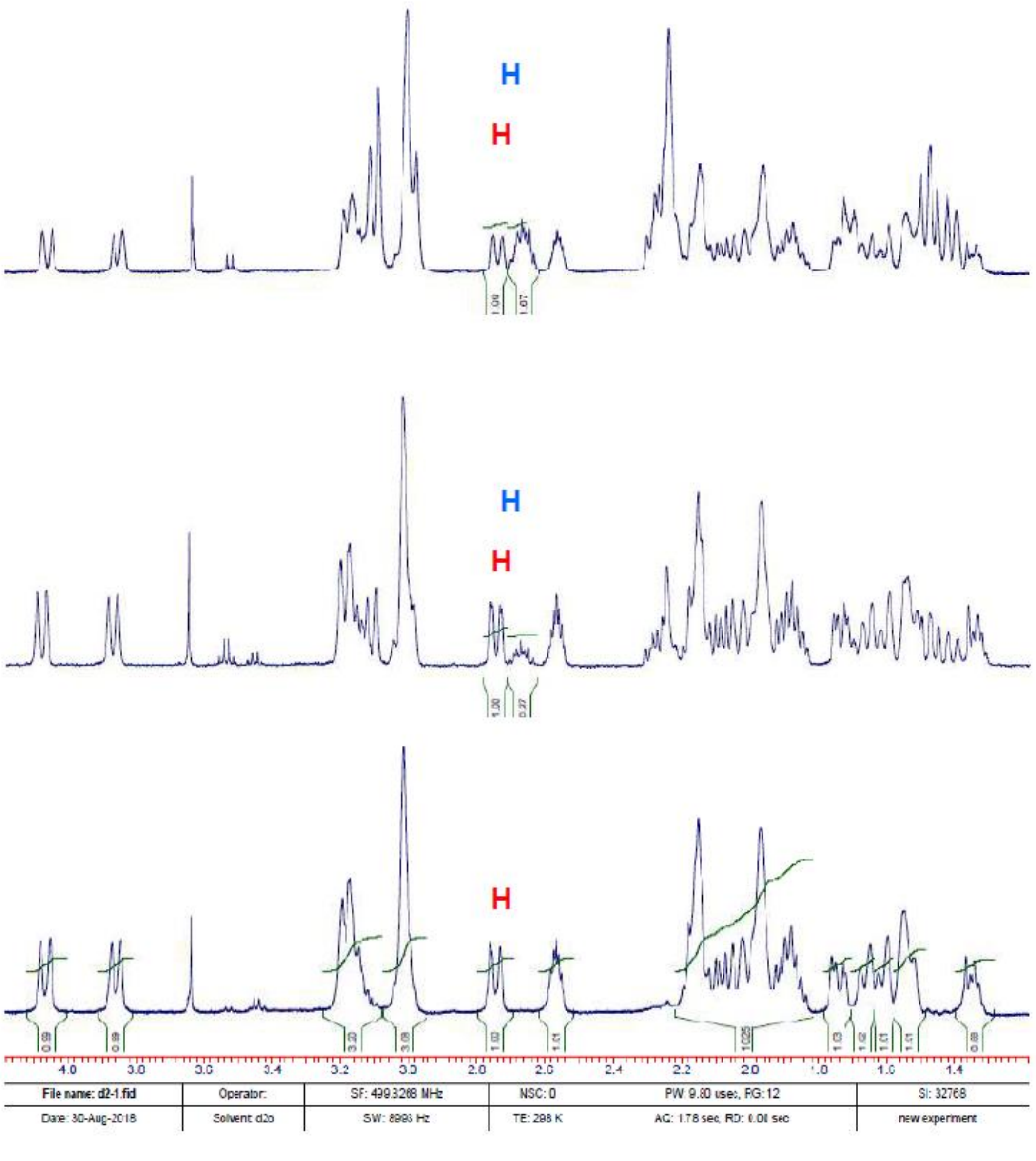

Figure S49. Representative ^{1}H-NMR spectral data set for hydrolysis of **2**·HCl in D_2O (experiment run for estimation of the isotope kinetic effect), 23 °C.

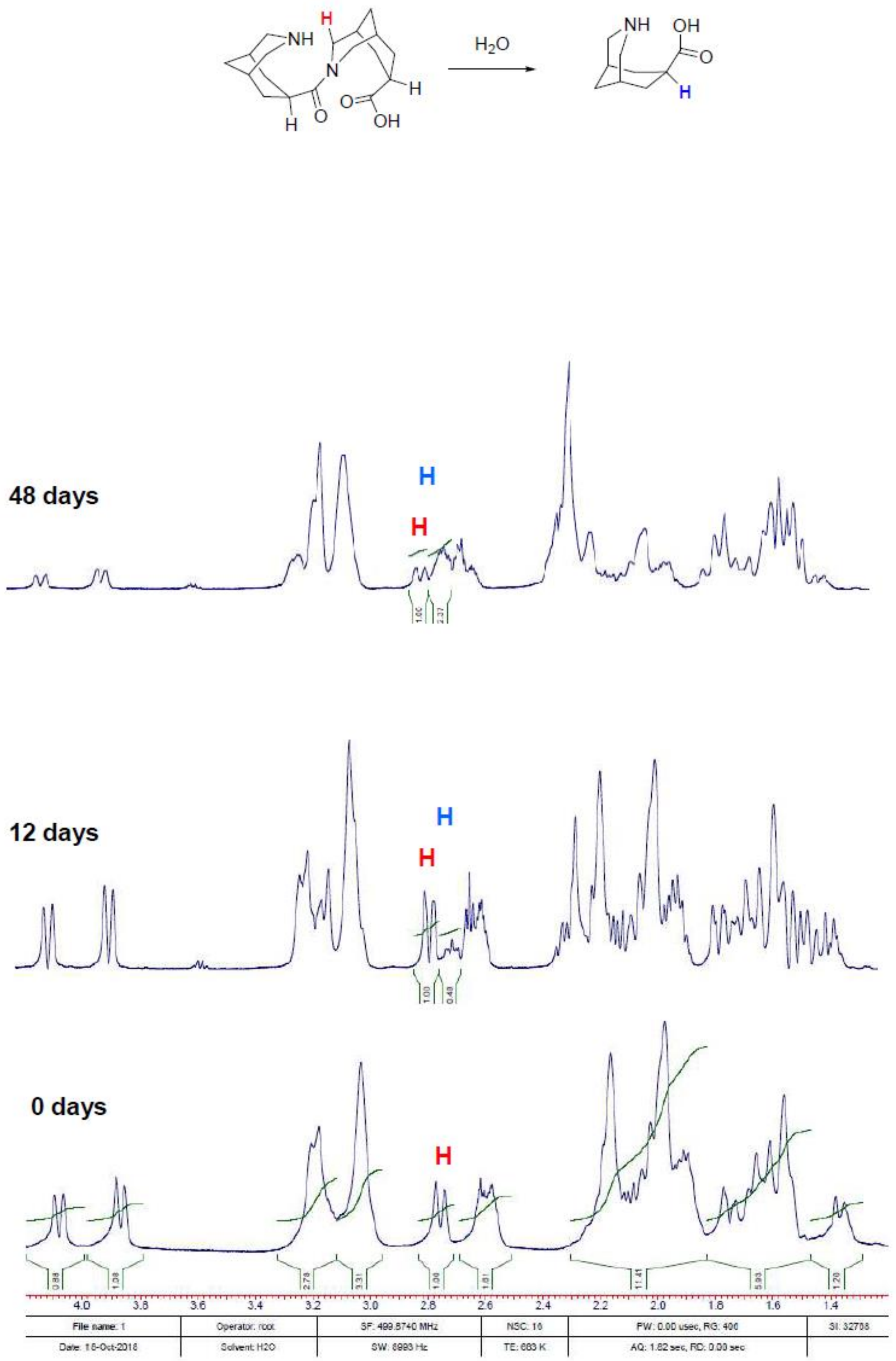

Figure S50. Representative ^{1}H-NMR spectral data set for hydrolysis of **2**·HCl in H_2O (experiment run for estimation of the isotope kinetic effect), 23 °C.
